# HaploMaker: An improved algorithm for rapid haplotype assembly of genomic sequences

**DOI:** 10.1093/gigascience/giac038

**Published:** 2022-05-17

**Authors:** Mario Fruzangohar, William A Timmins, Olena Kravchuk, Julian Taylor

**Affiliations:** The Biometry Hub, School of Agriculture, Food and Wine & Waite Research Institute, University of Adelaide, Glen Osmond, South Australia, 5064, Australia; The Biometry Hub, School of Agriculture, Food and Wine & Waite Research Institute, University of Adelaide, Glen Osmond, South Australia, 5064, Australia; The Biometry Hub, School of Agriculture, Food and Wine & Waite Research Institute, University of Adelaide, Glen Osmond, South Australia, 5064, Australia; The Biometry Hub, School of Agriculture, Food and Wine & Waite Research Institute, University of Adelaide, Glen Osmond, South Australia, 5064, Australia

**Keywords:** haplotype, PacBio, CCS, HiFi, CLR, Illumina, paired-end, SNP, INDEL, heterozygous

## Abstract

**Background:**

In diploid organisms, whole-genome haplotype assembly relies on the accurate identification and assignment of heterozygous single-nucleotide polymorphism alleles to the correct homologous chromosomes. This appropriate phasing of these alleles ensures that combinations of single-nucleotide polymorphisms on any chromosome, called haplotypes, can then be used in downstream genetic analysis approaches including determining their potential association with important phenotypic traits. A number of statistical algorithms and complementary computational software tools have been developed for whole-genome haplotype construction from genomic sequence data. However, many algorithms lack the ability to phase long haplotype blocks and simultaneously achieve a competitive accuracy.

**Results:**

In this research we present HaploMaker, a novel reference-based haplotype assembly algorithm capable of accurately and efficiently phasing long haplotypes using paired-end short reads and longer Pacific Biosciences reads from diploid genomic sequences. To achieve this we frame the problem as a directed acyclic graph with edges weighted on read evidence and use efficient path traversal and minimization techniques to optimally phase haplotypes. We compared the HaploMaker algorithm with 3 other common reference-based haplotype assembly tools using public haplotype data of human individuals from the Platinum Genome project. With short-read sequences, the HaploMaker algorithm maintained a competitively low switch error rate across all haplotype lengths and was superior in phasing longer genomic regions. For longer Pacific Biosciences reads, the phasing accuracy of HaploMaker remained competitive for all block lengths and generated substantially longer block lengths than the competing algorithms.

**Conclusions:**

HaploMaker provides an improved haplotype assembly algorithm for diploid genomic sequences by accurately phasing longer haplotypes. The computationally efficient and portable nature of the Java implementation of the algorithm will ensure that it has maximal impact in reference-sequence–based haplotype assembly applications.

## Introduction

Diploid organisms such as human, *Arabidopsis thaliana*, barley, and many other eukaryotic genomes typically contain 2 homologous copies of every chromosome, each inherited from either of the parents. When there is allelic variation (wild type or mutant) at 1 genomic position between homologous chromosomes, the position is called heterozygous. For a single heterozygous site it is possible to quantify the number of wild-type and mutant alleles by using, for example, variant-calling tools. However, when 2 or more heterozygous sites are present, it is not possible to determine whether their alleles are on the same or different chromosomes. The presence of certain alleles from multiple genomic positions on 1 chromosome is called a haplotype and may be associated with an important phenotypic trait in 1 or more individuals. In contrast, if the alleles reside on different chromosomes, this can be connected to a loss of function [1]. This distinction results in the need for computationally efficient algorithms that can accurately phase alleles on 1 chromosome using sequencing reads obtained from diploid organisms [2].

There are several approaches to haplotype construction [3]. One group of construction methods relies heavily on using sequence information obtained from multiple related individuals of a population. Haplotypes can then be phased and assembled using various aspects of the genomic structure of the population, such as linkage disequilibrium [4, 5]. Other construction approaches in this group have focussed on using founder sequences and inferring haplotype phase through identity by descent [6] or the use of hidden Markov models [7–9] Unfortunately, these approaches are generally not applicable for haplotype phasing and assembly of sequences from a single sample when there are no additional genomic sequences available from related individuals.

This research focusses on the group of reference-based haplotype-phasing algorithms used when only DNA sequences of an individual sample are available [1]. For an individual diploid organism, there should be only 2 haplotypes detected. However, owing to sequencing errors and misalignments of reads to the reference, the total number of inferred haplotypes can potentially increase exponentially. To minimize errors in the haplotype construction, mathematical algorithms involving selection criteria are required to assist in determining the appropriate phase of the haplotypes. One strategy is to use the widely established minimum error correction (MEC) criterion [1, 10], where an optimal MEC indicates the smallest set of single-nucleotide polymorphism (SNP) changes that create a conflict-free separation of mapped reads into 2 groups. HapCUT [11], HapCUT2 [12], and SDhaP [13] belong to this category because they reconstruct a pair of haplotypes such that the fragments are maximally consistent with the assembled haplotypes. An extension of the MEC was proposed in WhatsHap [14], where a weighted MEC criterion was used and the optimization of haplotype construction is achieved through dynamic programming. In HapCompass [15] the problem of haplotype construction in polyploids and diploids is defined as an undirected weighted graph and an algorithm is developed that incorporates cycle basis local optimizations for resolving conflicting evidence. HapTree [2] focusses on polyploid species and develops a Bayesian maximum-likelihood framework for haplotype phasing and construction. We note here that the necessity of having a specific method for polyploid species has lessened nowadays because newer versions of genomic references (e.g., tetraploid wheat) contain all homoeologous copies of 1 chromosome.

In this research we present a novel reference-based algorithm for phasing 2 haplotypes (paternal/maternal) of a single diploid organism by using its genomic sequence reads. The algorithm frames the haplotype construction problem as a directed acyclic graph (DAG) structure and determines the optimal haplotype assembly using minimal path and related graph traversal algorithms [16]. We call this algorithm "HaploMaker." The algorithm attempts to phase longer DNA strands as long as any heterozygous position within a strand, and ≥1 other nearby heterozygous position, is covered by a single DNA fragment. The algorithm provides support for phasing SNPs as well as potentially important insertion/deletion (INDELs) polymorphisms. Computationally, the HaploMaker algorithm has reduced execution times and only requires a minimal amount of memory.

For demonstrating and benchmarking the HaploMaker algorithm, we have chosen the human pedigree of 17 individuals (2 parents, 11 children, and 4 grandparents) from 3 generations [17], where their DNA has been sequenced and haplotypes of 2 parents have been verified using inheritance constraints in the pedigree and the concordance of variant calls across different methods. We compared HaploMaker results with those generated by HapCUT2, HapCompass, and WhatsHap. WhatsHap was selected owing to its widespread use in assembling haplotypes [18], and HapCUT2 and HapCompass were selected owing to recent reports of their ability to generate accurate haplotype assemblies [15]. We showed that when using paired-end short reads with 10× or 25× coverage, compared to the competitors used in this research, the HaploMaker algorithm was capable of constructing longer haplotype blocks while maintaining a competitively low switch error rate. Additionally, when using longer Pacific Biosciences (PacBio) continuous long reads (CLRs) or more accurate PacBio HiFi reads, HaploMaker generated substantially longer haplotype blocks, ensuring a more complete assembled genome.

## Methods

### Data preparation

#### Individual NA12877 paired-end reads

The paired-end FastQ files for NA12877 of the Platinum Genome project were downloaded from [19] and the NA12877 phased VCF file was downloaded from [20]. The FastQ files were then sampled randomly to 10× and 25× genome coverages and the resulting 162.6 and 407.5 million paired reads from the FastQ files were mapped to the human genome reference version 38 using Bowtie 2 [21], allowing for a 1% mismatch rate and capturing short INDELs up to 20 bases (see Supplemental Material).

#### Individual NA12878 PacBio CLR/subreads

The sorted bam file of PacBio reads was downloaded from [22, 23]. Similar to individual NA12877, the corresponding VCF file was downloaded from the Platinum Genome project. For mapping of the PacBio reads we used the human genome reference hg19 downloaded from [24].

#### Individual NA12878 PacBio HiFi reads

High-quality PacBio reads (known as CCS or HiFi) related to individual NA12878 were downloaded from the NCBI SRA archive (accession No. SRX5780566) [3]. For mapping of the reads we used the human genome reference hg19 from the PacBio subread experiment. The corresponding VCF file from the PacBio subread experiment was also used.

#### Haplotype directed acyclic graph

To simplify the notation and development of the theoretical framework, we initially focus on a single chromosome with the understanding that the framework will apply identically to other chromosomes. From the VCF file, assume *L* heterozygous alleles or variants have been detected at various positions on the chromosome, with each allele pair assigned a random phase. We now formalize a framework for accurate phasing of these alleles using a DAG and the aligned read sequence evidence.

Let $\mathcal{G}$ define a haplotype directed acyclic graph (H-DAG), such that $\mathcal{G}\ = \ ( {V,E} )$, where

-
$V\ = {\mathrm{\{ }}(v_1^1,v_2^1{\mathrm{)}}\ ,\ \ldots ,\ (v_1^l,\ v_2^l),\ \ldots ,\ (v_1^L,\ v_2^L)\} \ $ defines the complete set of nodes (or vertices); $(v_1^l,\ v_2^l)\ $ are considered a sibling pair of nodes at the *l*th level of the graph.
-
$E = \{ {( {{v^\delta },\ {v^{\delta + 1}}} )\ |\ {v^\delta } \in v_i^\delta ,\ {v^{\delta + 1}} \in v_j^{\delta + 1}, i,j = \ 1,2,\ \forall \ \delta \ = \ 1, \ldots ,\ L - 1} \} $ defines the set of all possible edges between *L* nodes in *V*.

**Figure 1:**
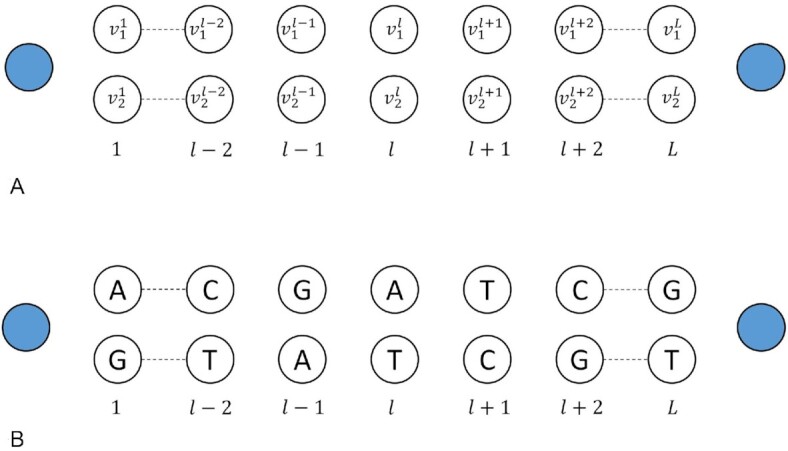
(A) Skeleton of the H-DAG with *L* pairs of nodes and 2 dummy nodes capping each end of the graph. (B) Skeleton of the H-DAG showing the random assignment of heterozygous pairs of alleles to each pair of nodes.

The sibling paired node structure for a H-DAG with *L* levels is visually represented in Fig. 1A with the complete skeleton H-DAG containing 2 dummy nodes at the beginning and end of the graph. Using this node structure, and without additional read sequence evidence, the *L* ordered pairs of heterozygous alleles are sequentially assigned to pairs of nodes. This assignment is represented in Fig. 1B, where, e.g., at the *l*th level, the pair of nodes have been assigned alleles $v_1^l = \ {\mathrm{A}}$ and $v_2^l = \ {\mathrm{T}}$ in the skeleton H-DAG.

The definition of the H-DAG edges indicates that directed edges can only exist between adjacent levels of the graph, ensuring that no directed cycles are possible. Additionally, for any directed edge, $( {{v^\delta },\ {v^{\delta + 1}}} ) \in E$, between arbitrary adjacent levels, ${v^\delta }$ is explicitly the parent node of ${v^{\delta + 1}}$ and ${v^{\delta + 1}}$ is explicitly the child node of ${v^\delta }$. This implies that directed edges can only occur left to right (low to high levels) sequentially across the H-DAG. Without additional evidence from the sequencing reads, the ambiguity of allelic phase at any level of the graph suggests that all directed edges are possible. In the following sections we discuss using evidence from the DNA reads to refine the rules of generating directed edges across the adjacent levels of the H-DAG.

### Continuous and discontinuous DNA fragments

**Figure 2:**
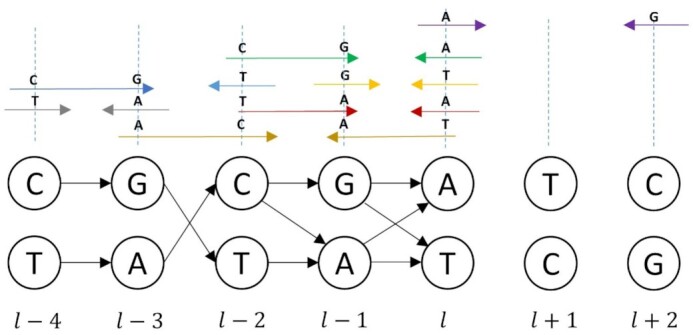
H-DAG with generated directed edges based on evidence from continuous DNA fragments (arrows above nodes from levels $l - 4, \ldots ,\ l$) that span ≥2 consecutive levels. Purple paired-end reads are considered discontinuous because they span non-consecutive levels.

Once the heterozygous alleles are assigned to the skeleton H-DAG, the HaploMaker algorithm is ready to generate directed edges sequentially across the levels of the graph using evidence from the DNA reads. To illustrate the development of this component of the algorithm we have focussed on paired-end reads, but a similar argument applies for longer single-end reads. During the processing of the read evidence, a DNA fragment (matching left and right paired-end reads) was considered to be continuous if it spanned consecutive levels of the H-DAG without loss of coverage across any of the heterozygous alleles contained in those levels. This is exemplified in Fig. 2, where the $( {l - 4,\ \ldots ,\ l + 2} )$ levels of the example skeleton H-DAG from Fig. 1 are used. The paired-end reads (arrows above nodes) are considered continuous DNA fragments because they span ≥2 consecutive levels $( {l - 4, \ldots ,\ l} )$ of the H-DAG with read evidence indicating that they also contain the heterozygous alleles in those levels.

In contrast, a DNA fragment was considered to be discontinuous if it spanned non-consecutive levels of the H-DAG. Discontinuities such as this are common and can arise when the reference DNA fragment insert size exceeds the aggregate size of the left and right paired-end read lengths. Figure 2 provides a simplified example of this where the purple end reads are considered discontinuous because they span levels $( {l,\ l + 1,l + 2} )$ with a discontinuity, indicating a lack of coverage across the $( {l + 1} )\mathrm{ th}$ level.

### Directed edges for continuous DNA fragments

Initially, the HaploMaker algorithm builds directed edges sequentially across levels of the H-DAG spanned by the continuous fragments. Owing to the initial random assignation of allelic phase to any pair of nodes in the skeleton H-DAG, and the potential of DNA misalignment or sequencing errors, the number of directed edges constructed between any 2 consecutive levels of the H-DAG can vary. Consider the node framework for arbitrary adjacent levels $\delta $ and $\delta + 1$ of the H-DAG and let the set of edges between these levels be defined by ${E^\delta }$. On the basis of continuous DNA fragment evidence, 4 distinct scenarios are possible for the consecutive allele pairs:

1. Unambiguous in phase: ${E^\delta } = \ \{ {( {{v^\delta },\ {v^{\delta + 1}}} )\ |\ {v^\delta } \in v_i^\delta ,\ {v^{\delta + 1}} \in v_i^{\delta + 1},\ i\ = \ 1,2} \}$. This indicates that there is read evidence that the allele pairs are in phase with no ambiguity and this supports the generation of 2 straight directed edges (not crossing over) from the parent nodes to child nodes.

2. Unambiguous out of phase: ${E^\delta } = \{ {( {{v^\delta },\ {v^{\delta + 1}}} )\ |\ {v^\delta } \in v_i^\delta ,\ {v^{\delta + 1}} \in v_j^{\delta + 1},\ i,j = 1,2;\ i \ne j} \}\ $. This indicates that there is read evidence that the allele pairs are out of phase with no ambiguity, and this supports the generation of 2 directed edges that cross over from the parent nodes to the child nodes.

3. Ambiguous phase of 1 allele pair: ${E^\delta } = \{ {( {{v^\delta },\ {v^{\delta + 1}}} )\ |\ {v^\delta } \in v_i^\delta ,\ {v^{\delta + 1}} \in v_j^{\delta + 1},\ i = 1,\ j = 1,2;i = j = 2} \}\ .$ This indicates that there is read evidence suggesting phase ambiguity of the allele pair at level $\delta $ of the graph. This supports the generation of 2 directed edges using Scenario 1 and a third diagonal directed edge from 1 parent node to a child node matching the read evidence.

4. Ambiguous phase of both allele pairs: ${E^\delta } = \ \{ {( {{v^\delta },\ {v^{\delta + 1}}} )\ |\ {v^\delta } \in v_i^\delta ,\ {v^{\delta + 1}} \in v_j^{\delta + 1},\ i,j\ = \ 1,2} \}$, indicating that there is read evidence that suggests phase ambiguity of both allele pairs and this supports the generation of 4 directed edges using Scenarios 1 and 2 defined above to match the conflicting read evidence.

Figure 2 provides a visual representation of the directed edge types spanning the $( {l - 4,\ \ldots ,\ l} )$ consecutive levels of an example H-DAG with the supporting read evidence above the graph nodes. It should be noted that the mirror version of Scenario 3 is also possible but has been omitted for brevity.

### Induced directed edges for discontinuous fragments

After generating all the directed edges based on evidence from the continuous DNA fragments, the H-DAG will most likely contain discontinuities or no directed edges between some adjacent levels. When paired-end read evidence indicates that there are discontinuous DNA fragments spanning these levels (see Fig. 2), then the HaploMaker algorithm builds new "induced directed edges" to connect the source node containing the heterozygous allele in the left paired-end read to the target node containing the heterozygous allele in the right paired-end read. For cases where there are overlapping discontinuous fragments the algorithm processes each of them sequentially by the order of their target node levels (from low to high levels) and if 2 or more fragments have the same target level then the processing is ordered by their source node level (from high to low levels). After processing fragments their induced edges immediately become part of the pre-existing directed edge framework for the H-DAG.

To process an individual discontinuous fragment, we designate the source node as the initial parent node and induce new edges between subsequent levels of the H-DAG using a “look-ahead” pre-order depth first traversal approach [25, 26]. The first component of the approach involves a single-level look-ahead algorithm that uses knowledge of the pre-existing directed edges between the source and target nodes to induce new edges to potential children in the adjacent levels of the H-DAG. Once the directed edges are built, the pre-order depth traversal algorithm then visits each of the children, assigns them as parents, and the process is repeated until the target level is encountered.

Owing to the simplistic nature of the H-DAG framework, the look-ahead algorithm to find potential children nodes and induce new edges can be easily defined for any 2 adjacent levels within the source node and target node defined by the discontinuous fragment. Suppose read evidence indicated that the source node was $\ v_i^l$, with source node sibling $v_j^l,\ i,j \in 1,2\ j \ne i$, and traversal was required to target node $v_k^m$, where $m - l > 1$ and $k \in 1,2$. Additionally, let ${E^{lm}}$ be the set of pre-existing directed edges between levels *l* and *m* of the H-DAG. During the recursive depth traversal, consider a parent node $v_i^\delta $ where $\ i \in 1,2$ and $l \le \delta < m$; then we can define an edge-inducing algorithm between adjacent levels $\delta $ and $\delta + 1$ that respects pre-existing edges and ambiguity of read evidence, namely:

**getPotentialChildren** (parent node $v_i^\delta $, sibling node $v_j^\delta $)

**if**  $v_j^\delta $ has an observed allele AND has directed edges to both child nodes at level $\delta + 1$  **then**

update ${E^{lm}}$ to include directed edges from parent node $v_i^\delta $ to both nodes at level $\ \delta + 1$

**else if**  $v_j^\delta $ does not have an observed allele OR has 0 or 1 edge to child nodes at level $\delta + 1$  **then**

**for** any child node at level $\delta + 1\ $ with no parent AND is not the sibling of the target node

update ${E^{lm}}$ to include a directed edge from the parent node $v_i^\delta $ to that child node.

**end for**

**end if**

**Figure 3:**
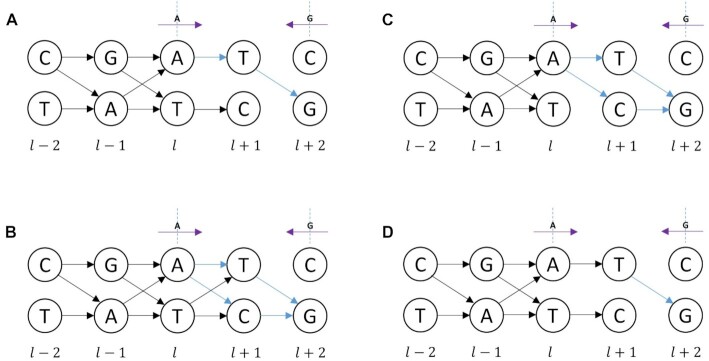
Various H-DAG possibilities with induced directed edges linking the discontinuous purple DNA fragment from the source node containing allele A at level *l* to target node containing allele G at level $l + 2$. Black edges are pre-existing directed edges; blue edges are new induced directed edges using the getPotentialChildren algorithm.

Given pre-existing edges in the H-DAG, Fig. 3 presents various examples of how the "getPotentialChildren" algorithm induces new edges between a source node and target node. In Fig. 3A, a previously known directed edge exists between alleles T and C at the *l* and $l + 1$ levels of the graph, suggesting that there is unambiguous phase of the allelic pairs between these levels. The getPotentialChildren algorithm indicates that we then require a directed edge from the source node with allele A at level *l* to allele T at the $l + 1$ level to match the unambiguous phase. Using pre-order depth traversal, the node containing allele T becomes the new parent node and a single induced directed edge is generated from T to the target node containing allele G to align with the read evidence from the discontinuous fragment. In Fig. 3B the pre-existing directed edges indicate that there is phase ambiguity of the allelic pairs between levels *l* and $l + 1$. The getPotentialChildren algorithm then induces directed edges from the source node containing allele A to both nodes at level $l + 1$. The pre-order depth traversal algorithm then sequentially assumes that each node at level $l + 1$ is a parent node and generates a directed edge from each of the nodes to the target node. Figure 3C follows identically to Fig. 3B due to the phase ambiguity of the allelic pairs between levels *l* and $l + 1$ and Fig. 3D follows identically from Fig. 3A.

The combined pre-order depth traversal, along with the getPotentialChildren method, is then repeated for each discontinuous DNA fragment along the H-DAG. Once complete, the H-DAG has obtained maximum connectivity between levels based on the complete DNA evidence from continuous and discontinuous fragments. Within this final H-DAG, disconnected levels may still exist where there is lack of read evidence to generate directed edges. As a consequence the H-DAG may be partitioned into several sub-graphs based on distinct genomic "blocks," where each block contains consecutive levels of the original H-DAG with ≥1 directed edge between each level. Without loss of generality an H-DAG block will be defined as a sub-graph ${\mathcal{G}_b} = ( {{V_b},{E_b}} )\ $ containing $\ {L_b}$ levels. The remaining algorithmic sections discuss an approach for the numerical estimation of edge weights in a general H-DAG block as well as the path traversal optimization technique to appropriately phase the heterozygous alleles within each block.

### Estimating directed edge weights

For each of the directed edges within an H-DAG block, we now derive a locally based probabilistic edge weight function based on cumulative DNA evidence and ambiguity of allelic phase between adjacent levels. Let $v_{i\ }^\delta \in {V_b}$ be a source node within an H-DAG block and $e_{ij\ }^\delta = ( {v_{i\ }^\delta ,\ v_{j\ }^{\delta + 1}} )\ \in {E_b},\ 1 \le \delta < L,\ i,j \in 1,2\ $ be an *existing* directed edge between any 2 adjacent levels. We then define $n( {e_{ij\ }^\delta } )$ to be its edge counter, initialized at 1, and incremented by 1 every time $e_{ij\ }^\delta $ is spanned by a DNA fragment. This initialization provides a convenient mechanism for ascribing a minimum numerical value for induced directed edges generated from discontinuous DNA fragments.

We then define a general probabilistic edge weight function as

(1) \begin{eqnarray*}
w_{ij}^\delta = \mathrm{ Pr}\ \left( {e_{ij\ }^\delta } \right) = \frac{{n\left( {e_{ij\ }^\delta } \right)}}{{\mathop \sum \nolimits_k n\left( {e_{ik\ }^\delta } \right)}}, \end{eqnarray*}

where the denominator $\mathop \sum \nolimits_k n( {e_{ik\ }^\delta } )$ represents the sum of the existing edge counters emitting from the source node. When there is only 1 directed edge emitting from the source node, e.g., where there is an unambiguous phase between adjacent pairs, the numerator and denominator of this edge weight become identical and $w_{ij}^\delta = \ 1$. This immediately indicates that edge weights <1 are derived from adjacent phase-ambiguous allelic pairs only.

To exemplify the ambiguous allele pairs case for continuous DNA fragments, consider the $l - 1$ and *l* levels of the H-DAG in Fig. 3. The source node $v_2^{l - 1}$ containing allele A emits 2 directed edges, $e_{21}^l$ and $e_{22}^l$. Let $n( {e_{21}^l} )$ and $n( {e_{22}^l} )$ indicate their associated edge counts based on the cumulative read evidence for the existence of each directed edge. Using equation (1) the 2 edge weights are simply estimated by the local probabilities

\begin{equation*} {\mathrm{\ }}w_{21}^l = \frac{{n\left( {e_{21}^l} \right)}}{{n\left( {e_{21}^l} \right) + n\left( {e_{22}^l} \right)}}\ ,\ \ w_{22}^l = \frac{{n\left( {e_{22}^l} \right)}}{{n\left( {e_{21}^l} \right) + n\left( {e_{22}^l} \right)}}\
\end{equation*}

and have the property, $w_{21}^l + w_{22}^l\ = \ 1$. Similarly, in Fig. 3C between the *l* and $l + 1$ levels of the H-DAG there are induced edges formed between a discontinuous DNA fragment, and the 2 edges emitting from source node $v_1^l\ $ indicate some phase ambiguity between the allele pairs. Because there are no DNA fragments spanning the edges, the counters $n(w_{12}^{l + 1})$ and $n(w_{11}^{l + 1})$ would both remain at their initial value of 1. Using equation (1), this immediately indicates that the induced edge weights are $w_{12}^{l + 1} = \ \ w_{11}^{l + 1} = \ 0.5$ and this probabilistic value would be assigned to all pairs of induced edges emitting from the same parent node between adjacent levels in the H-DAG.

### Minimum weighted path

Once the calculation of all edge weights is complete, a minimum weighted path can be algorithmically determined. Let ${P_b} = \{ {{p_1},\ {p_2},\ \ldots ,\ {p_t}} \}\ \ $ be the complete set of distinct paths through the block. For any path ${p_k} \in {P_b}$, a unique set of nodes ${V_{b:k\ }} \subset {V_b}$, such that ${V_{b:k\ }} = \ \{ {v_k^1,v_k^2,\ \ldots ,\ v_k^{{L_b}}} \}$, are visited across the ${L_b}$ levels, where, at any level of the sub-graph, ${l_b}$ say, $v_k^{{l_b}}$ is 1 of the nodes from the pair $(v_1^{{l_b}},v_2^{{l_b}})$. Similar to the complete graph, for the purpose of optimization, the sub-graph is also capped at each end with dummy nodes (Start*_k_*, End*_k_*). As the path ${p_k}$ traverses across a unique set of nodes, it also comprises a unique set of directed edges defined by ${E_{b:k\ }} \subset {E_b}$, where ${E_{b:k\ }} = \{ {( {\mathrm{ Star}{\mathrm{ t}_k},v_k^1} ),(v_k^1,v_k^2), \ldots ,(v_k^{{L_b} - 1},v_k^{{L_b}}),(v_k^{{L_b}},\mathrm{ En}{\mathrm{ d}_k})} \}\ = \ \{ {( {\mathrm{ Star}{\mathrm{ t}_k},v_k^1} ),e_k^1,\ \ldots ,\ e_k^{{L_b} - 1},(v_k^{{L_b}},\mathrm{ En}{\mathrm{ d}_k})} \}$. We define the likelihood of this path as

(2) \begin{eqnarray*}
Q\left( {{p_k}} \right)\ = \ \mathrm{ Pr}\ \left( {{p_k}} \right) = \mathop \prod \nolimits_{{l_b} = 1}^{{L_b} - 1} \mathrm{ Pr}\left( {e_k^{{l_b}}} \right)\ = \mathop \prod \nolimits_{{l_b} = 1}^{{L_b} - 1} w_k^{{l_b}}, \end{eqnarray*}

where $w_k^{{l_b}} = \ \mathrm{ Pr}( {e_k^{{l_b}}} )$, the local probability or weight of the directed edge that traverses from level ${l_b} - 1$ to level ${l_b}$ of the H-DAG block. Determining the appropriate phase of the haplotype within the H-DAG block is then equivalent to finding the path with maximum likelihood over the complete set of paths.

For the purpose of utilizing a known path traversal algorithm we can equivalently frame this optimization as a minimization problem. Let ${S_b} = \ \{ {{s_1},\ {s_2},\ \ldots ,\ {s_t}} \}\ $ be a set of values for the complete set of path traversals through the H-DAG block such that $S\ ( {{p_k}} ) = \ - \log Q( {{p_k}} )$. Determining the optimal path through the H-DAG block is then equivalent to finding the minimum negative log-likelihood path over the complete set of paths, namely,

(3) \begin{eqnarray*}
\mathop {\min }\nolimits_{{s_k} \in {S_b}} \{ S\left( {{p_k}} \right),k = 1, \ldots ,t;S\left( {{p_k}} \right) = \mathop \sum \nolimits_{{l_b} = 1}^{{L_b} - 1} v_k^{{l_b}}\}, \end{eqnarray*}

where $v_k^{{l_b}} = \ - \log ( {w_k^{{l_b}}} )\ \ge \ 0\ \forall \ k,\ {l_b}$ define the directed edge weights used in the optimization algorithm. Optimization of equation (3) can then equivalently be viewed as finding the path of minimum weight through the H-DAG block. Because the H-DAG block has no directed cycles it can be immediately topologically sorted and we can then use an established backtracking algorithm to perform the optimization [16]. This backtracking algorithm is also known to be efficient, requiring $O( {n( {{E_b}} ) + n( {{V_b}} )} )$ linear time, where $n( {{E_b}} )$ and $n( {{V_b}} )$ are the number of directed edges and nodes in the H-DAG block.

**Figure 4:**
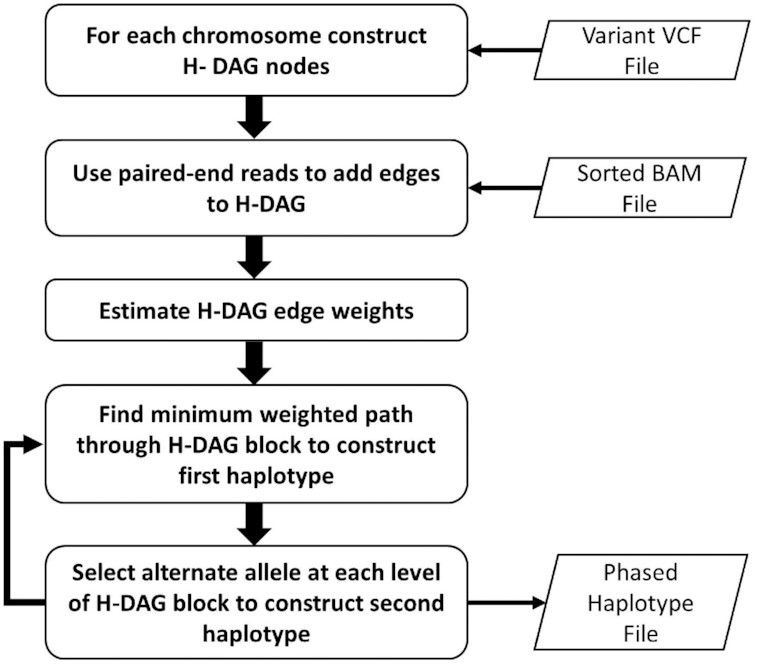
Workflow schematic of the HaploMaker algorithm.

After the backtracking algorithm completes, the first haplotype is obtained by traversing through the H-DAG block on the minimum weighted path and selecting 1 node at each level on the path. The corresponding second haplotype is then obtained by traversing the minimum weighted path and selecting the alternate allele (the allele not on the path) at each level of the H-DAG. The backtracking algorithmic process is then repeated for each H-DAG block. The algorithm halts once the minimum weighted path is obtained for the final H-DAG block containing the right-hand dummy node for the chromosome. Figure 4 presents a flow chart of the complete HaploMaker algorithm for each chromosome from the initial variant calling through to the repeated construction of 2 sequences for each haplotype block.

### Algorithm accuracy and statistics

To assess the accuracy of the algorithms to correctly phase the alleles for each haplotype, we used the well-known “switch error” accuracy measure. For any haplotype, the switch error is defined as the number of times the predicted haplotype allele is disconcordant with the true allele obtained from the phased VCF file. The switch error is then averaged as the number of errors per megabase pair. As HaploMaker attempts to construct longer haplotypes through inducing new edges, other useful statistics for comparing algorithms were also calculated. These included the well-known N50, the mean haplotype length in megabase pairs, and maximum haplotype length achievable with each algorithm. An R-based computational implementation of these statistics (comparison.R) is available at [27].

## Results

### Individual NA12877 paired-end reads

The human reference genome version 38 was used to map 162.6 million (10×) and 407.5 million (25×) paired-end reads from NA12877 individuals with 83.6% and 83.9% of reads, respectively, aligned concordantly. The mean gap-compressed sequence dissimilarity rate was estimated at 0.25%, and this was similar to previous reports of heterozygosity levels in humans [28]. A total of 1.86% of reads contained small INDELs of ≤20 bp. The median insert size (DNA fragment length) was estimated to be 316 bp. The sorted BAM file, along with the NA12877 phased VCF file, was then given as input to HaploMaker and the comparative haplotype construction software, HapCompass, HapCUT2, and WhatsHap (see Supplemental Material for execution commands). All algorithms were computationally conducted using a cloud-based Linux instance with 2 cores and 32 GB RAM. HaploMaker and HapCompass were the most computationally expedient, with HapCUT2 and WhatsHap taking longer to execute.

An R script (comparison.R), available at [27], was used to process the output of the 4 haplotype algorithms. The switch error rate, the N50 of haplotype blocks, and other useful statistics were extracted and are presented in Table 1. The table demonstrates that HaploMaker had the lowest switch error rate compared to the other algorithms. For 10× data, HaploMaker, HapCompass, and WhatsHap generated equivalent maximum haplotype lengths of 2,770 bp. For 25× data, HaploMaker and HapCompass generated larger haplotype blocks (≤26,188 bp) compared to the other 2 algorithms. The table also indicates that HapCompass and WhatsHap had substantially higher switch error rates compared to HaploMaker and HapCUT2. Although HapCUT2 had a competitively low switch error rate, it also had shorter and fewer haplotype blocks.

**Table 1:** Comparison of various statistics obtained from the output of the 4 haplotype-phasing algorithms applied to individual NA12877 10× and 25× coverage short paired-end reads

Read coverage	Algorithm	Switch error rate (per Mb)	N50 (bp)	Haplotype length (bp)		Running time (min)
				Mean	Maximum	
10×	HaploMaker	32.1	326	251	2,770	10
	HapCompass	143.9	329	246	2,770	10
	HapCUT2	43.0	296	181	2,310	15
	WhatsHap	84.2	329	252	2,770	35
25×	HaploMaker	38.0	496	331	26,188	25
	HapCompass	157.9	494	326	26,188	30
	HapCUT2	47.6	420	269	5,985	30
	WhatsHap	77.2	459	307	14,339	135

Table 1 also indicates that the increase in read coverage to 25× had a negligible effect on the accuracy of all 4 algorithms with only a slight increase in the switch error rate. However, comparing the N50 and the mean and maximum haplotype length, increasing coverage definitively generated longer haplotype blocks, with HaploMaker and HapCompass generating the highest mean haplotype lengths. Maximum haplotype lengths exceeded 9 times the maximum haplotype length obtained from 10× read coverage.

**Figure 5:**
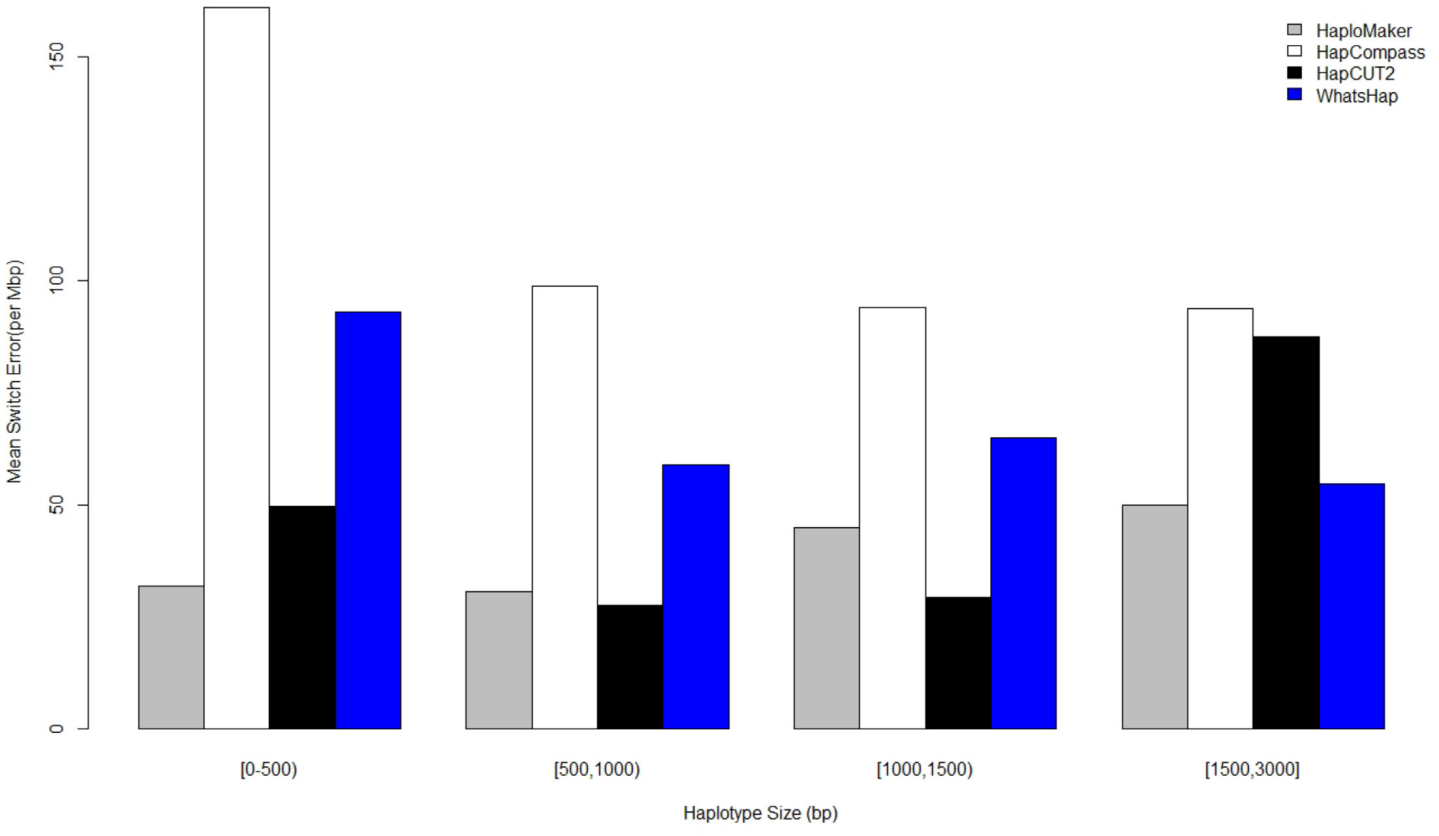
Comparison graph showing the average switch error rate for different haplotype length classes across all 4 haplotype-phasing algorithms applied to individual NA12877 using 10× short paired-end reads.

**Figure 6:**
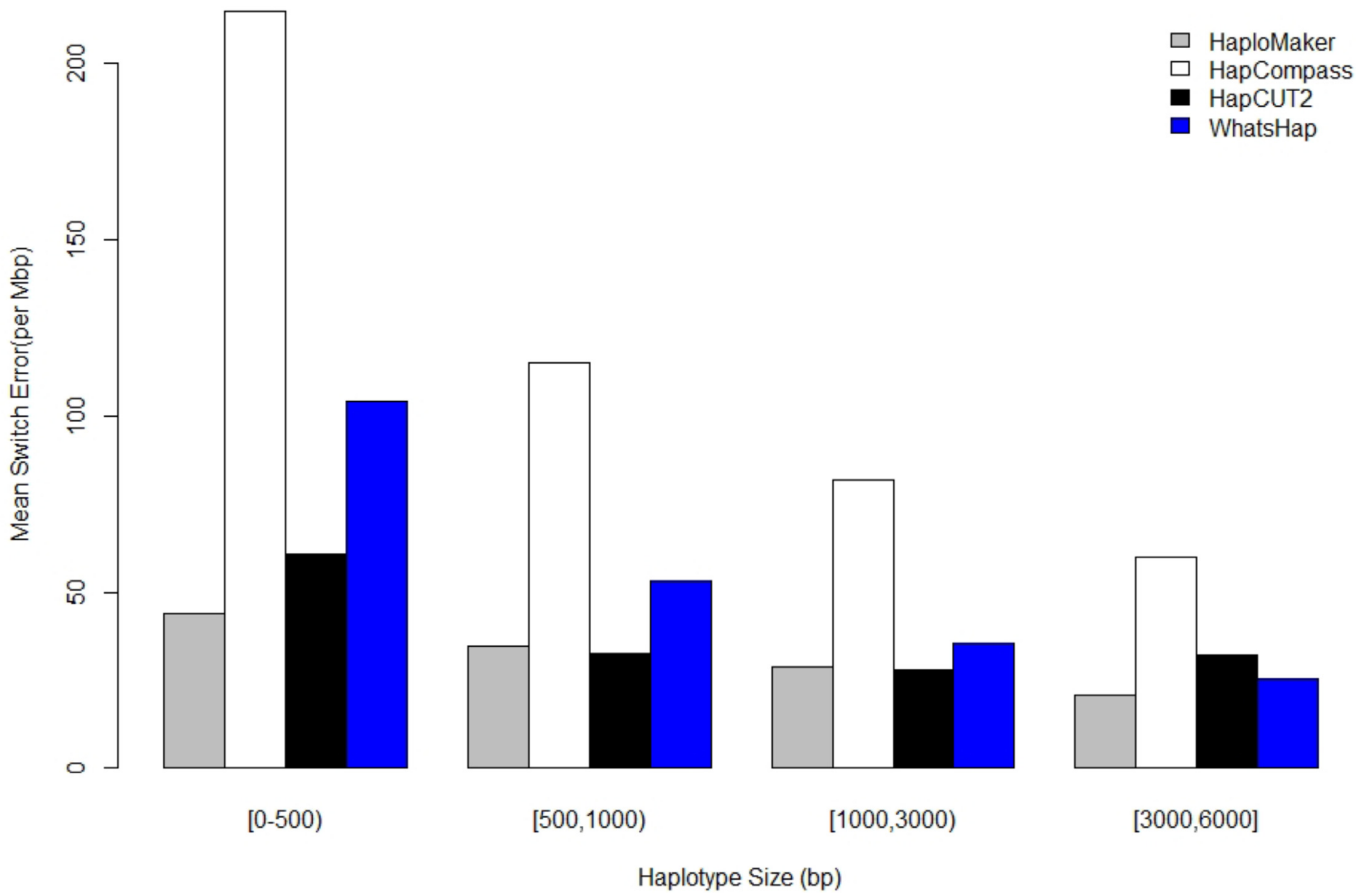
Mean switch error rate for different haplotype length classes across all 4 haplotype-phasing algorithms applied to individual NA12877 using 25× short paired-end reads.

To more rigorously assess the changes in accuracy as the haplotype block size increased, Figs 5 and 6 present the mean switch error rate of each of the algorithms against a class of haplotype lengths (in number of base pairs). The absolute numbers of haplotypes in each haplotype length class obtained from the 4 algorithms are given in Supplementary Tables S1 and S2 for the 10× and 25× read coverages, respectively. The figures revealed that, as haplotype blocks became longer, HaploMaker maintained a competitively low switch error rate compared to the other 3 algorithms, indicating that the HaploMaker algorithm preserves accuracy as the size of haplotypes increases. The switch error rate obtained from the HapCUT2 algorithm remained competitive against HaploMaker for shorter haplotype block lengths. However, when the haplotype block length increased to 1,500 and 3,000 bp at 10× and 25× coverage, respectively, the switch error rate of HapCUT2 became higher than that of HaploMaker. WhatsHap and HapCompass were the least competitive across all haplotype block size lengths.

### Individual NA12878 PacBio CLR/subreads

The total number of PacBio reads was 64 million, and their median and mean length was 3,611 and 5,005 bp, respectively. A total of 92% of reads contained INDELs, and their size varied from 1 up to 10,000 bp. The original alignment to the hg19 human genome reference was performed using BLASR [29] by the authors [22] with sequencing coverage estimated to be 65×. The sequencing error rate of PacBio subreads was reported to be ∼14% [30]. Owing to the lack of long-read PacBio-specific settings in HapCompass, we only compared HaploMaker with HapCUT2 and WhatsHap. We used a cloud-based instance of 4 cores and 32 GB of RAM. The computational implementation of the HaploMaker algorithm executed in a similar amount of time to HapCUT2 and 2.5 times faster than WhatsHap.

An R script (comparison2.R), available at [27], was used to process the output of the 3 haplotype-phasing algorithms. Table 2 contains the switch error rate, the N50 of haplotype blocks, and other useful statistics. The table indicates that HaploMaker was superior in generating longer haplotype blocks while maintaining a competitively low switch error rate. In particular, the N50 and mean haplotype length obtained from the HaploMaker algorithm was ≥30% longer than HapCUT2 and 300% longer than WhatsHap. These increased haplotype lengths obtained from HaploMaker also ensured greater coverage of the total human reference genome.

**Table 2:** Comparison of various statistics obtained from the output of the 3 haplotype-phasing algorithms applied to individual NA12878 PacBio subreads

Algorithm	Switch error rate (per Mb)	N50 (bp)	Mean haplotype length (bp)	Total genome coverage (Gb)	Maximum haplotype length (bp)	Running time (min)
HaploMaker	27.4	46,787	25,490	1.73	351,891	90
HapCUT2	2.0	37,251	19,560	1.66	299,882	167
WhatsHap	21.0	14,828	4,737	1.23	265,020	415

**Figure 7:**
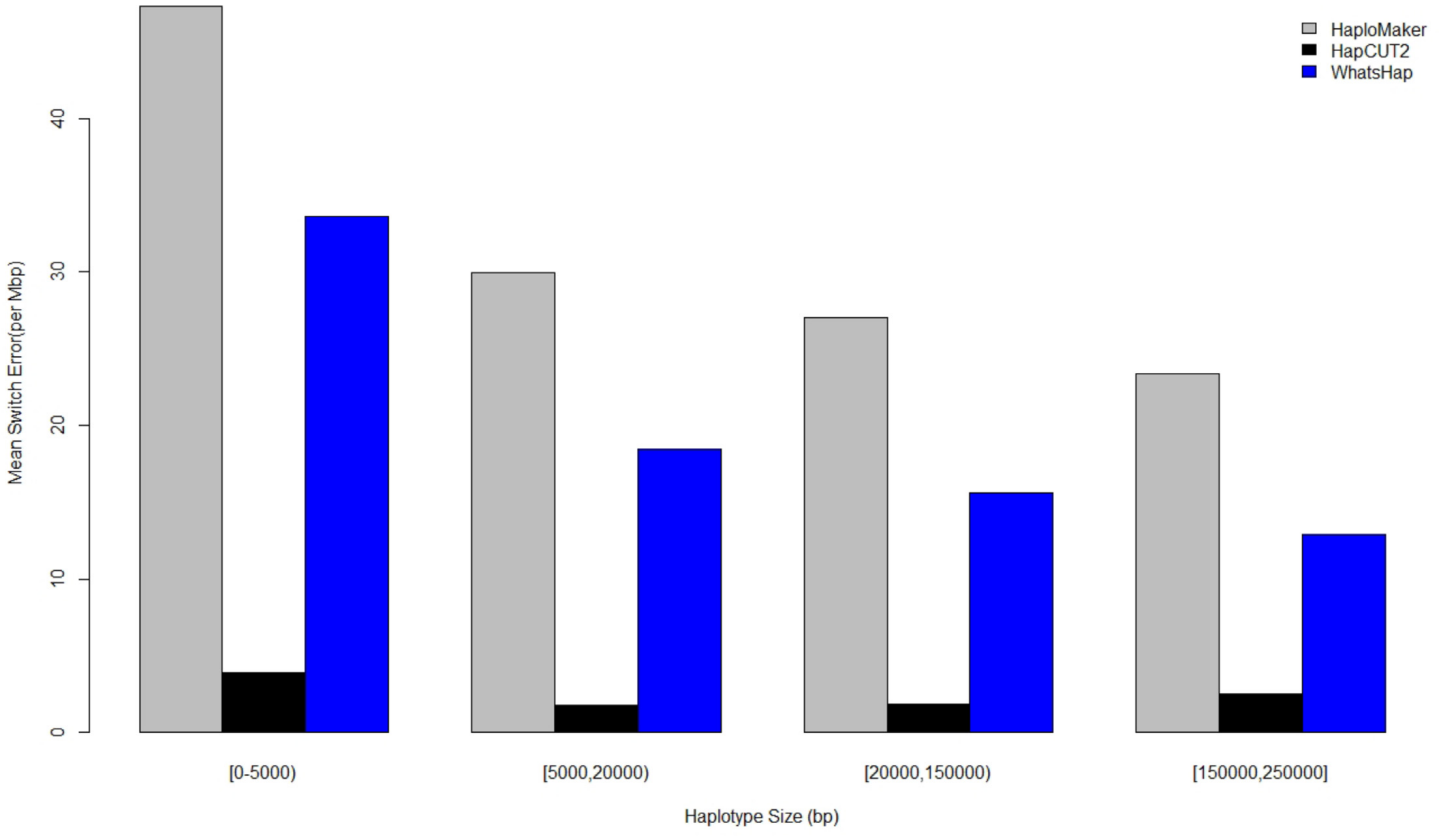
Comparison graph showing the average switch error rate of different haplotype length groups for 3 haplotype-phasing algorithms applied to individual NA12878 PacBio subreads.

Compared to HaploMaker, HapCUT2 had a significantly lower switch error rate and it maintained this reduced rate throughout the partitioned class of haplotype lengths (Fig. 7). In this figure we only compared blocks ≤250 kb because HaploMaker was the only algorithm to be able to generate longer blocks >250 kb (see Supplementary Table S3). Table S3 indicates almost a 2-fold increase in the number of short haplotype block lengths generated by HapCUT2 compared to HaploMaker, suggesting that for sequencing reads with high error rates, the HaploMaker algorithm trades off some accuracy while it attempts to generate longer haplotype blocks.

### Individual NA12878 PacBio HiFi reads

A total of 1.5 million PacBio HiFi reads with a mean length of 10 kb were mapped to the human genome reference hg19 using the pbmm2 aligner from PacBio [31]. The mean gap-compressed sequence identity was 98.6%, and this was closer to human genome polymorphism compared to the PacBio subreads. Sequencing coverage was estimated ∼5× and the accuracy of HiFi reads was ∼99.8%. This is much higher than the accuracy of traditional PacBio subreads [32], and as a consequence the algorithms are expected to construct more accurate haplotype blocks. All 3 algorithms were executed using identical hardware used to analyse the PacBio subread data. Because there were much fewer reads compared to previous experiments, all 3 algorithms had reduced execution times. HaploMaker completed in 5 minutes, with HapCUT2 and WhatsHap completing in 14 and 18 minutes, respectively (see Supplemental Material for execution commands).

An R script (comparison3.R), available at [27], was used to process the output of the 3 haplotype-phasing algorithms. and Table 3 contains the switch error rate and other useful output statistics. Owing to the sequence accuracy of the HiFi reads, the switch error rates of all 3 methods were significantly lower compared to the switch error rates obtained from analysing the PacBio subreads and paired-end read data sets. Despite reduced sequencing coverage, HaploMaker managed to construct ≥2 times longer mean haplotype lengths and N50 than the competing algorithms. As a result HaploMaker assembled a 1.54-Gb genome compared to 1.34 and 1.14 Gb genomes generated from the 2 other algorithms, a gain of ≤35% coverage of the human genome.

**Table 3:** Comparison of various statistics obtained from the output of the 3 haplotype-phasing algorithms applied to individual NA12878 PacBio HiFi reads

Algorithm	Switch error rate (per Mb)	N50 (bp)	Mean haplotype length (bp)	Total genome coverage (Gb)	Maximum haplotype length (bp)	Running time (min)
HaploMaker	2.6	31,698	15,340	1.54	315,905	5
HapCUT2	1.4	14,081	6,816	1.34	140,731	14
WhatsHap	4.5	12,542	4,369	1.14	183,560	18

**Figure 8:**
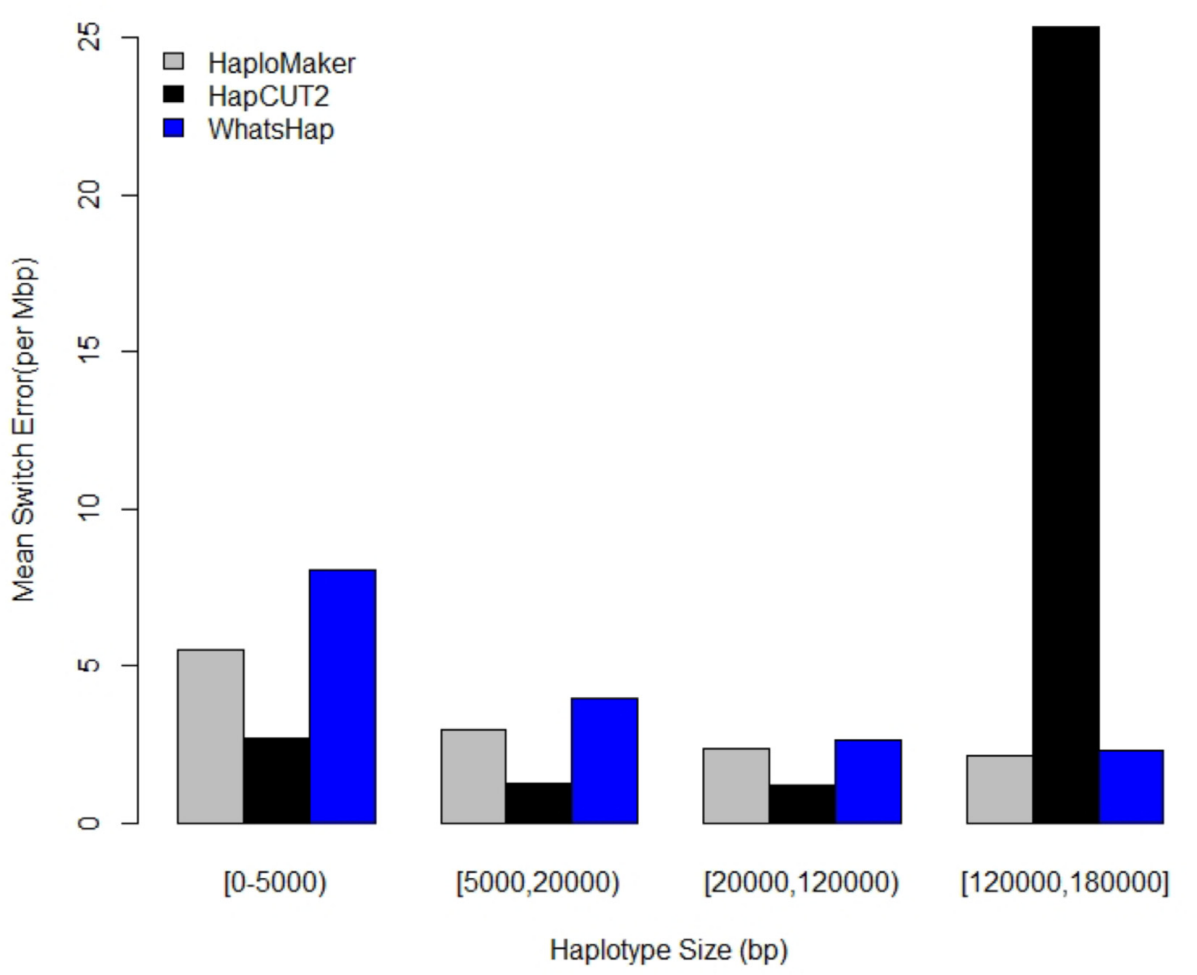
Mean switch error rate of different haplotype length groups for 3 haplotype-phasing algorithms applied to individual NA12878 PacBio HiFi reads.

Figure 8 presents the switch error rate of each of the algorithms against the haplotype length, with the number of haplotype lengths in each class given in Supplementary Table S4. In the figure we only compared blocks ≤180 kb because HaploMaker was the only algorithm able to generate longer blocks >180 kb (see Supplementary Table S4). The figure indicates that both HaploMaker and WhatsHap maintained low switch error rates across all length classes, with HaploMaker being more accurate. HapCUT2 had the lowest switch error rate up to 120 kb but then generated a significantly larger switch error rate (10× worse) for >120 kb haplotypes compared to the other 2 algorithms. Similar to the PacBio subread experiment, WhatsHap and HapCUT2 generated substantially more shorter haplotype blocks than HaploMaker. However, in contrast to the subread experiment, HaploMaker maintained a low switch error rate, suggesting that the quality of HaploMaker's haplotype-phasing algorithm is dramatically increased when the accuracy of the sequencing reads is improved.

## Discussion

In this research we presented an improved reference sequence–based haplotype construction algorithm, HaploMaker, that accurately assembles haplotypes of diploid genomic sequences by framing the problem as an edge-weighted DAG and phasing haplotypes using optimal path traversal algorithmic techniques. The novel strategy of inducing new directed edges based on read evidence to resolve disconnected levels of the H-DAG enabled HaploMaker to accurately phase longer genomic regions compared to other leading reference-based sequence algorithms, HapCUT2, HapCompass, and WhatsHap. For PacBio CLR data, the longer haplotype lengths generated by HaploMaker resulted in an increased switch error rate compared to the other algorithms, suggesting that HaploMaker trades off accuracy for haplotype length when read sequencing error is high. However, when longer and more accurate PacBio HiFi reads were used, the mean haplotype block lengths assembled were substantially greater using HaploMaker with, negligible sacrifice in accuracy. In all experiments analysed here, longer block lengths generated by the algorithm also ensured greater coverage of the genome. From a computational standpoint, the HaploMaker algorithm was shown to scale well, with a substantial reduction in computing time when longer, more computationally intensive PacBio reads were used. It is also important to note that the phasing of INDEL polymorphisms has been incorporated into the HaploMaker algorithm and it maintained a highly competitive accuracy. This is a crucial aspect of the algorithm because INDELs were the major source of switch errors from the haplotype construction software compared here and this issue is also exacerbated when read sequences, such as PacBio subreads, have a high sequencing error rate [12].

Graph-based frameworks have been widely adopted for population and reference-based haplotype assembly of genomes [7, 15, 33]. In this research we focussed on defining the problem as a simplistic DAG that removes the bulk of the complexity that many graph-based haplotype-phasing algorithms contain [15, 34]. The look-ahead edge-inducing algorithm contained in HaploMaker bears resemblance to the divide and conquer strategy used in the spectral graph–based approach of [33] except HaploMaker reconnects pairs of vertices when there is sufficient read evidence to do so, resulting in longer haplotype blocks. When read sequencing error is high this generation of longer haplotypes comes at the cost of reduced accuracy, and we are currently exploring small amendments to the HaploMaker algorithm to ensure that the accuracy remains competitive.

The length of assembled haplotypes depends on level of genome heterozygosity, DNA read/fragment length, and coverage of sequencing used. The human genome has a significantly lower level of heterozygosity compared to other diploid organisms such as *A. thaliana* [35]. Therefore we can expect shorter assembled haplotypes from human genomic sequences. In our example, using DNA fragments (insert size) of 316 bp and a paired-end sequencing coverage of 10× and 25×, the HaploMaker algorithm achieved a maximum haplotype length of 2,770 and 26,188 bp, respectively. While these lengths are short relative to the total length of a human chromosome, it is sufficient to enable PCR primer and CRISPR/Cas9 guide RNA-based experiments where a short homologue-specific sequence around a genomic position is required [36, 37]. In contrast, when using PacBio with read lengths of 5 kb average, the HaploMaker algorithm managed to assemble haplotypes up to 350 kb. This result emphasizes the importance of sequencing longer reads if a greater haplotype length is required [1].

We have demonstrated the ability of the HaploMaker algorithm to accurately assemble human diploid genomic sequences, and its potential is now being explored for other areas of related genomic sequence research. For example, the algorithm could be used to assemble haplotypes for genomes from tetraploid and hexaploid species, such as wheat, as long as there are separate reference sequences for each of the homoeologous copies of the chromosomes [1]. In cases where a set of genomic sequences have been generated from a population of related individuals, we are exploring the use of the base algorithm of HaploMaker for discovering the most recurring haplotypes among the population.

## Conclusion

By framing the haplotype assembly problem as a DAG and using a novel edge-inducing strategy for discontinuous DNA fragments, the HaploMaker algorithm was able to accurately phase long haplotype blocks using short or long sequence reads. The algorithm was shown to be highly efficient and also has potential to have an impact in similar genomic sequence research areas where accurate haplotype phasing or selection is required. To ensure the portability of the HaploMaker algorithm across varying computing architectures it has been implemented in Java and is available under MIT license from [27].

## Data Availability

The 2 FastQ files and the sorted BAM file relating to the NA12877 individuals are accessible through the NCBI SRA [38]. The VCF files relating to the individuals NA12877 and NA12878 are publicly accessible through the Figshare repository [39]. The human genome reference and its Bowtie2 index were downloaded from the link within [40].

The Bowtie read alignment software version 2.4.1 was downloaded from [41]. Comparative haplotype assembly software, HapCompass version 0.8.2, was downloaded from [42] and required Java 1.8 or higher to execute. HapCUT2 1.3.3 was compiled from [43] and for execution required installation of high-throughput sequencing tools library htslib from [44]. WhatsHap version 1.1 (latest) depends on Python version 3.6 or higher and C++ compiler and was installed using pip. pbmm2 version 1.7 was downloaded and installed from [31].

## Availability of Source Code and Requirements

The HaploMaker source code and Java executable (MFbio.jar file) are publicly accessible from [27] under MIT license. The resulting haplotype output files for all 4 algorithms are accessible from Figshare [39]. R code to process the output files and generate reports is located at [27] in comparison.R, comparison2.R, and comparison3.R.

An archival copy of the code and supporting data is available via the *GigaScience* database, GigaDB [45].

Project Name: HaploMaker
Project Home Page: https://github.com/mfruzan/HaploMaker
Operating System: Platform independent
Programming Language: Java
Requirements: Java 1.8 or higher
License: MIT
RRID:SCR_021928
Biotools ID: haplomaker

## Additional Files

**Supplemental Material**Contains execution commands for mapping sequences to the human genome, execution commands for all haplotype phasing algorithms, Tables S1-S4.

**Supplementary Table S1**. Frequency of haplotypes across four haplotype length classes obtained from the four haplotype phasing algorithms applied to individual NA12877 10x coverage short paired end reads.

**Supplementary Table S2**. Frequency of haplotypes across four haplotype length classes obtained from the four haplotype phasing algorithms applied to individual NA12877 25x coverage short paired end reads.

**Supplementary Table S3**. Frequency of haplotypes across four haplotype length classes obtained from the three haplotype phasing algorithms applied to individual NA12877 PacBio subreads.

**Supplementary Table S4**. Frequency of haplotypes across four haplotype length classes obtained from the three haplotype phasing algorithms applied to individual NA12877 PacBio HiFi reads.

giac038_GIGA-D-21-00252_Original_Submission

giac038_GIGA-D-21-00252_Revision_1

giac038_GIGA-D-21-00252_Revision_2

giac038_GIGA-D-21-00252_Revision_3

giac038_Response_to_Reviewer_Comments_Original_Submission

giac038_Response_to_Reviewer_Comments_Revision_1

giac038_Response_to_Reviewer_Comments_Revision_2

giac038_Reviewer_1_Report_Original_Submission Shilpa Garg -- 10/11/2021 Reviewed

giac038_Reviewer_1_Report_Revision_1 Shilpa Garg -- 1/28/2022 Reviewed

giac038_Reviewer_2_Report_Original_Submission Alexander SchÃ¶nhuth D -- 10/20/2021 Reviewed

giac038_Reviewer_2_Report_Revision_1 Alexander SchÃ¶nhuth -- 1/31/2022 Reviewed

giac038_Reviewer_3_Report_Original_Submission Yuri Pirola -- 10/21/2021 Reviewed

giac038_Reviewer_3_Report_Revision_1 Yuri Pirola -- 1/28/2022 Reviewed

giac038_Supplemental_File

## Abbreviations

BLASR: Basic Local Alignment with Successive Refinement; bp: base pairs; CCS: circular consensus sequencing; CLR: continuous long reads; CRISPR: Clustered regularly interspaced short palindromic repeat; DAG: directed acyclic graph; Gb: gigibase pairs; H-DAG: haplotype DAG; HiFi: High Fidelity; INDEL: insertion deletion; kb: kilobase pairs; Mb: megabase pairs; MEC: minimum error correction; NCBI: National Center for Biotechnology Information; PacBio: Pacific Biosciences; RAM: random access memory; SNP: single-nucleotide polymorphism; SRA: Sequence Read Archive; VCF: Variant Calling Format.

## Competing Interests

The authors declare that they have no competing interests.

## Authors' Contributions

M.F. and J.T. designed the algorithm. All authors wrote the manuscript. M.F. wrote the source code in Java. M.F. and J.T. wrote the source code in R. M.F. performed the analyses.
