# Supplementary material for: HaploMaker: An improved algorithm for rapid haplotype assembly of genomic sequences
Source: Gigascience. 2022 May 17;11:giac038. doi: 10.1093/gigascience/giac038 (PMC9112781; doi:10.1093/gigascience/giac038)
Supplement: giac038_GIGA-D-21-00252_Original_Submission [file giac038_giga-d-21-00252_original_submission.pdf]

GigaScience

HaploMiner: An improved algorithm for rapid haplotype assembly of genomic sequences

--Manuscript Draft--

**Manuscript Number:**	GIGA-D-21-00252
**Full Title:**	HaploMiner: An improved algorithm for rapid haplotype assembly of genomic sequences
**Article Type:**	Research
**Funding Information:**	Grains Research and Development Corporation — Dr Olena Kravchuk
**Abstract:**	Background<br><br>In diploid organisms, whole genome haplotype assembly relies on the accurate identification and assignment of heterozygous single nucleotide polymorphism (SNP) alleles to the correct homologous chromosomes. This appropriate phasing of the alleles ensures combinations of SNPs on any chromosome, called haplotypes, can then be used in down-stream genetic analyses approaches including determining their potential association with important phenotypic traits. A number of statistical algorithms and complementary computational software tools have been developed for whole genome haplotype construction from genomic sequence data. However, many algorithms lack the ability to phase long haplotype blocks and simultaneously achieve a competitive accuracy.<br><br>Results<br><br>In this research we present HaploMiner, a novel reference-based haplotype assembly algorithm capable of accurately and efficiently phasing long haplotypes using paired-end short reads and longer PacBio reads from diploid genomic sequences. To achieve this we frame the problem as a directed acyclic graph with edges weighted on read evidence and use efficient path traversal and minimization techniques to optimally phase haplotypes. We compared the HaploMiner algorithm with three other common reference-based haplotype assembly tools using public haplotype data of human individuals from the Platinum Genome project. With short read sequences, the HaploMiner algorithm maintained a competitively low switch error rate across all haplotype lengths and was found to be superior in phasing longer genomic regions. For longer PacBio reads, HaploMiner remained competitive for small block lengths and managed to generate longer block lengths than the competing algorithms.<br><br>Conclusions<br><br>HaploMiner provides an improved haplotype assembly algorithm for diploid genomic sequences by accurately phasing longer haplotypes. The computationally efficient and portable nature of the Java implementation of the algorithm will ensure it has maximal impact in reference-sequence based haplotype assembly applications.
**Corresponding Author:**	Mario Fruzangohar<br>The University of Adelaide<br>Urrbrae, SA 5064, SA AUSTRALIA
**Corresponding Author Secondary Information:**	
**Corresponding Author's Institution:**	The University of Adelaide
**Corresponding Author's Secondary Institution:**	
**First Author:**	Mario Fruzangohar
**First Author Secondary Information:**	
**Order of Authors:**	Mario Fruzangohar

Powered by Editorial Manager® and ProduXion Manager® from Aries Systems Corporation

	William A. Timmins
	Olena Kravchuk
	Julian Taylor
Order of Authors Secondary Information:	
Additional Information:	
**Question**	**Response**
Are you submitting this manuscript to a special series or article collection?	No
**Experimental design and statistics**<br><br>Full details of the experimental design and statistical methods used should be given in the Methods section, as detailed in our Minimum Standards Reporting Checklist. Information essential to interpreting the data presented should be made available in the figure legends.<br><br>Have you included all the information requested in your manuscript?	Yes
**Resources**<br><br>A description of all resources used, including antibodies, cell lines, animals and software tools, with enough information to allow them to be uniquely identified, should be included in the Methods section. Authors are strongly encouraged to cite Research Resource Identifiers (RRIDs) for antibodies, model organisms and tools, where possible.<br><br>Have you included the information requested as detailed in our Minimum Standards Reporting Checklist?	Yes
**Availability of data and materials**<br><br>All datasets and code on which the conclusions of the paper rely must be either included in your submission or deposited in publicly available repositories (where available and ethically	Yes

appropriate), referencing such data using a unique identifier in the references and in the “Availability of Data and Materials” section of your manuscript.<br><br>Have you have met the above requirement as detailed in our Minimum Standards Reporting Checklist?	

Powered by Editorial Manager® and ProduXion Manager® from Aries Systems Corporation

Manuscript
Click here to access/download;Manuscript;HaploMiner-GigaScience_V14_JT2.docx

HaploMiner: An improved algorithm for rapid haplotype assembly of genomic sequences

Mario Fruzangohar[1*], William A. Timmins[1], Olena Kravchuk[1], Julian Taylor[1]

[1]The Biometry Hub, School of Agriculture, Food and Wine, University of Adelaide, Australia

[*]Corresponding author

Mario Fruzangohar: mario.fruzangohar@adelaide.edu.au

William A. Timmins: andy.timmins@adelaide.edu.au

Olena Kravchuk: olena.kravchuk@adelaide.edu.au

Julian Taylor: julian.taylor@adelaide.edu.au

Abstract

Background

In diploid organisms, whole genome haplotype assembly relies on the accurate identification and assignment of heterozygous single nucleotide polymorphism (SNP) alleles to the correct homologous chromosomes. This appropriate phasing of the alleles ensures combinations of SNPs on any chromosome, called haplotypes, can then be used in down-stream genetic analyses approaches including determining their potential association with important phenotypic traits. A number of statistical algorithms and complementary computational software tools have been developed for whole genome haplotype construction from genomic sequence data. However, many algorithms lack the ability to phase long haplotype blocks and simultaneously achieve a competitive accuracy.

Results

In this research we present *HaploMiner*, a novel reference-based haplotype assembly algorithm capable of accurately and efficiently phasing long haplotypes using paired-end short reads and longer PacBio reads from diploid genomic sequences. To achieve this we frame the problem as a directed acyclic graph with edges weighted on read evidence and use efficient path traversal and minimization techniques to optimally phase haplotypes. We compared the HaploMiner algorithm with three other common reference-based haplotype assembly tools using public haplotype data of human individuals from the Platinum Genome project. With short read sequences, the HaploMiner algorithm maintained a competitively low switch error rate across all haplotype lengths and was found to be superior in phasing longer genomic regions. For longer PacBio reads, HaploMiner remained competitive for small block lengths and managed to generate longer block lengths than the competing algorithms.

Conclusions

HaploMiner provides an improved haplotype assembly algorithm for diploid genomic sequences by accurately phasing longer haplotypes. The computationally efficient and portable nature of the Java

implementation of the algorithm will ensure it has maximal impact in reference-sequence based haplotype assembly applications.

Keywords: haplotype, PacBio, Illumina, SNP, INDEL, heterozygous, DNA sequence, genome

Supplementary information

Supplementary information is available in Supplemental Material submitted with this document.

Introduction

Diploid organisms such as human, *Arabidopsis thaliana*, barley and many other eukaryotic genomes typically contain two homologous copies of every chromosome, each inherited from either of the parents. When there is allelic variation (wild type or mutant) at one genomic position between homologous chromosomes, the position is called heterozygous. For a single heterozygous site it is possible to quantify the number of wild type and mutant alleles by using, for example, variant calling tools. However, when two or more heterozygous sites are present, it is not possible to determine whether their alleles are on the same or different chromosomes. The presence of certain alleles from multiple genomic positions on one chromosome is called a haplotype and may be associated with an important phenotypic trait in one or more individuals. In contrast, if the alleles reside on different chromosomes, this can be connected to a loss of function. This distinction results in the need for computationally efficient algorithms that can accurately phase alleles on one chromosome using sequencing reads obtained from diploid organisms [1].

There are several approaches to haplotype construction. One group of construction methods relies heavily on using sequence information obtained from multiple related individuals of a population. Haplotypes can then be phased and assembled using various aspects of the genomic structure of the population such as linkage disequilibrium [2, 3]. Other construction approaches in this group have focussed on using founder sequences and inferring haplotype phase through identity by descent [4] or the use of Hidden Markov Models [5-7] Unfortunately, these approaches are generally not applicable

for haplotype phasing and assembly of sequences from a single sample when there are no additional genomic sequences available from related individuals.

This research focusses on the group of reference-based haplotype phasing algorithms used when only DNA sequences of an individual sample are available. For an individual diploid organism, there should be only two haplotypes detected. However, due to sequencing errors and misalignments of reads to the reference, the total number of inferred haplotypes can potentially increase exponentially. To minimize errors in the haplotype construction, mathematical algorithms involving selection criteria are required to assist in determining the appropriate phase of the haplotypes. One strategy is to use the widely established minimum error correction (MEC) criterion [8] where an optimal MEC indicates the smallest set of SNP changes that create a conflict-free separation of mapped reads into two groups. HapCUT [9] , HapCUT2 [10] and SDhaP [11] belong to this category as they reconstruct a pair of haplotypes such that the fragments are maximally consistent with the assembled haplotypes. An extension of the MEC was proposed in WhatsHap [12] where a weighted MEC (wMEC) criterion was used and the optimization of haplotype construction is achieved through dynamic programming. In HapCompass [13] the problem of haplotype construction in polyploids and diploids is defined as an undirected weighted graph and an algorithm is developed that incorporates cycle basis local optimizations for resolving conflicting evidence. HapTree [1] focusses on polyploid species and develops a Bayesian maximum-likelihood framework for haplotype phasing and construction. We note here that the necessity of having a specific method for polyploid species has lessened nowadays, as newer versions of genomic references (*e.g.* tetraploid wheat) contain all homoeologous copies of one chromosome.

In this research we present a novel reference-based algorithm for phasing two haplotypes (paternal/maternal) of a single diploid organism by using its genomic sequence reads. The algorithm transforms the haplotype construction problem into a directed acyclic graph (DAG) structure and determines the optimal haplotype assembly using minimal path and related graph traversal algorithms [14]. We call this algorithm *HaploMiner*. The algorithm has the ability to phase DNA strands of any length as long as there are heterozygous positions close enough to be covered by at least a single DNA fragment. The algorithm provides support for phasing SNPs as well as potentially important

Insertion/Deletion (INDELs) polymorphisms. Computationally, the HaploMiner algorithm runs in *O(n)* linear time and only requires a minimal amount of computational memory and processor power.

For demonstrating and benchmarking the HaploMiner algorithm, we have chosen the human pedigree of 17 individuals (two parents, 11 children and four grandparents) from three generations [15], where their DNA has been sequenced and haplotypes of two parents have been verified using inheritance constraints in the pedigree and the concordance of variant calls across different methods. We compared HaploMiner results with those generated by HapCUT2, HapCompass and WhatsHap. WhatsHap was selected due to its widespread use in assembling haplotypes [16] and HapCUT2 and HapCompass were selected due to recent reports of their ability to generate accurate haplotype assemblies[13]. We showed that when using paired-end short reads, HaploMiner algorithm was capable of constructing the most accurate haplotype blocks while maintaining length of haplotypes. Additionally, when using longer PacBio reads, HaploMiner generated significantly longer haplotype blocks while maintaining a competitively low switch error rate.

Methods

Data preparation

Individual NA12877 paired-end reads

The paired-end FastQ files for NA12877 of the Platinum Genome project were downloaded from https://www.ebi.ac.uk/ena/browser/view/PRJEB3381 and the NA12877 phased VCF file was downloaded from https://sapac.illumina.com/platinumgenomes.html. The FastQ files were then sampled randomly to 15X genome coverage and the resulting 162.6 million paired reads from the FastQ files were mapped to the human genome reference version 38 using Bowtie 2 [17] allowing for a 1% mismatch rate and capturing short INDELs up to 20 bases (see Supplemental Material).

Individual NA12878 PacBio reads

The sorted bam file of PacBio reads was downloaded from ftp://ftp-trace.ncbi.nlm.nih.gov/giab/ftp/data/NA12878/NA12878_PacBio_MtSinai [18]. Similar to individual

NA12877, the corresponding VCF file was downloaded from the Platinum Genome project. For mapping of the PacBio reads we used the human genome reference hg19 and this was downloaded from http://hgdownload.cse.ucsc.edu/goldenPath/hg19/bigZips/chromFa.tar.gz.

Haplotype directed acyclic graph (H-DAG)

For each of the chromosomes, we used a directed acyclic graph framework to define a haplotype directed acyclic graph (H-DAG) as $\mathcal{G} = (N, E, L)$ where N and E define the complete set of nodes and edges of $\mathcal{G}$ respectively, and L defines the number of levels used in the H-DAG. Specifically, the complete set of nodes were defined as $N = \{(N_1^1, N_2^1), \dots, (N_1^l, N_2^l), \dots (N_1^L, N_2^L)\}$ where (N_1^l, N_2^l) defines a pair of nodes at the lth level of the graph. This general skeleton framework for a H-DAG with L levels is presented in Figure 1A with the complete skeleton H-DAG containing two dummy nodes at the beginning and end of the graph. Assuming there are L heterozygous reference positions within the chromosome, then using the skeleton H-DAG framework, pairs of alleles called at each heterozygous reference position are then sequentially assigned to pairs of nodes. The random assignation of the allelic phase to any pair of nodes in the H-DAG suggests there may be as many as 2^L potential haplotype combinations. Figure 1B presents an example skeleton H-DAG where, for example, at the lth level, the pair of nodes have been assigned alleles $N_1^l =$ A and $N_2^l =$ T.

Figure 1. *(A) Skeleton of the H-DAG with L pairs of nodes and two dummy nodes capping each end of the graph. (B) Skeleton of the H-DAG showing the random assignation of reference heterozygous pairs of alleles to each pair of nodes.*

Continuous and discontinuous DNA fragments

Once the complete skeleton H-DAG is formed, the HaploMiner algorithm is ready to generate directed edges sequentially across the levels of the graph using evidence from the DNA reads. To illustrate the development of the algorithm we have focussed on paired end reads but a similar argument applies for longer single end reads. During the processing of the read evidence, a DNA fragment (a DNA read and its pair) was considered to be continuous if it spanned consecutive levels of the H-DAG without loss of

coverage across the heterozygous alleles contained in those levels. This is exemplified in Figure 2 where the $(l-2,\ldots,l+2)$ levels of the example skeleton H-DAG from Figure 1 are used. The red, green and yellow paired-end reads are considered continuous DNA fragments as they span consecutive levels $(l-2,l-1,l)$ of the H-DAG with read evidence indicating they also contain the heterozygous alleles in those levels.

In contrast, a DNA fragment was considered to be discontinuous if it spanned non-consecutive levels of the H-DAG. Discontinuities such as this are common and can arise when the reference DNA fragment insert size exceeds the aggregate size of the left and right paired-end read lengths. Figure 2 provides a simplified example of this where the purple end reads are considered discontinuous as they span levels $(l,l+1,l+2)$ with a discontinuity, indicating a lack of coverage over the $(l+1)th$ level.

Directed edges for continuous DNA fragments

Initially, the HaploMiner algorithm builds directed edges across levels of the H-DAG spanned by the continuous fragments. Due to the initial random assignation of allelic phase to any pair of nodes in the skeleton H-DAG, and the potential of DNA misalignment or sequencing errors, the number of directed edges constructed between any two consecutive levels varies. In general, we define any single directed edge as $e_{i,j}^{l} \in E$ such that $e_{i,j}^{l}: N_i^{l-1} \longmapsto N_j^{l}$ i.e. an edge that connects node N_i^{l-1} at level $l-1$ to node N_j^{l} at level l. An example of the various types of directed edges formed from continuous fragments are given in Figure 2. Evidence from the reads indicates the heterozygous alleles assigned at level $l-2$ (C, T) are unambiguously in phase with the alleles assigned at level $l-1$, (G, A). As a consequence, only directed edges $e_{1,1}^{l-1}: N_1^{l-2} \longmapsto N_1^{l-1}$ and $e_{2,2}^{l-1}: N_2^{l-2} \longmapsto N_2^{l-1}$ are required to connect alleles C and T to alleles G and A respectively. At consecutive levels $(l-1,l)$ read evidence suggests there is ambiguity in the phase of the alleles (A, T) assigned at level l. For this reason, directed edges, $e_{1,1}^{l}, e_{2,2}^{l}$ are formed to ensure the optimisation algorithm understands there is potential connectedness across the levels for alleles (A, T) in the correct phase. As there is phase ambiguity, and therefore potential for the allelic phase to be switched, an additional edge $e_{2,1}^{l}: N_2^{l-1} \longmapsto N_1^{l}$ is included to connect allele A at level $l-1$ to allele A at level l.

Figure 2. *H-DAG with generated directed edges based on evidence from continuous DNA fragments (red, green and yellow paired-end reads) that span consecutive levels. Purple paired-end reads are considered discontinuous as the span non-consecutive levels.*

Induced directed edges for discontinuous fragments

After generating all the directed edges based on evidence from the continuous DNA fragments, the H-DAG will most likely contain discontinuities or no directed edges between some adjacent levels. When paired end read evidence indicates there are discontinuous DNA fragments spanning these levels (see Figure 2), the HaploMiner algorithm then attempts to build new *induced directed edges* to connect the source node containing the heterozygous allele in the left paired end read to the target node containing the heterozygous allele in the right paired end read. We use a recursive depth-first pre-order traversal method [19, 20] where, specifically for the H-DAG, we define a simple algorithm for inducing the edges between adjacent levels based on read evidence and pre-existing knowledge of constructed directed edges in previous levels of the H-DAG. For a given discontinuous DNA fragment, suppose read evidence initiates this traversal from source node N_i^l with source node sibling defined as N_j^l where $i, j \in$ 1,2 $j \neq i$. The potential children of these nodes are N_1^{l+1} and N_2^{l+1} at the $l+1$ level of the H-DAG. The algorithm is then defined by the pseudo code

getPotentialChildren from parent source node N_i^l with sibling node N_j^l at level l

  if N_j^l has an observed allele AND also has directed edges to both nodes at level $l+1$

    generate directed edges from source node N_i^l to both nodes at level $l+1$

  else if N_j^l does not have an observed allele OR has zero or one edge to nodes at level $l+1$

    for any node at level $l+1$ that has no parent AND is not the sibling node of the target node

      generate a directed edge from the source node N_i^l to that node

Various examples of how this algorithm induces new directed edges are presented in Figure 3. In Figure 3 (A), a previously known directed edge exists between nodes containing alleles T and C at the l and $l+1$ levels of the graph respectively, suggesting there is unambiguous phase of the allelic pairs

between these levels. Using the *getPotentialChildren* algorithm at level l, we induce a directed edge from the source node with allele A at level l to the node with allele T at $l+1$ level to match the unambiguous phase. At the $l+1$ level, the depth first traversal algorithm then assumes allele T has become the new parent node and a single induced directed edge is generated from T to the target node containing the allele G to align with the read evidence from the discontinuous fragment. In Figure 3 (B) the pre-existing directed edges indicate there is phase ambiguity of the allelic pairs between levels l and $l+1$. This forces *getPotentialChildren* to induce directed edges from the source node containing allele A to both nodes at level $l+1$. The recursive traversal algorithm then sequentially assumes each node at level $l+1$ is a parent node and generates a directed edge from each of the nodes to the target node. Figure 3 (C) follows identically to Figure 3 (B) due to the phase ambiguity of the allelic pairs between levels l and $l+1$. Figure 3 (D) then follows identically from Figure 3 (A).

The pre-order depth traversal algorithm, along with the *getPotentialChildren* method, is then repeated for each discontinuous DNA fragment along the H-DAG. Once complete, the H-DAG has obtained maximum connectivity between levels based on the complete DNA evidence from continuous and discontinuous fragments. Within this final H-DAG, disconnected levels may still exist where there is lack of read evidence to generate directed edges. As a consequence the H-DAG may be partitioned

Figure 3: *Various H-DAG possibilities with induced directed edges linking the discontinuous purple DNA fragment from the source node containing allele A at level* l *to target node containing allele G at level* $l+2$. *Black edges are pre-existing directed edges; blue edges are new induced directed edges using the getPotentialChildren algorithm.*

into several sub-graphs based on distinct genomic *blocks* where each block contains consecutive levels of the original H-DAG with at least one directed edge between each level. The remaining algorithmic sections will discuss an approach for the numerical estimation of edge weights in a general H-DAG block as well as the path traversal optimisation technique to appropriately phase the heterozygous alleles within each block.

Estimating directed edge weights

Within each H-DAG block, the HaploMiner algorithm assigns each directed edge an edge counter, initialized at one. The edge counters are then incremented by one every time a directed edge is spanned by a DNA fragment. Estimation of directed edge weights then uses the local probability of a directed edge defined as the ratio of its counts to the total counts of edges emitting from the source node. This calculation varies depending on the number of directed edges that were generated from the source node as well as the number of DNA fragments spanning the edges. As an example, consider the $(l-2, l-1, l)$ levels of the H-DAG in Figure 2. In the simplest case where read evidence indicates adjacent allelic pairs have unambiguous phase, such as the allelic pairs in levels $l-2$ and $l-1$ of the graph, each of the nodes in level $l-2$ are source nodes and emit one directed edge each to their target node. In this case, the edge weights can be immediately estimated as $w_{1,1}^{l-1} = pr(e_{1,1}^{l-1}) = pr(e_{2,2}^{l-1}) = w_{2,2}^{l-1} = 1$. For phase ambiguous allelic pairs at levels $l-1$ and l of the H-DAG, the source node N_2^{l-1} containing allele A emits two directed edges, $e_{2,1}^{l}$and $e_{2,2}^{l}$. Let $n(e_{2,1}^{l})$ and $n(e_{2,2}^{l})$ indicate their associated edge counts based on the cumulative read evidence for the existence of each directed edge. Then the edge weights are then estimated by the local probability

$$w_{2,1}^{l} = pr(e_{2,1}^{l}) = \frac{n(e_{2,1}^{l})}{n(e_{2,1}^{l}) + n(e_{2,2}^{l})} \;, \qquad w_{2,2}^{l} = pr(e_{2,2}^{l}) = \frac{n(e_{2,2}^{l})}{n(e_{2,1}^{l}) + n(e_{2,2}^{l})} \qquad (1)$$

and have the property, $w_{2,1}^{l} + w_{2,2}^{l} = 1$. This simple probabilistic approach for estimating directed edge weights from source nodes is then replicated sequentially throughout the H-DAG blocks using the continuous and discontinuous DNA fragment evidence spanning each of the edges.

Minimum weighted path

Without loss of generality, let any H-DAG block be defined by the sub-graph $\mathcal{G}_b = (N_b, E_b, L_b)$ and let $P_b = \{P_1, P_2, \ldots, P_t\}$ be the complete set of distinct paths through the block. For any path $P_k \in P_b$, a unique set of nodes $N_{b:k} \in N_b$, such that $N_{b:k} = \{Start_k, N_k^1, N_k^2, \ldots, N_k^{L_b}, End_k\}$, are visited across the L_b levels where, at any level of the sub-graph, l_b say, $N_k^{l_b}$ is *one* of the nodes from the pair

$(N_1^{l_b}, N_2^{l_b})$. Similar to the complete graph, for the purpose of optimisation, the sub-graph is also capped at each end with dummy nodes $(Start_k, End_k)$ nodes. As the path P_k traverses across a unique set of nodes, it also comprises of a unique set of directed edges defined by $E_{b:k} \in E_b$ where $E_{b:k} = \{Start_k \longmapsto N_k^1, N_k^1 \longmapsto N_k^2, \dots, N_k^{L_b-1} \longmapsto N_k^{L_b}, N_k^{L_b} \longmapsto End_k\} = \{Start_k \longmapsto N_k^1, e_k^1, \dots, e_k^{L_b-1}, N_k^{L_b} \longmapsto End_k\}$. We define the likelihood of this path as

$$Q_k = pr(P_k) = \prod_{l_b=1}^{L_b-1} pr\left(N_k^{l_b} \longmapsto N_k^{l_b+1}\right) = \prod_{l_b=1}^{L_b-1} w_k^{l_b} \qquad (2)$$

where $w_k^{l_b} = pr(e_k^{l_b})$, the local probability or weight of the directed edge that traverses from level $l_b - 1$ to level l_b of the H-DAG block. Determining the appropriate phase of the haplotype within the H-DAG block is then equivalent to finding the path with maximum likelihood over the complete set of paths.

For the purpose of utilizing a known path traversal algorithm we can equivalently frame this optimization as a minimization problem. Let $S_b = \{S_1, S_2, \dots, S_t\}$ be a set of values for the complete set of path traversals through the H-DAG block such that $S_k = -\log Q_k$. Determining the optimal path through the H-DAG block is then equivalent to finding the minimum negative log-likelihood path over the complete set of paths, namely

$$\min_{S_k \in S_b} \{ S_k, k = 1, \dots, t; S_k = \sum_{l_b=1}^{L_b-1} v_k^{l_b} \} \qquad (3)$$

where $v_k^{l_b} = -\log\left(w_k^{l_b}\right) \geq 0 \; \forall \; k, l_b$ define the directed edge weights used in the optimisation algorithm. Optimisation of (3) can then equivalently be viewed as finding the path of minimum weight through the H-DAG block. As the H-DAG block has no directed cycles it can be immediately topologically sorted and we can then use an established backtracking algorithm to compute the minimum weighted path [14]. This backtracking algorithm is also known to be efficient, requiring $O(n(E_b) + n(N_b))$ linear time, where $n(E_b)$ and $n(N_b)$ are the number of directed edges and nodes in the H-DAG block.

After the backtracking algorithm completes, the first haplotype is obtained by traversing through the H-DAG block on the minimum weighted path and selecting one node at each level on the path. The corresponding second haplotype is then obtained by traversing the minimum weighted path and selecting the alternate allele (the allele not on the path) at each level of the H-DAG. The backtracking algorithmic process is then repeated for each H-DAG block. The algorithm halts once the minimum weighted path is obtained for the final H-DAG block containing the right hand dummy node for the chromosome. Figure 4 presents a flow chart of the complete HaploMiner algorithm for each chromosome from the initial variant calling through to the repeated construction of two sequences for each haplotype block.

Figure 4. *Workflow schematic of the HaploMiner algorithm.*

Results

Individual NA12877 paired-end reads

The human reference genome version 38 was used to map 162.6 million paired-end reads from NA12877 individuals with 83.6% of reads aligned concordantly. The average mismatch rate was estimated 0.24% and which was similar to previous reports of heterozygosity levels in humans [21]. A total of 1.86% of reads contained small INDELs up to 20bp. The median insert size (DNA fragment length) was estimated to be 316bp. The sorted BAM file along with the NA12877 phased VCF file was then given as input to HaploMiner and the comparative haplotype construction software, HapCompass, HapCUT2 and WhatsHap (see Supplemental Material for execution commands). All algorithms were computationally conducted using a cloud-based Linux instance with 2 cores and 8GB RAM. HaploMiner, HapCompass and HapCUT2 completed in approximately 10 minutes and WhatsHap required approximately 40 minutes to complete.

Table 1. *Comparison of various statistics obtained from the output of the four haplotype phasing algorithms applied to individual NA12877 short paired-end reads.*

	INDEL support (paired-end reads)	Switch Error Rate (per Mbp)	N50(bp)	Average Haplotype Length(bp)	Number of Haplotype Blocks	Maximum Haplotype Length (bp)
HaploMiner	Yes	32.1	326	251	322,480	2770
HapCompass	Yes	143.9	329	246	339,425	2770
HapCUT2	No	37.6	313	225	285,017	2581
WhatsHap	Yes	84.2	329	252	324,362	2770

An R script, available at https://github.com/mfruzan/HaploMiner/comparison.R, was used to process the output of the four haplotype algorithms. The switch error rate, the N50 of haplotype blocks and other useful statistics were extracted and presented in Table 1. The table demonstrates HaploMiner had the lowest switch error rate compared to the other algorithms. HaploMiner, HapCompass and Hapcut2 comparably generated a similar number of haplotype blocks, 322K and 339K and 324K respectively, and all three algorithms achieved equivalent maximum haplotype lengths of 2770bp. Table 1 also indicates HapCompass and WhatsHap had substantially higher switch error rates compared to HaploMiner and HapCUT2. Although HapCUT2 had a competitively low switch error rate, it also had shorter and fewer haplotype blocks. This was most likely due to the inability of the algorithm to detect INDELs in paired-end reads.

To more rigorously assess the changes in the switch error rate as the haplotype block size increased, Figure 5 presents the switch error rate of each of the algorithms against the haplotype length (in number of base pairs). Figure 5 revealed, as haplotype blocks became longer, HaploMiner maintained a competitively low switch error rate compared to the other three algorithms indicating the HaploMiner algorithm preserves accuracy as the size of haplotypes increases. The switch error rate obtained from the HapCUT2 algorithm remained competitive against HaploMiner for shorter haplotype block lengths. However, when the haplotype block length increased to 1500 bp the switch error rate of HapCUT2 became higher than HaploMiner. WhatsHap and HapCompass were the least competitive across all haplotype block size lengths.

Figure 5. *Comparison graph showing the switch error rate for different haplotype length groups across all four haplotype phasing algorithms applied to individual NA12877 short paired-end reads.*

Individual NA12878 PacBio reads

The total number of PacBio reads was 40 million and their median and mean length was 3611 bp and 5005 bp respectively. Almost 92% of reads contained INDELs and their size varied from 1 up to 10000 bp. The original alignment to the hg19 human genome reference was performed by BLASR[22]. Due to the lack of long read PacBio sequence specific settings in HapCompass, we only compared HaploMiner with Hapcut2 and WhatsHap,. We used a cloud-based instance of 8 cores and 32GB of RAM. The computational implementation of the HaploMiner algorithm executed 1.8 times faster than HapCut2 and 2.5 times faster than WhatsHap.

An R script, available at https://github.com/mfruzan/HaploMiner/comparison2.R and was used to process the output of the three haplotype phasing algorithms. Table 2 contains the switch error rate, the N50 of haplotype blocks and other useful extracted statistics. The table indicates HaploMiner was superior in generating much longer haplotypes blocks while maintaining a competitively low switch error rate. In particular, the N50 and average haplotype length obtained from the HaploMiner algorithm was four to five times longer than the competing algorithms. HapCut2 was associated with lowest switch error rate, but similar to the paired-end reads, it ignored INDELs and also generated shortest haplotype blocks. The increased haplotype lengths obtained from HaploMiner also ensured a greater coverage of the total human reference genome (3 Gbp).

Table 2. *Comparison of various statistics obtained from the output of the three haplotype phasing algorithms applied to individual NA12878 PacBio reads*.

	INDEL support (Pacbio reads)	Switch Error Rate (per Mbp)	N50(bp)	Average Haplotype Length(bp)	Total Genome Coverage(Gbp)	Maximum Haplotype Length (bp)	Running Time (minutes)

HaploMiner	Yes	27.4	46,787	25,490	1.73	351,891	208
HapCUT2	No	5.8	10,804	4,802	1.42	118,473	380
WhatsHap	Yes	20.7	14,828	4,737	1.23	265,020	518

Figure 6 presents the switch error rate of each of the algorithms against the haplotype length. In this figure we only compared blocks up to 250 Kbp as HaploMiner was the only algorithm to be able to generate longer blocks greater than 250 Kbp. The figure indicates, even for shorter haplotype block lengths, HaploMiner maintained a competitively low switch error rate.

Figure 6. *Comparison graph showing the switch error rate at different haplotype length groups for three haplotype phasing algorithms applied to individual NA12878 PacBio reads*.

Discussion

In this research we presented an improved reference sequence-based haplotype construction algorithm, HaploMiner, that accurately assembled haplotypes of diploid genomic sequences by framing the problem as a weighted DAG and phasing haplotypes using optimal path traversal algorithmic techniques. The novel strategy of inducing new directed edges based on read evidence to resolve the disconnected levels of the H-DAG enabled HaploMiner to more accurately phase longer genomic regions compared to other leading reference-based sequence algorithms, HapCUT2, HapCompass and WhatsHap. From a computational standpoint, the HaploMiner algorithm was shown to scale well with a significant reduction in computing time when longer more computationally intensive PacBio reads were used. The significant increase in haplotype block lengths also ensured greater coverage of the genome.

It is important to note the phasing of INDEL polymorphisms has been incorporated into the HaploMiner algorithm and it managed to maintain a competitive accuracy. This is a crucial aspect of the algorithm as INDELs were the major source of switch errors in HapCUT2 and other competitive haplotype

construction software [12, 13]. While omitting INDELs can result in achieving more accurate results, it is also disadvantageous as it eliminates the second most numerous source of allelic variation in the human genome [23] and many INDELs are also known to be linked to many traits and diseases [24].

The length of assembled haplotypes depends on level of genome heterozygosity, DNA read length and coverage of sequencing used. The human genome has significantly lower level of heterozygosity compared to other diploid organisms such as *Arabidopsis thaliana* [25]. Therefore we can expect shorter assembled haplotypes from human genomic sequences. In our example, using DNA fragments (insert size) of 316bp and a paired-end sequencing coverage of 15X, the HaploMiner algorithm achieved a maximum of 2770 bp of assembled haplotype length. While this is short relative to the total length of a human chromosome, it is sufficient to enable PCR primer and CRISPR/Cas9 guide RNA based experiments where a short homologue specific sequence around a genomic position is required [26], [27]. In contrast, when using PacBio read lengths of 5 Kbp average, the HaploMiner algorithm managed to assemble haplotypes up to 350 Kbp. This result emphasizes the importance of sequencing longer reads if greater haplotype length is required.

Although we demonstrated the ability of the HaploMiner algorithm to accurately assemble human diploid genomic sequences, its potential is now being explored for other areas of related genomic sequence research. For example, the algorithm could be used to assemble haplotypes for genomes from tetraploid and hexaploid species, such as wheat, as long as there are separate reference sequences for each of the homoeologous copies of the chromosomes. In cases where a set of genomic sequences have been generated from a population of related individuals, we are exploring the use of the base algorithm of HaploMiner for discovering the most recurring haplotypes among the population.

Conclusion

By framing the haplotype assembly problem as a DAG and using a novel edge inducing strategy for discontinuous DNA fragments, the HaploMiner algorithm was able to accurately phase long haplotype blocks using short or long sequence reads. The algorithm was shown to be highly efficient and also has potential to be impactful in similar genomic sequence research areas where accurate haplotype phasing

or selection is required. To ensure the portability of the HaploMiner algorithm across varying computing architectures it has been implemented in Java and is available under MIT license from https://github.com/mfruzan/HaploMiner.

Availability of Supporting Data and Materials

The two FastQ files and the sorted BAM file relating to the NA12877 individuals are accessible through NCBI SRA at https://www.ncbi.nlm.nih.gov/sra/PRJNA664648. The VCF files relating to the individuals NA12877 and NA12878 are publicly accessible through the Figshare repository at https://figshare.com/s/39dfcc6216de19234de0. The human genome reference and its Bowtie2 index was downloaded from the link within https://sapac.support.illumina.com/sequencing/sequencing_software/igenome.html.

The Bowtie read alignment software version 2.4.1 was downloaded from https://sourceforge.net/projects/bowtie-bio/files/bowtie2/2.4.1/. Comparative haplotype assembly software, HapCompass version 0.8.2 was downloaded from https://www.brown.edu/Research/Istrail_Lab/hapcompass.php and required Java 1.8 or higher to execute. HapCUT2 was compiled from https://github.com/vibansal/HapCUT2 and for execution required installation of high-throughput sequencing tools library htslib from https://github.com/samtools/htslib. WhatsHap version 1.0 (latest) depends on python version 3.6 or higher and C++ compiler and was installed using pip.

Availability of Source Code and Requirements

The HaploMiner source code and Java executable (MFbio.jar file) are publicly accessible from https://github.com/mfruzan/HaploMiner under MIT license. The resulting haplotype output files for all four algorithms is accessible from Figshare https://figshare.com/s/39dfcc6216de19234de0 . R code to process the output files and generate reports is located at https://github.com/mfruzan/HaploMiner in *comparison.R* and *comparison2.R.*

Abbreviations

SNP: Single Nucleotide Polymorphism; DAG: Directed acyclic graph; H-DAG: Haplotype DAG; bp: base pair; Mbp: Mega base pair; MEC: Minimum Error Correction; INDEL: Insertion Deletion; PCR: Polymerase Chain Reaction; CRISPR: Clustered Regularly Interspaced Short Palindromic Repeat; VCF: Variant Calling Format; RNA: Ribonucleic acid; PacBio: Pacific Biosiences

Competing interests

The authors declare no competing interests.

Authors Contributions

MF and JT designed the algorithm. MF, WT, OK and JT wrote the manuscript. MF wrote source code in Java. MF and JT wrote source code in R. MF performed analysis.

Acknowledgements

The authors gratefully acknowledge the Grain Research and Development Corporation (GRDC) of Australia for supporting this research. We also acknowledge the support from all the staff specially Russell Edson in the Biometry Hub the School of Agriculture, Food and Wine, University of Adelaide.

References

1. Berger E, Yorukoglu D, Peng J and Berger B. Haptree: A novel bayesian framework for single individual polyplotyping using ngs data. PLoS computational biology. 2014;10 3:e1003502.
2. Excoffier L and Slatkin M. Maximum-likelihood estimation of molecular haplotype frequencies in a diploid population. Molecular biology and evolution. 1995;12 5:921-7.
3. Delaneau O, Coulonges C and Zagury J-F. Shape-IT: new rapid and accurate algorithm for haplotype inference. BMC bioinformatics. 2008;9 1:540.
4. Kong A, Masson G, Frigge ML, Gylfason A, Zusmanovich P, Thorleifsson G, et al. Detection of sharing by descent, long-range phasing and haplotype imputation. Nature genetics. 2008;40 9:1068.
5. Browning SR and Browning BL. Rapid and accurate haplotype phasing and missing-data inference for whole-genome association studies by use of localized haplotype clustering. The American Journal of Human Genetics. 2007;81 5:1084-97.
6. Delaneau O, Zagury J-F and Marchini J. Improved whole-chromosome phasing for disease and population genetic studies. Nature methods. 2013;10 1:5-6.

7. Delaneau O, Zagury J-F, Robinson MR, Marchini JL and Dermitzakis ET. Accurate, scalable and integrative haplotype estimation. Nature communications. 2019;10 1:1-10.
8. Majidian S, Kahaei MH and de Ridder D. Minimum error correction-based haplotype assembly: Considerations for long read data. Plos one. 2020;15 6:e0234470.
9. Bansal V and Bafna V. HapCUT: an efficient and accurate algorithm for the haplotype assembly problem. Bioinformatics. 2008;24 16:i153-i9.
10. Edge P, Bafna V and Bansal V. HapCUT2: robust and accurate haplotype assembly for diverse sequencing technologies. Genome research. 2017;27 5:801-12.
11. Das S and Vikalo H. SDhaP: haplotype assembly for diploids and polyploids via semi-definite programming. BMC genomics. 2015;16 1:260.
12. Patterson M, Marschall T, Pisanti N, Van Iersel L, Stougie L, Klau GW, et al. WhatsHap: weighted haplotype assembly for future-generation sequencing reads. Journal of Computational Biology. 2015;22 6:498-509.
13. Aguiar D and Istrail S. HapCompass: a fast cycle basis algorithm for accurate haplotype assembly of sequence data. Journal of Computational Biology. 2012;19 6:577-90.
14. Cormen TH, Leiserson CE, Rivest RL and Stein C. Introduction to algorithms. MIT press; 2009.
15. Eberle MA, Fritzilas E, Krusche P, Källberg M, Moore BL, Bekritsky MA, et al. A reference data set of 5.4 million phased human variants validated by genetic inheritance from sequencing a three-generation 17-member pedigree. Genome research. 2017;27 1:157-64.
16. Sedlazeck FJ, Lee H, Darby CA and Schatz MC. Piercing the dark matter: bioinformatics of long-range sequencing and mapping. Nature Reviews Genetics. 2018;19 6:329-46.
17. Langmead B and Salzberg SL. Fast gapped-read alignment with Bowtie 2. Nature methods. 2012;9 4:357.
18. Zook JM, Catoe D, McDaniel J, Vang L, Spies N, Sidow A, et al. Extensive sequencing of seven human genomes to characterize benchmark reference materials. Scientific data. 2016;3 1:1-26.
19. Kural M. Tree traversal and word order. Linguistic Inquiry. 2005;36 3:367-87.
20. Morris JM. Traversing binary trees simply and cheaply. Information Processing Letters. 1979;9 5:197-200.
21. Bryc K, Patterson N and Reich D. A novel approach to estimating heterozygosity from low-coverage genome sequence. Genetics. 2013;195 2:553-61.
22. Chaisson MJ and Tesler G. Mapping single molecule sequencing reads using basic local alignment with successive refinement (BLASR): application and theory. BMC bioinformatics. 2012;13 1:1-18.
23. Mullaney JM, Mills RE, Pittard WS and Devine SE. Small insertions and deletions (INDELs) in human genomes. Human molecular genetics. 2010;19 R2:R131-R6.
24. Mills RE, Pittard WS, Mullaney JM, Farooq U, Creasy TH, Mahurkar AA, et al. Natural genetic variation caused by small insertions and deletions in the human genome. Genome research. 2011;21 6:830-9.
25. Tyagi A, Singh S, Mishra P, Singh A, Tripathi AM, Jena SN, et al. Genetic diversity and population structure of Arabidopsis thaliana along an altitudinal gradient. AoB Plants. 2016;8.
26. Lv J, Wu S, Wei R, Li Y, Jin J, Mu Y, et al. The length of guide RNA and target DNA heteroduplex effects on CRISPR/Cas9 mediated genome editing efficiency in porcine cells. Journal of veterinary science. 2019;20 3.

27. Ye J, Coulouris G, Zaretskaya I, Cutcutache I, Rozen S and Madden TL. Primer-BLAST: a tool to design target-specific primers for polymerase chain reaction. BMC bioinformatics. 2012;13 1:1-11.

Supplementary data

Supplementary_Material_1.docx

Figure1
Click here to access/download;Figure;Figure1.jpg

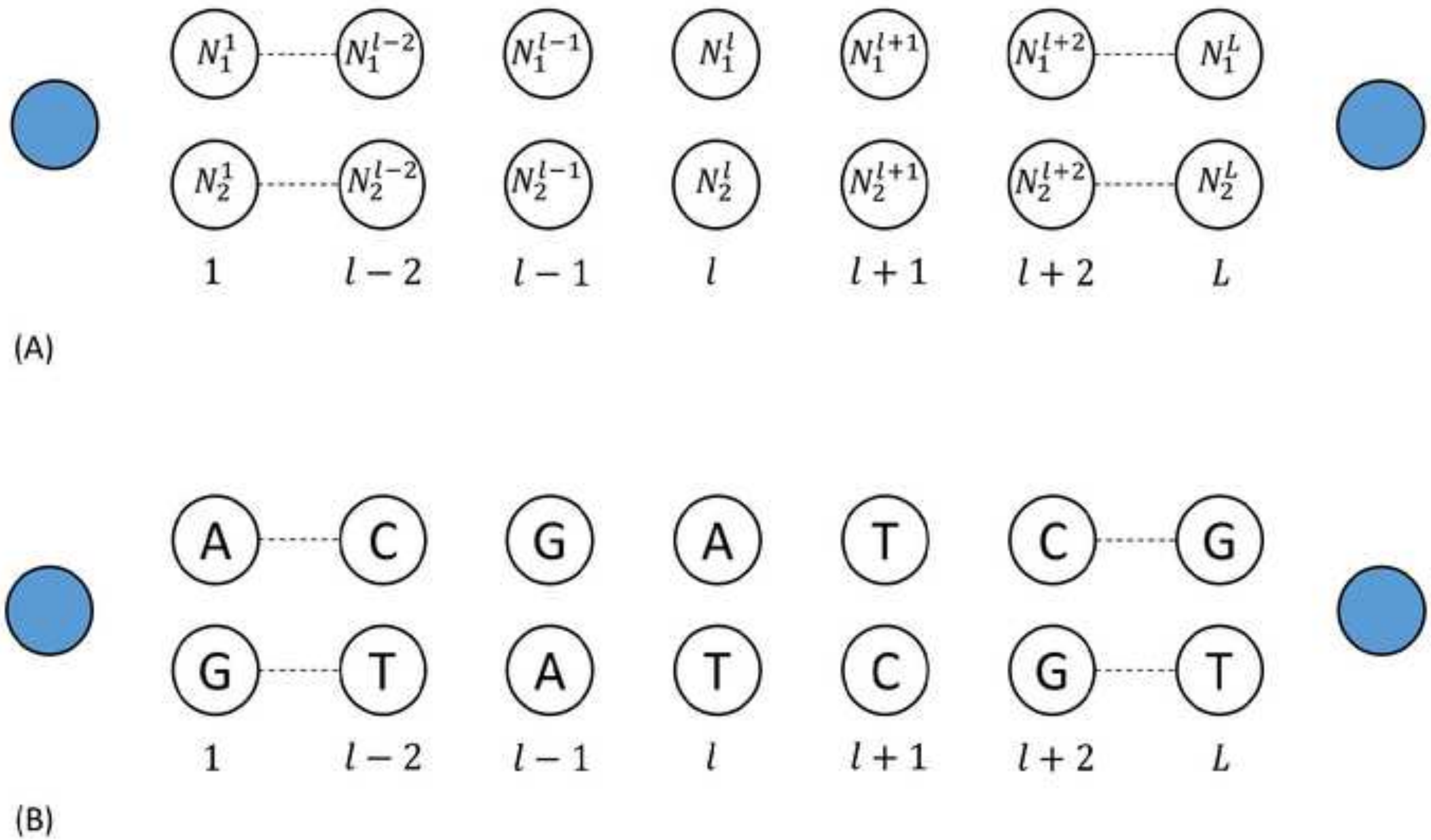

Figure2

Click here to access/download;Figure;Figure2.jpg

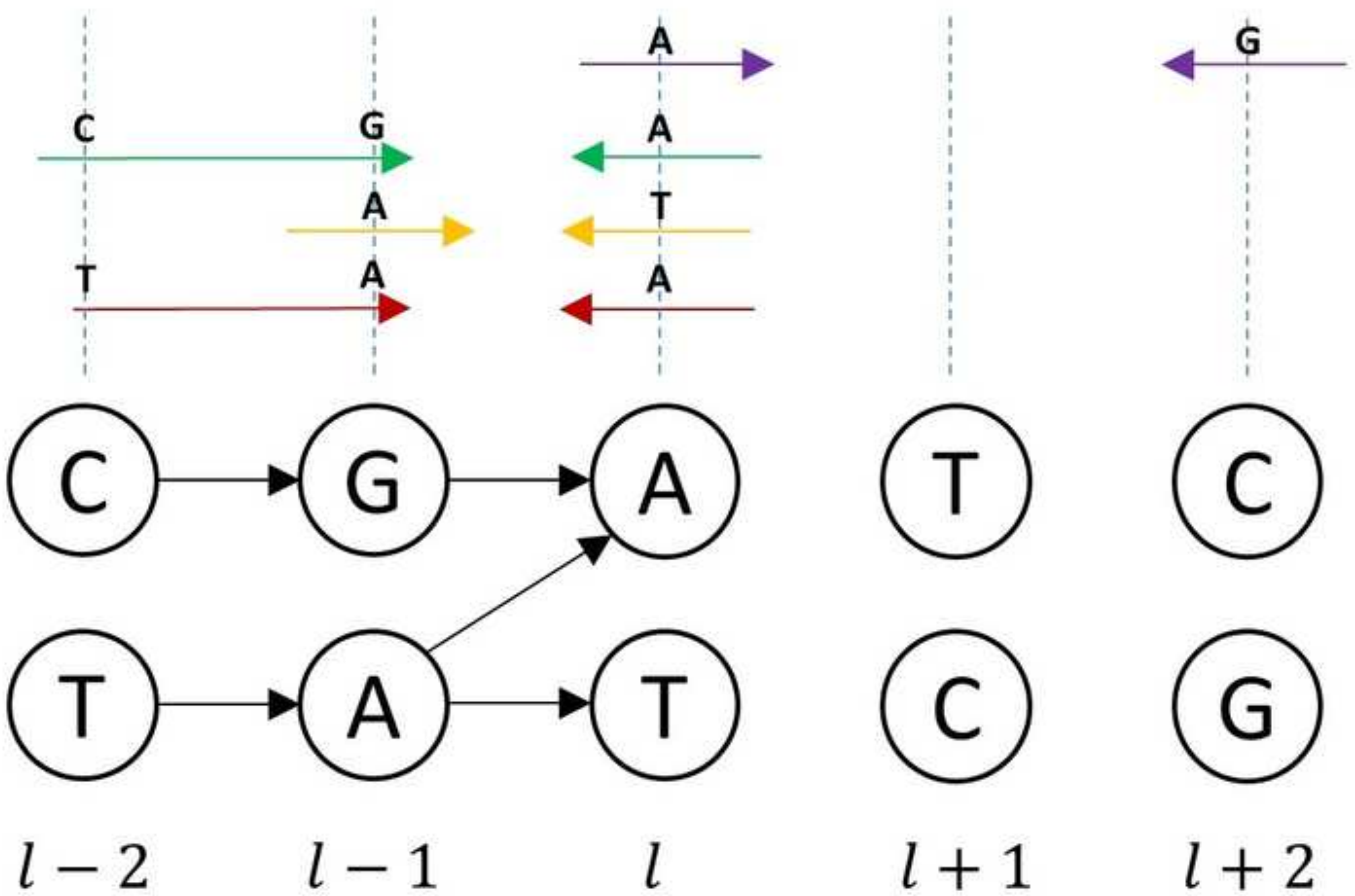

Figure3

Click here to access/download;Figure;Figure3.jpg

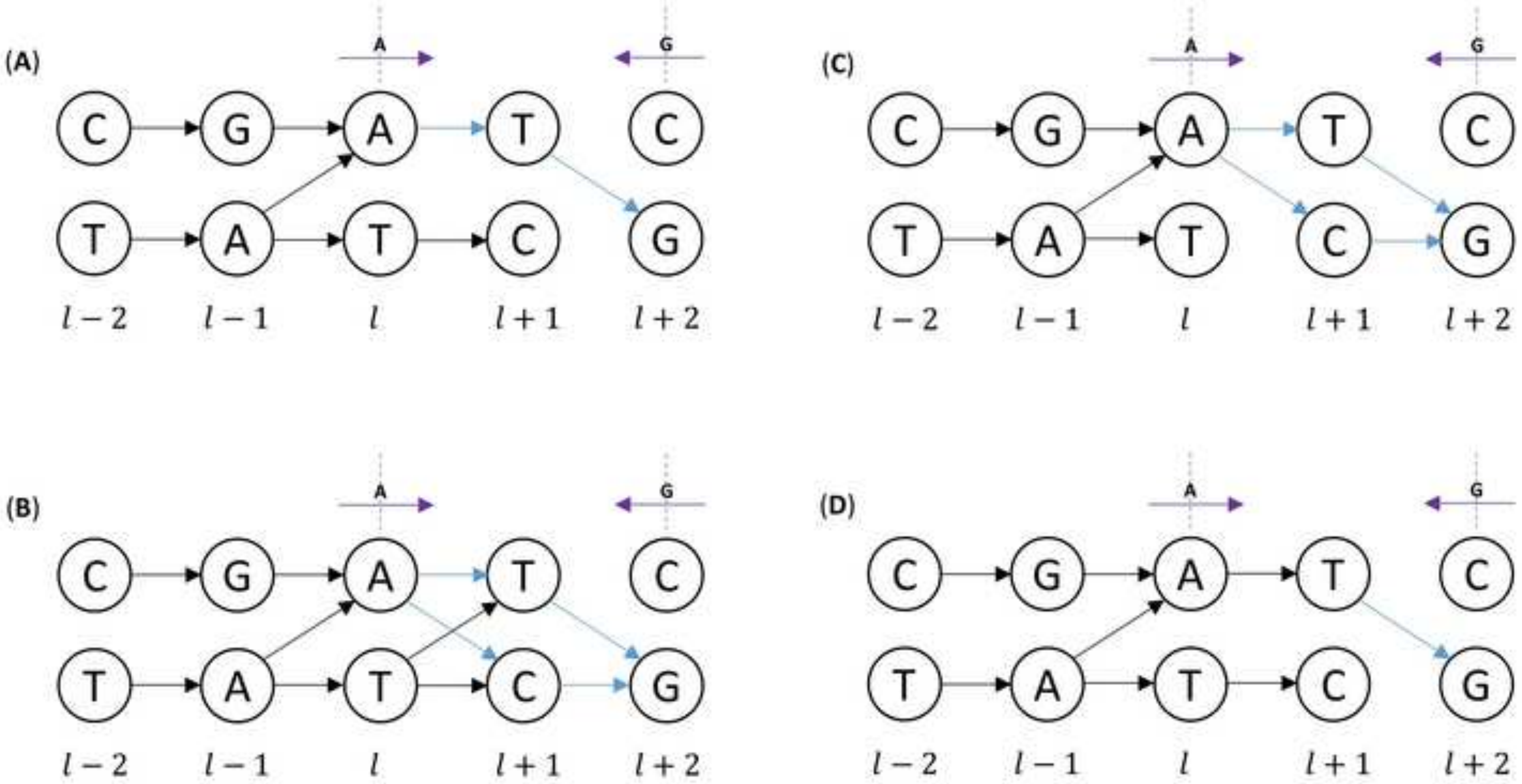

Click here to access/download;Figure;Figure4.jpg

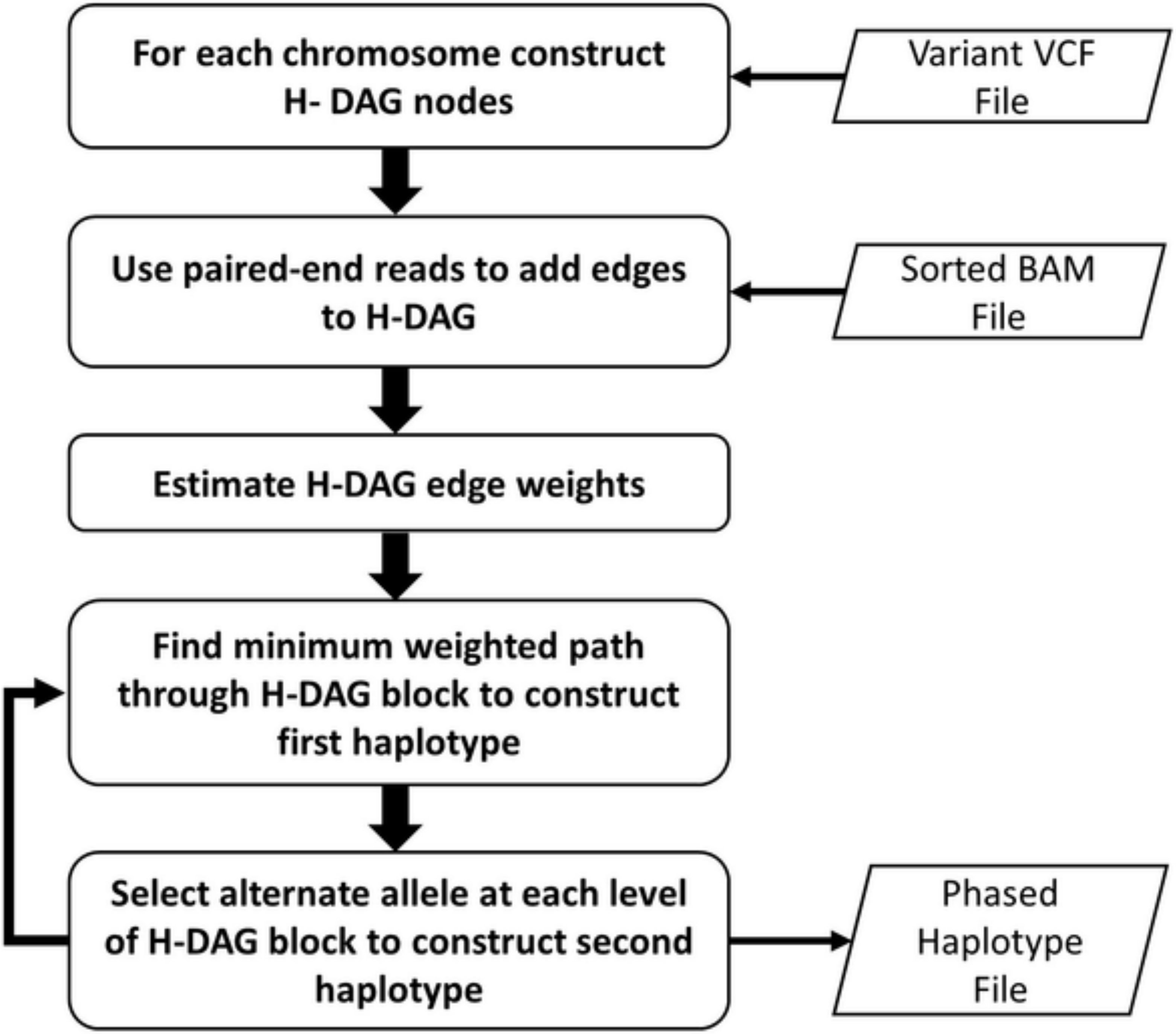

 Click here to access/download;Figure;Figure5.jpeg

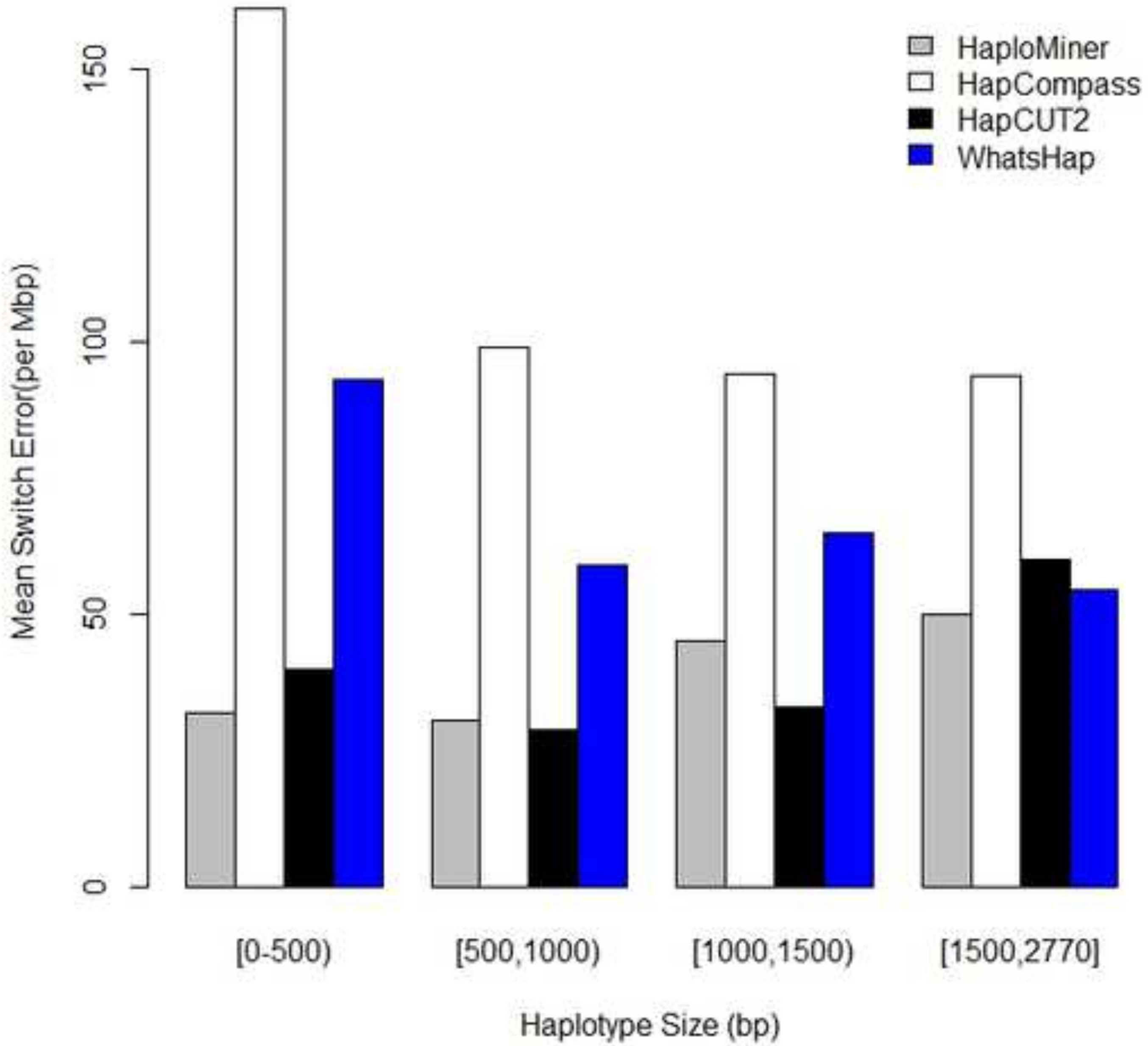

Figure6

Click here to access/download;Figure;Figure6.jpeg

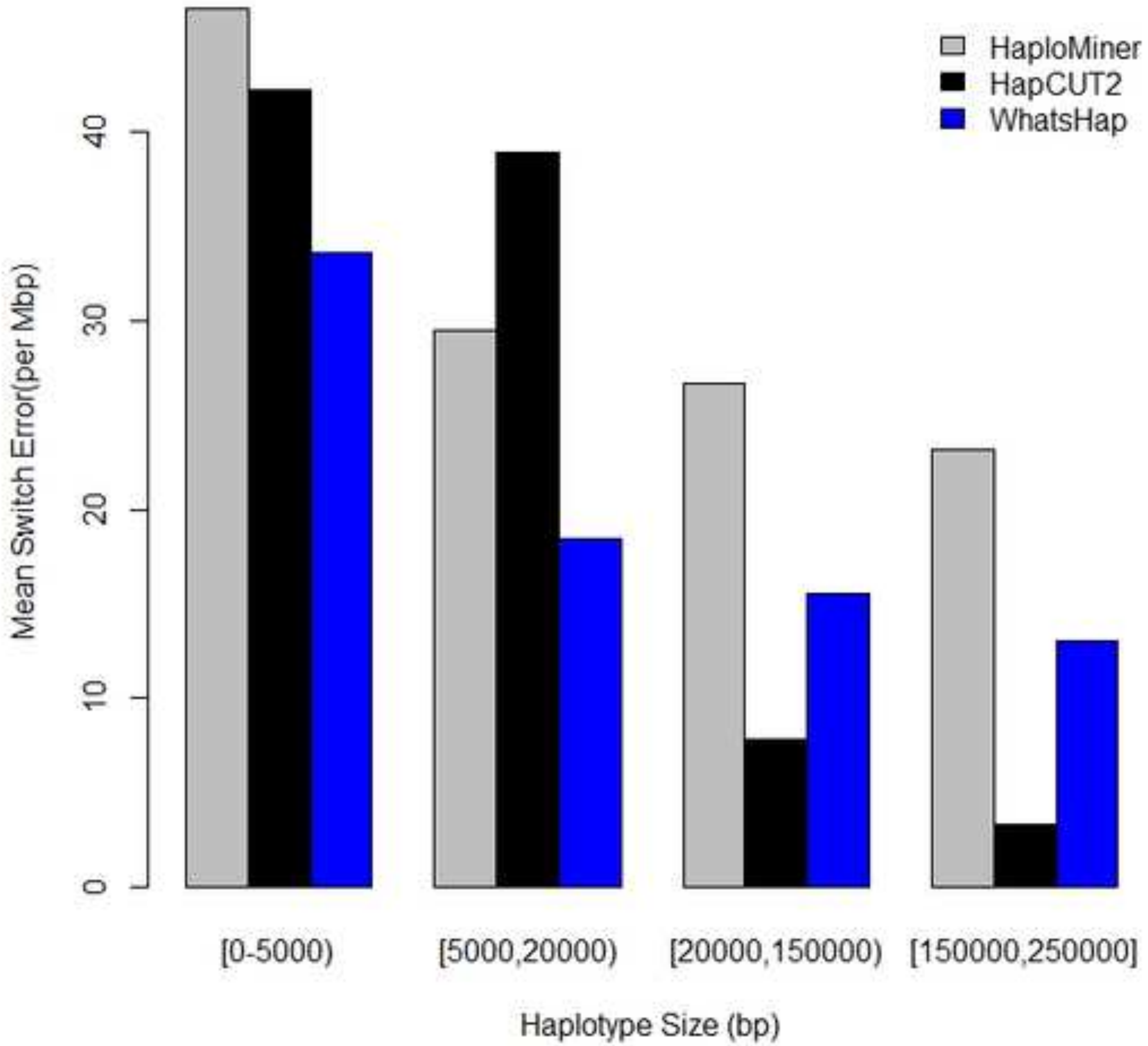

Click here to access/download
Supplementary Material
supplementary_material_1.docx
