# Supplementary material for: HaploMaker: An improved algorithm for rapid haplotype assembly of genomic sequences
Source: Gigascience. 2022 May 17;11:giac038. doi: 10.1093/gigascience/giac038 (PMC9112781; doi:10.1093/gigascience/giac038)
Supplement: giac038_GIGA-D-21-00252_Revision_3 [file giac038_giga-d-21-00252_revision_3.pdf]

GigaScience

HaploMaker: An improved algorithm for rapid haplotype assembly of genomic sequences

--Manuscript Draft--

**Manuscript Number:**	GIGA-D-21-00252R3
**Full Title:**	HaploMaker: An improved algorithm for rapid haplotype assembly of genomic sequences
**Article Type:**	Technical Note
**Funding Information:**	Grains Research and Development Corporation — Dr Olena Kravchuk
**Abstract:**	Background<br>In diploid organisms, whole genome haplotype assembly relies on the accurate identification and assignment of heterozygous single nucleotide polymorphism (SNP) alleles to the correct homologous chromosomes. This appropriate phasing of these alleles ensures combinations of SNPs on any chromosome, called haplotypes, can then be used in down-stream genetic analyses approaches including determining their potential association with important phenotypic traits. A number of statistical algorithms and complementary computational software tools have been developed for whole genome haplotype construction from genomic sequence data. However, many algorithms lack the ability to phase long haplotype blocks and simultaneously achieve a competitive accuracy.<br>Results<br>In this research we present HaploMaker , a novel reference-based haplotype assembly algorithm capable of accurately and efficiently phasing long haplotypes using paired-end short reads and longer PacBio reads from diploid genomic sequences. To achieve this we frame the problem as a directed acyclic graph with edges weighted on read evidence and use efficient path traversal and minimization techniques to optimally phase haplotypes. We compared the HaploMaker algorithm with three other common reference-based haplotype assembly tools using public haplotype data of human individuals from the Platinum Genome project. With short read sequences, the HaploMaker algorithm maintained a competitively low switch error rate across all haplotype lengths and was found to be superior in phasing longer genomic regions. For longer PacBio reads, the phasing accuracy of HaploMaker remained competitive for all block lengths and demonstrated the ability to generate substantially longer block lengths than the competing algorithms.<br>Conclusions<br>HaploMaker provides an improved haplotype assembly algorithm for diploid genomic sequences by accurately phasing longer haplotypes. The computationally efficient and portable nature of the Java implementation of the algorithm will ensure it has maximal impact in reference-sequence based haplotype assembly applications.
**Corresponding Author:**	Mario Fruzangohar<br>The University of Adelaide<br>Urrbrae, SA 5064, SA AUSTRALIA
**Corresponding Author Secondary Information:**	
**Corresponding Author's Institution:**	The University of Adelaide
**Corresponding Author's Secondary Institution:**	
**First Author:**	Mario Fruzangohar
**First Author Secondary Information:**	
**Order of Authors:**	Mario Fruzangohar<br>William A. Timmins<br>Olena Kravchuk

Powered by Editorial Manager® and ProduXion Manager® from Aries Systems Corporation

	Julian Taylor
**Order of Authors Secondary Information:**	
**Response to Reviewers:**	- Please move all URLs / dois /ftp sites /github repos referring to data sources, software etc. to the bibliography and cite them by number from the text. At GigaScience, we treat online data or software repositories and other internet resources as citable items, i.e. they should get their own numbered reference in the bibliography. Please refer to our instructions for authors for details.<br>>We have created citations for all the URL , github and software programs in the "Reference" section.<br><br>- Please also include your new GigaDB dataset in the bibliography and cite it by number from the availability section. (e.g. write "An archival copy of the code and supporting data is available via the GigaScience database, GigaDB (xx)".<br>>GigaDB citation was created and added.
**Additional Information:**	
**Question**	**Response**
Are you submitting this manuscript to a special series or article collection?	No
**Experimental design and statistics**<br><br>Full details of the experimental design and statistical methods used should be given in the Methods section, as detailed in our Minimum Standards Reporting Checklist. Information essential to interpreting the data presented should be made available in the figure legends.<br><br>Have you included all the information requested in your manuscript?	Yes
**Resources**<br><br>A description of all resources used, including antibodies, cell lines, animals and software tools, with enough information to allow them to be uniquely identified, should be included in the Methods section. Authors are strongly encouraged to cite Research Resource Identifiers (RRIDs) for antibodies, model organisms and tools, where possible.<br><br>Have you included the information requested as detailed in our Minimum Standards Reporting Checklist?	Yes

**Availability of data and materials**<br><br>All datasets and code on which the conclusions of the paper rely must be either included in your submission or deposited in publicly available repositories (where available and ethically appropriate), referencing such data using a unique identifier in the references and in the "Availability of Data and Materials" section of your manuscript.<br><br>Have you have met the above requirement as detailed in our Minimum Standards Reporting Checklist?	Yes

Powered by Editorial Manager® and ProduXion Manager® from Aries Systems Corporation

Manuscript
Click here to access/download;Manuscript;HaploMaker-for_publication.docx

HaploMaker: An improved algorithm for rapid haplotype assembly of genomic sequences

Mario Fruzangohar[1*], William A. Timmins[1], Olena Kravchuk[1], Julian Taylor[1]

[1]The Biometry Hub, School of Agriculture, Food and Wine, University of Adelaide, Australia

[*]Corresponding author

Mario Fruzangohar: mario.fruzangohar@adelaide.edu.au

William A. Timmins: andy.timmins@adelaide.edu.au

Olena Kravchuk: olena.kravchuk@adelaide.edu.au

Julian Taylor: julian.taylor@adelaide.edu.au

ORCID iDs:

Mario Fruzangohar [0000-0003-3525-8338]; William A Timmins [0000-0001-5266-056X]; Olena Kravchuk [0000-0001-5291-3600]; Julian Taylor [0000-0002-9145-052X]

Abstract

Background

In diploid organisms, whole genome haplotype assembly relies on the accurate identification and assignment of heterozygous single nucleotide polymorphism (SNP) alleles to the correct homologous chromosomes. This appropriate phasing of these alleles ensures combinations of SNPs on any chromosome, called haplotypes, can then be used in down-stream genetic analyses approaches including determining their potential association with important phenotypic traits. A number of statistical algorithms and complementary computational software tools have been developed for whole genome haplotype construction from genomic sequence data. However, many algorithms lack the ability to phase long haplotype blocks and simultaneously achieve a competitive accuracy.

Results

In this research we present *HaploMaker*, a novel reference-based haplotype assembly algorithm capable of accurately and efficiently phasing long haplotypes using paired-end short reads and longer PacBio reads from diploid genomic sequences. To achieve this we frame the problem as a directed acyclic graph with edges weighted on read evidence and use efficient path traversal and minimization techniques to optimally phase haplotypes. We compared the HaploMaker algorithm with three other common reference-based haplotype assembly tools using public haplotype data of human individuals from the Platinum Genome project. With short read sequences, the HaploMaker algorithm maintained a competitively low switch error rate across all haplotype lengths and was found to be superior in phasing longer genomic regions. For longer PacBio reads, the phasing accuracy of HaploMaker remained competitive for all block lengths and demonstrated the ability to generate substantially longer block lengths than the competing algorithms.

Conclusions

HaploMaker provides an improved haplotype assembly algorithm for diploid genomic sequences by accurately phasing longer haplotypes. The computationally efficient and portable nature of the Java

implementation of the algorithm will ensure it has maximal impact in reference-sequence based haplotype assembly applications.

Keywords: haplotype, PacBio, CCS, HiFi, CLR, Illumina, paired-end, SNP, INDEL, heterozygous, DNA sequence, genome

Supplementary information

Supplementary information is available in Supplemental Material submitted with this document.

Introduction

Diploid organisms such as human, *Arabidopsis thaliana*, barley and many other eukaryotic genomes typically contain two homologous copies of every chromosome, each inherited from either of the parents. When there is allelic variation (wild type or mutant) at one genomic position between homologous chromosomes, the position is called heterozygous. For a single heterozygous site it is possible to quantify the number of wild type and mutant alleles by using, for example, variant calling tools. However, when two or more heterozygous sites are present, it is not possible to determine whether their alleles are on the same or different chromosomes. The presence of certain alleles from multiple genomic positions on one chromosome is called a haplotype and may be associated with an important phenotypic trait in one or more individuals. In contrast, if the alleles reside on different chromosomes, this can be connected to a loss of function [1]. This distinction results in the need for computationally efficient algorithms that can accurately phase alleles on one chromosome using sequencing reads obtained from diploid organisms [2].

There are several approaches to haplotype construction [3]. One group of construction methods relies heavily on using sequence information obtained from multiple related individuals of a population. Haplotypes can then be phased and assembled using various aspects of the genomic structure of the population, such as linkage disequilibrium [4, 5]. Other construction approaches in this group have focussed on using founder sequences and inferring haplotype phase through identity by descent [6] or the use of Hidden Markov Models [7-9] Unfortunately, these approaches are generally not applicable

for haplotype phasing and assembly of sequences from a single sample when there are no additional genomic sequences available from related individuals.

This research focusses on the group of reference-based haplotype phasing algorithms used when only DNA sequences of an individual sample are available [1]. For an individual diploid organism, there should be only two haplotypes detected. However, due to sequencing errors and misalignments of reads to the reference, the total number of inferred haplotypes can potentially increase exponentially. To minimize errors in the haplotype construction, mathematical algorithms involving selection criteria are required to assist in determining the appropriate phase of the haplotypes. One strategy is to use the widely established minimum error correction (MEC) criterion [1, 10] where an optimal MEC indicates the smallest set of SNP changes that create a conflict-free separation of mapped reads into two groups. HapCUT [11], HapCUT2 [12] and SDhaP [13] belong to this category as they reconstruct a pair of haplotypes such that the fragments are maximally consistent with the assembled haplotypes. An extension of the MEC was proposed in WhatsHap [14] where a weighted MEC (wMEC) criterion was used and the optimization of haplotype construction is achieved through dynamic programming. In HapCompass [15] the problem of haplotype construction in polyploids and diploids is defined as an undirected weighted graph and an algorithm is developed that incorporates cycle basis local optimizations for resolving conflicting evidence. HapTree [2] focusses on polyploid species and develops a Bayesian maximum-likelihood framework for haplotype phasing and construction. We note here that the necessity of having a specific method for polyploid species has lessened nowadays, as newer versions of genomic references (*e.g.* tetraploid wheat) contain all homoeologous copies of one chromosome.

In this research we present a novel reference-based algorithm for phasing two haplotypes (paternal/maternal) of a single diploid organism by using its genomic sequence reads. The algorithm frames the haplotype construction problem as a directed acyclic graph (DAG) structure and determines the optimal haplotype assembly using minimal path and related graph traversal algorithms [16]. We call this algorithm *HaploMaker*. The algorithm attempts to phase longer DNA strands as long as any heterozygous position within a strand, and at least one other nearby heterozygous position, is covered

by a single DNA fragment. The algorithm provides support for phasing SNPs as well as potentially important Insertion/Deletion (INDELs) polymorphisms. Computationally, the HaploMaker algorithm has reduced execution times and only requires a minimal amount of memory.

For demonstrating and benchmarking the HaploMaker algorithm, we have chosen the human pedigree of 17 individuals (two parents, 11 children and four grandparents) from three generations [17], where their DNA has been sequenced and haplotypes of two parents have been verified using inheritance constraints in the pedigree and the concordance of variant calls across different methods. We compared HaploMaker results with those generated by HapCUT2, HapCompass and WhatsHap. WhatsHap was selected due to its widespread use in assembling haplotypes [18] and HapCUT2 and HapCompass were selected due to recent reports of their ability to generate accurate haplotype assemblies [15]. We showed that when using paired-end short reads with 10x or 25x coverage, compared to the competitors used in this research, the HaploMaker algorithm was capable of constructing longer haplotype blocks while maintaining a competitively low switch error rate. Additionally, when using longer PacBio continuous long reads (CLR) or more accurate PacBio HiFi reads, HaploMaker generated substantially longer haplotype blocks, ensuring a more complete assembled genome.

Methods

Data preparation

Individual NA12877 paired-end reads

The paired-end FastQ files for NA12877 of the Platinum Genome project were downloaded from [19] and the NA12877 phased VCF file was downloaded from [20]. The FastQ files were then sampled randomly to 10x and 25x genome coverages and the resulting 162.6 and 407.5 million paired reads from the FastQ files were mapped to the human genome reference version 38 using Bowtie 2 [21], allowing for a 1% mismatch rate and capturing short INDELs up to 20 bases (see Supplemental Material).

Individual NA12878 PacBio CLR/subreads

The sorted bam file of PacBio reads was downloaded from [22, 23]. Similar to individual NA12877, the corresponding VCF file was downloaded from the Platinum Genome project. For mapping of the PacBio reads we used the human genome reference hg19 downloaded from [24].

Individual NA12878 PacBio HiFi reads

High quality PacBio reads (known as CCS or HiFi) related to individual NA12878 were downloaded from NCBI SRA archive (accession number SRX5780566)[3]. For mapping of the reads we used the human genome reference hg19 from the PacBio subread experiment. The corresponding VCF file from the PacBio subread experiment was also used.

Haplotype directed acyclic graph (H-DAG)

To simplify the notation and development of the theoretical framework, we initially focus on a single chromosome with the understanding that the framework will apply identically to other chromosomes. From the VCF file, assume L heterozygous alleles or variants have been detected at various positions on the chromosome, with each allele pair assigned a random phase. We now formalize a framework for accurately phasing of these alleles using a directed acyclic graph and the aligned read sequence evidence.

Let $\mathcal{G}$ define a haplotype directed acyclic graph (H-DAG), such that $\mathcal{G} = (V, E)$ where

i. $V = \{(v_1^1, v_2^1), \dots, (v_1^l, v_2^l), \dots (v_1^L, v_2^L)\}$ defines the complete set of nodes (or vertices); (v_1^l, v_2^l) are considered a sibling pair of nodes at the lth level of the graph.

ii. $E = \left\{\left(v^\delta, v^{\delta+1}\right) \mid v^\delta \in v_i^\delta, v^{\delta+1} \in v_j^{\delta+1}, i, j = 1,2, \forall\, \delta = 1, \dots, L-1\right\}$ defines the set of all possible edges between nodes in L nodes in V.

The sibling paired node structure for a H-DAG with L levels is visually represented in Figure 1A with the complete skeleton H-DAG containing two dummy nodes at the beginning and end of the graph. Using this node structure, and without additional read sequence evidence, the L ordered pairs of heterozygous alleles are sequentially assigned to pairs of nodes.. This assignment is represented in

Figure 1B where, for example at the lth level, the pair of nodes have been assigned alleles $v_1^l = \mathrm{A}$ and $v_2^l = \mathrm{T}$ in the skeleton H-DAG.

The definition of the H-DAG edges indicates directed edges can only exist between adjacent levels of the graph ensuring no directed cycles are possible. Additionally, for any directed edge, $(v^{\delta}, v^{\delta+1}) \in E$, between arbitrary adjacent levels, v^{δ} is explicitly the parent node of $v^{\delta+1}$ and $v^{\delta+1}$ is explicitly the child node of v^{δ}. This implies directed edges can only occur left to right (low to high levels) sequentially across the H-DAG. Without additional evidence from the sequencing reads, the ambiguity of allelic phase at any level of the graph suggests all directed edges are possible. In the following sections we discuss using evidence from the DNA reads to refine the rules of generating directed edges across the adjacent levels of the H-DAG.

Figure 1. *(A) Skeleton of the H-DAG with L pairs of nodes and two dummy nodes capping each end of the graph. (B) Skeleton of the H-DAG showing the random assignment of heterozygous pairs of alleles to each pair of nodes.*

Continuous and discontinuous DNA fragments

Once the heterozygous alleles are assigned to the skeleton H-DAG, the HaploMaker algorithm is ready to generate directed edges sequentially across the levels of the graph using evidence from the DNA reads. To illustrate the development of this component of the algorithm we have focussed on paired end reads but a similar argument applies for longer single end reads. During the processing of the read evidence, a DNA fragment (matching left and right paired-end reads) was considered to be continuous if it spanned consecutive levels of the H-DAG without loss of coverage across any of the heterozygous alleles contained in those levels. This is exemplified in Figure 2 where the $(l-4, \ldots, l+2)$ levels of the example skeleton H-DAG from Figure 1 are used. The paired-end reads (arrows above nodes) are considered continuous DNA fragments as they span at least two consecutive levels $(l-4, \ldots, l)$ of the H-DAG with read evidence indicating they also contain the heterozygous alleles in those levels.

In contrast, a DNA fragment was considered to be discontinuous if it spanned non-consecutive levels of the H-DAG. Discontinuities such as this are common and can arise when the reference DNA fragment insert size exceeds the aggregate size of the left and right paired-end read lengths. Figure 2 provides a simplified example of this where the purple end reads are considered discontinuous as they span levels $(l, l+1, l+2)$ with a discontinuity, indicating a lack of coverage across the $(l+1)th$ level.

Directed edges for continuous DNA fragments

Initially, the HaploMaker algorithm builds directed edges sequentially across levels of the H-DAG spanned by the continuous fragments. Due to the initial random assignation of allelic phase to any pair of nodes in the skeleton H-DAG, and the potential of DNA misalignment or sequencing errors, the number of directed edges constructed between any two consecutive levels of the H-DAG can vary. Consider the node framework for arbitrary adjacent levels δ and $\delta+1$ of the H-DAG and let the set of edges between these levels be defined by E^{δ}. Based on continuous DNA fragment evidence, four distinct scenarios are possible for the consecutive allele pairs:

1. Unambiguous in phase: $E^{\delta} = \left\{\left(v^{\delta}, v^{\delta+1}\right) \mid v^{\delta} \in v_i^{\delta}, v^{\delta+1} \in v_i^{\delta+1}, i = 1{,}2\right\}$. This indicates there is read evidence that the allele pairs are in phase with no ambiguity and this supports the generation of two straight directed edges (not crossing over) from the parent nodes to child nodes.

2. Unambiguous out of phase: $E^{\delta} = \left\{\left(v^{\delta}, v^{\delta+1}\right) \mid v^{\delta} \in v_j^{\delta}, v^{\delta+1} \in v_i^{\delta+1},\ i,j = 1{,}2;\ j \neq i\right\}$. This indicates there is read evidence that the allele pairs are out of phase with no ambiguity and this supports the generation of two directed edges that cross over from the parent nodes to the child nodes.

3. Ambiguous phase of one allele pair: $E^{\delta} = \left\{\left(v^{\delta}, v^{\delta+1}\right) \mid v^{\delta} \in v_i^{\delta}, v^{\delta+1} \in v_j^{\delta+1},\ i = 1, j = 1{,}2; i = j = 2\right\}$. This indicates there is read evidence suggesting phase ambiguity of the allele pair at level δ of the graph. This supports the generation of two directed edges using **1.** and a third diagonal directed edge from one parent node to a child node matching the read evidence.

4. Ambiguous phase of both allele pairs: $E^{\delta} = \{(v^{\delta}, v^{\delta+1}) \mid v^{\delta} \in v_i^{\delta}, v^{\delta+1} \in v_j^{\delta+1}, i, j = 1,2\}$, indicating there is read evidence that suggests phase ambiguity of both allele pairs and this supports the generation of four directed edges using **1.** and **2.** defined above to match the conflicting read evidence.

Figure 2 provides a visual representation of the directed edge types spanning the $(l-4, \ldots, l)$ consecutive levels of an example H-DAG with the supporting read evidence above the graph nodes. It should be noted that the mirror version of 3. is also possible but has been omitted for brevity.

Figure 2. *H-DAG with generated directed edges based on evidence from continuous DNA fragments (arrows above nodes from levels* $-4, \ldots, l$ *) that span at least two consecutive levels. Purple paired-end reads are considered discontinuous as the span non-consecutive levels.*

Induced directed edges for discontinuous fragments

After generating all the directed edges based on evidence from the continuous DNA fragments, the H-DAG will most likely contain discontinuities or no directed edges between some adjacent levels. When paired-end read evidence indicates there are discontinuous DNA fragments spanning these levels (see Figure 2), then the HaploMaker algorithm builds new *induced directed edges* to connect the source node containing the heterozygous allele in the left paired end read to the target node containing the heterozygous allele in the right paired end read. For cases where there are overlapping discontinuous fragments the algorithm processes each of them sequentially by the order of their target node levels (from low to high levels) and if two or more fragments have the same target level then the processing is ordered by their source node level (from high to low levels). After processing fragments their induced edges immediately become part of the pre-existing directed edge framework for the H-DAG.

To process an individual discontinuous fragment, we designate the source node as the initial parent node and induce new edges between subsequent levels of the H-DAG using a "look-ahead" pre-order depth first traversal approach [25, 26]. The first component of the approach involves a single level look-ahead algorithm that uses knowledge of the pre-existing directed edges between the source and target nodes to induce new edges to potential children in the adjacent levels of the H-DAG. Once the directed

edges are built, the pre-order depth traversal algorithm then visits each of the children, assigns them as parents and the process is repeated until the target level is encountered.

Due to the simplistic nature of the H-DAG framework, the look-ahead algorithm to find potential children nodes and induce new edges can be easily defined for any two adjacent levels within the source node and target node defined by the discontinuous fragment. Suppose read evidence indicated the source node was v_i^l, with source node sibling $v_j^l,\ i,j \in 1,2\ \ j \neq i$, and traversal was required to target node v_k^m where $m - l > 1$ and $k \in 1,2$. Additionally, let E^{lm} be the set of pre-existing directed edges between levels l and m of the H-DAG. During the recursive depth traversal, consider a parent node v_i^δ where $i \in 1,2$ and $l \leq \delta < m$, then we can define an edge inducing algorithm between adjacent levels δ and $\delta + 1$ that respects pre-existing edges and ambiguity of read evidence, namely

```
getPotentialChildren (parent node v_i^δ, sibling node v_j^δ)
    if v_j^δ has an observed allele AND has directed edges to both child nodes at level δ + 1
        update E^lm to include directed edges from parent node v_i^δ to both nodes at level δ + 1
    else if v_j^δ does not have an observed allele OR has zero or one edge to child nodes at level δ + 1
        for any child node at level δ + 1 with no parent AND is not the sibling of the target node
            update E^lm to include a directed edge from the parent node v_i^δ to that child node
```

Given pre-existing edges in the H-DAG, Figure 3 presents various examples of how the *getPotentialChildren* algorithm induces new edges between a source node and target node. In Figure 3 (A), a previously known directed edge exists between alleles T and C at the l and $l + 1$ levels of the graph, suggesting there is unambiguous phase of the allelic pairs between these levels. The *getPotentialChildren* algorithm indicates we then require a directed edge from the source node with allele A at level l to allele T at the $l + 1$ level to match the unambiguous phase. Using pre-order depth traversal, the node containing allele T becomes the new parent node and a single induced directed edge is generated from T to the target node containing allele G to align with the read evidence from the discontinuous fragment. In Figure 3 (B) the pre-existing directed edges indicate there is phase ambiguity of the allelic pairs between levels l and $l + 1$. The *getPotentialChildren* algorithm then induces directed

edges from the source node containing allele A to both nodes at level $l+1$. The pre-order depth traversal algorithm then sequentially assumes each node at level $l+1$ is a parent node and generates a directed edge from each of the nodes to the target node. Figure 3 (C) follows identically to Figure 3 (B) due to the phase ambiguity of the allelic pairs between levels l and $l+1$ and Figure 3 (D) follows identically from Figure 3 (A).

The combined pre-order depth traversal along with the *getPotentialChildren* method, is then repeated for each discontinuous DNA fragment along the H-DAG. Once complete, the H-DAG has obtained maximum connectivity between levels based on the complete DNA evidence from continuous and discontinuous fragments. Within this final H-DAG, disconnected levels may still exist where there is lack of read evidence to generate directed edges. As a consequence the H-DAG may be partitioned into several sub-graphs based on distinct genomic *blocks* where each block contains consecutive levels of the original H-DAG with at least one directed edge between each level. Without loss of generality a H-DAG block will be defined as a sub-graph $\mathcal{G}_b = (V_b, E_b)$ containing L_b levels, The remaining algorithmic sections will discuss an approach for the numerical estimation of edge weights in a general H-DAG block as well as the path traversal optimisation technique to appropriately phase the heterozygous alleles within each block.

Figure 3: *Various H-DAG possibilities with induced directed edges linking the discontinuous purple DNA fragment from the source node containing allele A at level l to target node containing allele G at level $l+2$. Black edges are pre-existing directed edges; blue edges are new induced directed edges using the getPotentialChildren algorithm.*

Estimating directed edge weights

For each of the directed edges within a H-DAG block, we now derive a locally based probabilistic edge weight function based on cumulative DNA evidence and ambiguity of allelic phase between adjacent levels. Let $v_i^\delta \in V_b$ be a source node within a H-DAG block and $e_{ij}^\delta = \left(v_i^\delta, v_j^{\delta+1}\right) \in E_b, 1 \le \delta < L, i, j \in 1,2$ be an *existing* directed edge between any two adjacent levels. We then define $n(e_{ij}^\delta)$ to be its edge counter, initialized at one, and incremented by one every time e_{ij}^δ is spanned by a DNA

fragment. This initialization provides a convenient mechanism for ascribing a minimum numerical value for induced directed edges generated from discontinuous DNA fragments.

We then define a general probabilistic edge weight function as

$$w_{ij}^{\delta} = pr\left(e_{ij}^{\delta}\right) = \frac{n(e_{ij}^{\delta})}{\sum_k n(e_{ik}^{\delta})} \tag{1}$$

where the denominator is $\sum_k n(e_{ik}^{\delta})$ represents the sum of the existing edge counters emitting from the source node. When there is only one directed edge emitting from the source node, for example where there is an unambiguous phase between adjacent pairs, the numerator and denominator of this edge weight become identical and $w_{ij}^{\delta} = 1$. This immediately indicates that edge weights less than one are derived from adjacent phase ambiguous allelic pairs only.

To exemplify the ambiguous allele pairs case for continuous DNA fragments, consider the $l-1$ and l levels of the H-DAG in Figure 3. The source node v_2^{l-1} containing allele A emits two directed edges, $e_{21}^{l}$and e_{22}^{l}. Let $n(e_{21}^{l})$ and $n(e_{22}^{l})$ indicate their associated edge counts based on the cumulative read evidence for the existence of each directed edge. Using (1) the two edge weights are simply estimated by the local probabilities

$$w_{21}^{l} = \frac{n(e_{21}^{l})}{n(e_{21}^{l}) + n(e_{22}^{l})}\ , \qquad w_{22}^{l} = \frac{n(e_{22}^{l})}{n(e_{21}^{l}) + n(e_{22}^{l})}$$

and have the property, $w_{21}^{l} + w_{22}^{l} = 1$. Similarly, in Figure 3 (C) between the l and $l+1$ levels of the H-DAG there are induced edges formed between a discontinuous DNA fragment and the two edges emitting from source node v_1^{l} indicate some phase ambiguity between the allele pairs. As there are no DNA fragments spanning the edges, the counters $n(w_{12}^{l+1})$ and $n(w_{11}^{l+1})$ would both remain at their initial value of one. Using (1), this immediately indicates the induced edge weights are $w_{12}^{l+1} = w_{11}^{l+1} = 0.5$ and this probabilistic value would be assigned to *all* pairs of induced edges emitting from the same parent node between adjacent levels in the H-DAG.

Minimum weighted path

Once the calculation of all edge weights is complete, a minimum weighted path can be algorithmically determined. Let $P_b = \{p_1, p_2, \ldots, p_t\}$ be the complete set of distinct paths through the block. For any path $p_k \in P_b$, a unique set of nodes $V_{b:k} \subset V_b$, such that $V_{b:k} = \{v_k^1, v_k^2, \ldots, v_k^{L_b}\}b$, are visited across the L_b levels where, at any level of the sub-graph, l_b say, $v_k^{l_b}$ is *one* of the nodes from the pair $(v_1^{l_b}, v_2^{l_b})$. Similar to the complete graph, for the purpose of optimisation, the sub-graph is also capped at each end with dummy nodes $(Start_k, End_k)$ nodes. As the path p_k traverses across a unique set of nodes, it also comprises of a unique set of directed edges defined by $E_{b:k} \subset E_b$ where $E_{b:k} = \{(Start_k, v_k^1), (v_k^1, v_k^2), \ldots, (v_k^{L_b-1}, v_k^{L_b}), (v_k^{L_b}, End_k)\} = \{(Start_k, v_k^1), e_k^1, \ldots, e_k^{L_b-1}, (v_k^{L_b}, End_k)\}$. We define the likelihood of this path as

$$Q(p_k) = pr(p_k) = \prod_{l_b=1}^{L_b-1} pr\left(e_k^{l_b}\right) = \prod_{l_b=1}^{L_b-1} w_k^{l_b} \qquad (2)$$

where $w_k^{l_b} = pr(e_k^{l_b})$, the local probability or weight of the directed edge that traverses from level $l_b - 1$ to level l_b of the H-DAG block. Determining the appropriate phase of the haplotype within the H-DAG block is then equivalent to finding the path with maximum likelihood over the complete set of paths.

For the purpose of utilizing a known path traversal algorithm we can equivalently frame this optimization as a minimization problem. Let $S_b = \{s_1, s_2, \ldots, s_t\}$ be a set of values for the complete set of path traversals through the H-DAG block such that $S(p_k) = -\log Q(p_k)$. Determining the optimal path through the H-DAG block is then equivalent to finding the minimum negative log-likelihood path over the complete set of paths, namely

$$\min_{s_k \in S_b} \{S(p_k), k = 1, \ldots, t;\ S(p_k) = \sum_{l_b=1}^{L_b-1} v_k^{l_b}\} \qquad (3)$$

where $v_k^{l_b} = -\log\left(w_k^{l_b}\right) \geq 0\ \forall\, k, l_b$ define the directed edge weights used in the optimisation algorithm. Optimisation of (3) can then equivalently be viewed as finding the path of minimum weight through the H-DAG block. As the H-DAG block has no directed cycles it can be immediately

topologically sorted and we can then use an established backtracking algorithm to perform the optimisation [16]. This backtracking algorithm is also known to be efficient, requiring $O(n(E_b) + n(V_b))$ linear time, where $n(E_b)$ and $n(V_b)$ are the number of directed edges and nodes in the H-DAG block.

After the backtracking algorithm completes, the first haplotype is obtained by traversing through the H-DAG block on the minimum weighted path and selecting one node at each level on the path. The corresponding second haplotype is then obtained by traversing the minimum weighted path and selecting the alternate allele (the allele not on the path) at each level of the H-DAG. The backtracking algorithmic process is then repeated for each H-DAG block. The algorithm halts once the minimum weighted path is obtained for the final H-DAG block containing the right hand dummy node for the chromosome. Figure 4 presents a flow chart of the complete HaploMaker algorithm for each chromosome from the initial variant calling through to the repeated construction of two sequences for each haplotype block.

Figure 4. *Workflow schematic of the HaploMaker algorithm.*

Algorithm accuracy and statistics

To assess the accuracy of the algorithms to correctly phase the alleles for each haplotype, we used the well-known *switch error* accuracy measure. For any haplotype, the switch error is defined as the number of times the predicted haplotype allele is disconcordant with the true allele obtained from the phased VCF file. The switch error is then averaged as the number of errors per Mbp. As HaploMaker attempts to construct longer haplotypes through inducing new edges, other useful statistics for comparing algorithms were also calculated. These included the well-known N50, the average haplotype length in Mbp and maximum haplotype length achievable with each algorithm. An R based computational implementation of these statistics (comparison.R) is available at [27].

Results

Individual NA12877 paired-end reads

The human reference genome version 38 was used to map 162.6 million (10x) and 407.5 million (25x) paired-end reads from NA12877 individuals with 83.6% and 83.9% of reads aligned concordantly. The average gap-compressed sequence dissimilarity rate was estimated 0.25% and this was similar to previous reports of heterozygosity levels in humans [28]. A total of 1.86% of reads contained small INDELs up to 20bp. The median insert size (DNA fragment length) was estimated to be 316bp. The sorted BAM file along with the NA12877 phased VCF file was then given as input to HaploMaker and the comparative haplotype construction software, HapCompass, HapCUT2 and WhatsHap (see Supplemental Material for execution commands). All algorithms were computationally conducted using a cloud-based Linux instance with 2 cores and 32GB RAM. HaploMaker and HapCompass were the most computationally expedient with HapCUT2 and WhatsHap taking longer to execute.

Table 1. *Comparison of various statistics obtained from the output of the four haplotype phasing algorithms applied to individual NA12877 10x and 25x coverage short paired-end reads.*

	Read Coverage	Switch Error Rate (per Mbp)	N50 (bp)	Average Haplotype Length (bp)	Maximum Haplotype Length (bp)	Running Time (minutes)
HaploMaker	10x	32.1	326	251	2770	10
HapCompass		143.9	329	246	2770	10
HapCUT2		43	296	181	2310	15
WhatsHap		84.2	329	252	2770	35
HaploMaker	25x	38	496	331	26,188	25
HapCompass		157.9	494	326	26,188	30
HapCUT2		47.6	420	269	5,985	30
WhatsHap		77.2	459	307	14,339	135

An R script (comparison.R), available at [27], was used to process the output of the four haplotype algorithms. The switch error rate, the N50 of haplotype blocks and other useful statistics were extracted and presented in Table 1. The table demonstrates HaploMaker had the lowest switch error rate compared

to the other algorithms. For 10x data, HaploMaker, HapCompass and WhatsHap generated equivalent maximum haplotype lengths of 2770bp. For 25x data, HaploMaker and HapCompass generated larger haplotype blocks (up to 26,188bp) compared to the other two algorithms. The table also indicates HapCompass and WhatsHap had substantially higher switch error rates compared to HaploMaker and HapCUT2. Although HapCUT2 had a competitively low switch error rate, it also had shorter and fewer haplotype blocks.

Table 1 also indicates the increase in read coverage to 25x had a negligible effect on the accuracy of all four algorithms with only a slight increase in the switch error rate. However, comparing the N50, average haplotype length and maximum haplotype length, increasing coverage definitively generated longer haplotype blocks with HaploMaker and HapCompass generating the highest average haplotype lengths. Maximum haplotype lengths exceeded 9 times the maximum haplotype length obtained from 10x read coverage.

To more rigorously assess the changes in accuracy as the haplotype block size increased, Figures 5 and 6 presents the average switch error rate of each of the algorithms against a class of haplotype lengths (in number of base pairs). The absolute number of haplotypes in each haplotype length class obtained from the four algorithms are given in Supplementary Table S1 and S2 for the 10x and 25x read coverages respectively. The figures revealed, as haplotype blocks became longer, HaploMaker maintained a competitively low switch error rate compared to the other three algorithms indicating the HaploMaker algorithm preserves accuracy as the size of haplotypes increases. The switch error rate obtained from the HapCUT2 algorithm remained competitive against HaploMaker for shorter haplotype block lengths. However, when the haplotype block length increased to 1500 bp and 3000bp at 10x and 25x coverage respectively, the switch error rate of HapCUT2 became higher than HaploMaker. WhatsHap and HapCompass were the least competitive across all haplotype block size lengths.

Figure 5. *Comparison graph showing the average switch error rate for different haplotype length classes across all four haplotype phasing algorithms applied to individual NA12877 using 10x short paired-end reads.*

Figure 6. *Comparison graph showing the average switch error rate for different haplotype length classes across all four haplotype phasing algorithms applied to individual NA12877 using 25x short paired-end reads.*

Individual NA12878 PacBio CLR/subreads

The total number of PacBio reads was 64 million and their median and mean length was 3611 bp and 5005 bp respectively. Almost 92% of reads contained INDELs and their size varied from 1 up to 10,000 bp. The original alignment to the hg19 human genome reference was performed using BLASR[29] by the authors [22] with sequencing coverage estimated to be 65x. Sequencing error rate of PacBio subreads was reported to be around 14% [30]. Due to the lack of long read PacBio specific settings in HapCompass, we only compared HaploMaker with HapCUT2 and WhatsHap. We used a cloud-based instance of 4 cores and 32GB of RAM. The computational implementation of the HaploMaker algorithm executing in similar time to HapCUT2 and 2.5 times faster than WhatsHap.

An R script (comparison2.R), available at [27]was used to process the output of the three haplotype phasing algorithms. Table 2 contains the switch error rate, the N50 of haplotype blocks and other useful statistics. The table indicates HaploMaker was superior in generating longer haplotypes blocks while maintaining a competitively low switch error rate. In particular, the N50 and average haplotype length obtained from the HaploMaker algorithm was at least 30% longer than HapCUT2 and 300% longer than WhatsHap. These increased haplotype lengths obtained from HaploMaker also ensured greater coverage of the total human reference genome.

Table 2. *Comparison of various statistics obtained from the output of the three haplotype phasing algorithms applied to individual NA12878 PacBio subreads*.

	Switch Error Rate (per Mbp)	N50 (bp)	Average Haplotype Length (bp)	Total Genome Coverage (Gbp)	Maximum Haplotype Length (bp)	Running Time (minutes)
HaploMaker	27.4	46,787	25,490	1.73	351,891	90
HapCUT2	2	37,251	19,560	1.66	299,882	167

WhatsHap	21	14,828	4,737	1.23	265,020	415

Compared to HaploMaker, HapCUT2 had a significantly lower switch error rate and it maintained this reduced rate throughout the partitioned class of haplotype lengths (Figure 7). In this figure we only compared blocks up to 250 Kbp as HaploMaker was the only algorithm to be able to generate longer blocks greater than 250 Kbp (see Supplementary Table S3). Table S3 indicates almost a 2-fold increase in the number of short haplotype block lengths generated by HapCUT2 compared to HaploMaker suggesting for sequencing reads with high error rates, the HaploMaker algorithm trades off some accuracy while it attempts to generate longer haplotype blocks.

Figure 7. *Comparison graph showing the average switch error rate of different haplotype length groups for three haplotype phasing algorithms applied to individual NA12878 PacBio subreads*.

Individual NA12878 PacBio HiFi reads

A total of 1.5 million PacBio HiFi reads with an average length of 10 Kbp were mapped to the human genome reference hg19 using the pbmm2 aligner from Pacific Biosciences [31]. Average gap-compressed sequence identity was 98.6% and this was closer to human genome polymorphism compared to the PacBio subreads. Sequencing coverage was estimated around 5x and the accuracy of HiFi reads was around 99.8%. This is much higher than the accuracy of traditional PacBio subreads [32] and as a consequence the algorithms are expected to construct more accurate haplotype blocks. All three algorithms were executed using identical hardware used to analyse the PacBio subread data. As there were much fewer reads compared to previous experiments, all three algorithms had reduced execution times. HaploMaker completed in 5 minutes with HapCUT2 and WhatsHap completing in 14 and 18 minutes (see Supplemental Material for execution commands).

An R script (comparison3.R), available at [27] was used to process the output of the three haplotype phasing algorithms and Table 3 contains the switch error and other useful output statistics. Due to the sequence accuracy of the HiFi reads, the switch error rates of all three methods were significantly lower compared to the switch error rates obtained from analysing the PacBio subreads and paired-end read

data sets. Despite reduced sequencing coverage, HaploMaker managed to construct at least two times longer average haplotype lengths and N50 than the competing algorithms. As a result HaploMaker assembled a 1.54 Gbp genome compared to 1.34Gbp and 1.14 Gbp genomes generated from the two other algorithms, a gain of up to 35% coverage of the human genome.

Table 3. *Comparison of various statistics obtained from the output of the three haplotype phasing algorithms applied to individual NA12878 PacBio HiFi reads*.

	Switch Error Rate (per Mbp)	N50 (bp)	Average Haplotype Length (bp)	Total Genome Coverage (Gbp)	Maximum Haplotype Length (bp)	Running Time (minutes)
HaploMaker	2.67	31,698	15,340	1.54	315,905	5
HapCUT2	1.45	14,081	6,816	1.34	140,731	14
WhatsHap	4.54	12,542	4,369	1.14	183,560	18

Figure 8 presents the switch error rate of each of the algorithms against the haplotype length with the number of haplotype lengths in each class given in Supplementary Table S4. In the figure we only compared blocks up to 180 Kbp as HaploMaker was the only algorithm able to generate longer blocks greater than 180 Kbp (see Supplementary Table S4). The figure indicates both HaploMaker and WhatsHap maintained low switch error rates across all length classes with HaploMaker being more accurate. HapCUT2 had the lowest switch error rate up to 120 Kbp but then generated a significantly larger switch error rate (10x worse) for 120+ Kbp haplotypes compared to other two algorithms. Similar to the PacBio subread experiment, WhatsHap and HapCUT2 generated substantially more shorter haplotype blocks than HaploMaker. However, in contrast to the subread experiment, HaploMaker maintained a low switch error rate suggesting the quality of HaploMakers haplotype phasing algorithm is dramatically increased when the accuracy of the sequencing reads is improved.

Figure 8. *Comparison graph showing the average switch error rate of different haplotype length groups for three haplotype phasing algorithms applied to individual NA12878 PacBio HiFi reads*.

Discussion

In this research we presented an improved reference sequence-based haplotype construction algorithm, HaploMaker, that accurately assembles haplotypes of diploid genomic sequences by framing the problem as an edge weighted DAG and phasing haplotypes using optimal path traversal algorithmic techniques. The novel strategy of inducing new directed edges based on read evidence to resolve disconnected levels of the H-DAG enabled HaploMaker to accurately phase longer genomic regions compared to other leading reference-based sequence algorithms, HapCUT2, HapCompass and WhatsHap. For PacBio CLR data, the longer haplotype lengths generated by HaploMaker resulted in an increased switch error rate compared to the other algorithms suggesting that HaploMaker trades off accuracy for haplotype length when read sequencing error is high. However, When longer and more accurate PacBio HiFi reads were used, the average haplotype block lengths assembled were substantially greater using HaploMaker with negligible sacrifice in accuracy. In all experiments analysed here, longer block lengths generated by the algorithm also ensured greater coverage of the genome. From a computational standpoint, the HaploMaker algorithm was shown to scale well with a significant reduction in computing time when longer, more computationally intensive PacBio reads were used. It is also important to note the phasing of INDEL polymorphisms has been incorporated into the HaploMaker algorithm and it maintained a highly competitive accuracy. This is a crucial aspect of the algorithm as INDELs were the major source of switch errors from the haplotype construction software compared here and this issue is also exacerbated when read sequences, such as PacBio subreads, have a high sequencing error rate [12].

Graph based frameworks have been widely adopted for population and reference based haplotype assembly of genomes [7, 15, 33]. In this research we focussed on defining the problem as a simplistic directed acyclic graph that removes the bulk of the complexity many graph based haplotype phasing algorithms contain [15, 34]. The look ahead edge inducing algorithm contained in HaploMaker bares

resemblance to the divide and conquer strategy used in the spectral graph based approach of [33] except HaploMaker re-connects pairs of vertices when there is sufficient read evidence to do so, resulting in longer haplotype blocks. When read sequencing error is high this generation of longer haplotypes comes at the cost of reduced accuracy and we are now currently exploring small amendments to the HaploMaker algorithm to ensure the accuracy remains competitive.

The length of assembled haplotypes depends on level of genome heterozygosity, DNA read/fragment length and coverage of sequencing used. The human genome has significantly lower level of heterozygosity compared to other diploid organisms such as *Arabidopsis thaliana* [35]. Therefore we can expect shorter assembled haplotypes from human genomic sequences. In our example, using DNA fragments (insert size) of 316bp and a paired-end sequencing coverage of 10x and 25x, the HaploMaker algorithm achieved a maximum haplotype length of 2,770 bp and 26,188 bp respectively. While these lengths are short relative to the total length of a human chromosome, it is sufficient to enable PCR primer and CRISPR/Cas9 guide RNA based experiments where a short homologue specific sequence around a genomic position is required [36], [37]. In contrast, when using PacBio with read lengths of 5 Kbp average, the HaploMaker algorithm managed to assemble haplotypes up to 350 Kbp. This result emphasizes the importance of sequencing longer reads if a greater haplotype length is required [1].

We have demonstrated the ability of the HaploMaker algorithm to accurately assemble human diploid genomic sequences and its potential is now being explored for other areas of related genomic sequence research. For example, the algorithm could be used to assemble haplotypes for genomes from tetraploid and hexaploid species, such as wheat, as long as there are separate reference sequences for each of the homoeologous copies of the chromosomes [1]. In cases where a set of genomic sequences have been generated from a population of related individuals, we are exploring the use of the base algorithm of HaploMaker for discovering the most recurring haplotypes among the population.

Conclusion

By framing the haplotype assembly problem as a DAG and using a novel edge inducing strategy for discontinuous DNA fragments, the HaploMaker algorithm was able to accurately phase long haplotype

blocks using short or long sequence reads. The algorithm was shown to be highly efficient and also has potential to be impactful in similar genomic sequence research areas where accurate haplotype phasing or selection is required. To ensure the portability of the HaploMaker algorithm across varying computing architectures it has been implemented in Java and is available under MIT license from [27].

Availability of Supporting Data and Materials

The two FastQ files and the sorted BAM file relating to the NA12877 individuals are accessible through NCBI SRA at [38]. The VCF files relating to the individuals NA12877 and NA12878 are publicly accessible through the Figshare repository at [39]. The human genome reference and its Bowtie2 index was downloaded from the link within [40].

The Bowtie read alignment software version 2.4.1 was downloaded from [41]. Comparative haplotype assembly software, HapCompass version 0.8.2 was downloaded from [42] and required Java 1.8 or higher to execute. HapCUT2 1.3.3 was compiled from [43] and for execution required installation of high-throughput sequencing tools library htslib from [44]. WhatsHap version 1.1 (latest) depends on python version 3.6 or higher and C++ compiler and was installed using pip. pbmm2 version 1.7 was downloaded and installed from [31].

Availability of Source Code and Requirements

The HaploMaker source code and Java executable (MFbio.jar file) are publicly accessible from [27] under MIT license. The resulting haplotype output files for all four algorithms are accessible from Figshare [39]. R code to process the output files and generate reports is located at [27] in *comparison.R, comparison2.R* and *comparison3.R.*

An archival copy of the code and supporting data is available via the GigaScience database, GigaDB [45]

Project Name	HaploMaker
Project Home Page	https://github.com/mfruzan/HaploMaker

Operating System	Platform Independent
Programming Language	Java
Requirements	Java 1.8 or higher
License	MIT
RRID	SCR_021928
Biotools ID	haplomaker

Abbreviations

SNP: Single Nucleotide Polymorphism; DAG: Directed acyclic graph; H-DAG: Haplotype DAG; bp: base pair; Mbp: Mega base pair; MEC: Minimum Error Correction; INDEL: Insertion Deletion; PCR: Polymerase Chain Reaction; CRISPR: Clustered Regularly Interspaced Short Palindromic Repeat; VCF: Variant Calling Format; RNA: Ribonucleic acid; PacBio: Pacific Biosciences; CCS: Circular Consensus Sequencing; HiFi: High Fidelity; CLR: Continuous Long Reads;

Competing interests

The authors declare no competing interests.

Authors Contributions

MF and JT designed the algorithm. MF, WT, OK and JT wrote the manuscript. MF wrote source code in Java. MF and JT wrote source code in R. MF performed analysis.

Acknowledgements

The authors gratefully acknowledge the Grain Research and Development Corporation (GRDC) of Australia for supporting this research. We also acknowledge the support from all the staff specially Russell Edson in the Biometry Hub the School of Agriculture, Food and Wine, University of Adelaide.

Lastly, we are enormously grateful to the three reviewers for their comments that have enabled us to dramatically improve this manuscript.

References

1. Garg S. Computational methods for chromosome-scale haplotype reconstruction. Genome biology. 2021;22 1:1-24.
2. Berger E, Yorukoglu D, Peng J and Berger B. Haptree: A novel bayesian framework for single individual polyplotyping using ngs data. PLoS computational biology. 2014;10 3:e1003502.
3. Garg S, Fungtammasan A, Carroll A, Chou M, Schmitt A, Zhou X, et al. Chromosome-scale, haplotype-resolved assembly of human genomes. Nature biotechnology. 2021;39 3:309-12.
4. Excoffier L and Slatkin M. Maximum-likelihood estimation of molecular haplotype frequencies in a diploid population. Molecular biology and evolution. 1995;12 5:921-7.
5. Delaneau O, Coulonges C and Zagury J-F. Shape-IT: new rapid and accurate algorithm for haplotype inference. BMC bioinformatics. 2008;9 1:540.
6. Kong A, Masson G, Frigge ML, Gylfason A, Zusmanovich P, Thorleifsson G, et al. Detection of sharing by descent, long-range phasing and haplotype imputation. Nature genetics. 2008;40 9:1068.
7. Browning SR and Browning BL. Rapid and accurate haplotype phasing and missing-data inference for whole-genome association studies by use of localized haplotype clustering. The American Journal of Human Genetics. 2007;81 5:1084-97.
8. Delaneau O, Zagury J-F and Marchini J. Improved whole-chromosome phasing for disease and population genetic studies. Nature methods. 2013;10 1:5-6.
9. Delaneau O, Zagury J-F, Robinson MR, Marchini JL and Dermitzakis ET. Accurate, scalable and integrative haplotype estimation. Nature communications. 2019;10 1:1-10.
10. Majidian S, Kahaei MH and de Ridder D. Minimum error correction-based haplotype assembly: Considerations for long read data. Plos one. 2020;15 6:e0234470.
11. Bansal V and Bafna V. HapCUT: an efficient and accurate algorithm for the haplotype assembly problem. Bioinformatics. 2008;24 16:i153-i9.
12. Edge P, Bafna V and Bansal V. HapCUT2: robust and accurate haplotype assembly for diverse sequencing technologies. Genome research. 2017;27 5:801-12.
13. Das S and Vikalo H. SDhaP: haplotype assembly for diploids and polyploids via semi-definite programming. BMC genomics. 2015;16 1:260.
14. Patterson M, Marschall T, Pisanti N, Van Iersel L, Stougie L, Klau GW, et al. WhatsHap: weighted haplotype assembly for future-generation sequencing reads. Journal of Computational Biology. 2015;22 6:498-509.
15. Aguiar D and Istrail S. HapCompass: a fast cycle basis algorithm for accurate haplotype assembly of sequence data. Journal of Computational Biology. 2012;19 6:577-90.
16. Cormen TH, Leiserson CE, Rivest RL and Stein C. Introduction to algorithms. MIT press; 2009.
17. Eberle MA, Fritzilas E, Krusche P, Källberg M, Moore BL, Bekritsky MA, et al. A reference data set of 5.4 million phased human variants validated by genetic inheritance from sequencing a three-generation 17-member pedigree. Genome research. 2017;27 1:157-64.
18. Sedlazeck FJ, Lee H, Darby CA and Schatz MC. Piercing the dark matter: bioinformatics of long-range sequencing and mapping. Nature Reviews Genetics. 2018;19 6:329-46.
19. EMBL's European Bioinformatics Institute. https://www.ebi.ac.uk/ena/browser/view/PRJEB3381. Accessed 23 March 2022.
20. Illumina Platinum Genomes. https://sapac.illumina.com/platinumgenomes.html. Accessed 23 March 2022.

21. Langmead B and Salzberg SL. Fast gapped-read alignment with Bowtie 2. Nature methods. 2012;9 4:357.
22. Zook JM, Catoe D, McDaniel J, Vang L, Spies N, Sidow A, et al. Extensive sequencing of seven human genomes to characterize benchmark reference materials. Scientific data. 2016;3 1:1-26.
23. NCBI ftp Mt Sinai. ftp://ftp-trace.ncbi.nlm.nih.gov/giab/ftp/data/NA12878/NA12878_PacBio_MtSinai. Accessed 23 Macrh 2022.
24. UCSC Human Genome 19. http://hgdownload.cse.ucsc.edu/goldenPath/hg19/bigZips/chromFa.tar.gz. Accessed 23 March 2019.
25. Kural M. Tree traversal and word order. Linguistic Inquiry. 2005;36 3:367-87.
26. Morris JM. Traversing binary trees simply and cheaply. Information Processing Letters. 1979;9 5:197-200.
27. HaploMaker (2022).: https://github.com/mfruzan/HaploMaker/.
28. Bryc K, Patterson N and Reich D. A novel approach to estimating heterozygosity from low-coverage genome sequence. Genetics. 2013;195 2:553-61.
29. Chaisson MJ and Tesler G. Mapping single molecule sequencing reads using basic local alignment with successive refinement (BLASR): application and theory. BMC bioinformatics. 2012;13 1:1-18.
30. Weirather JL, de Cesare M, Wang Y, Piazza P, Sebastiano V, Wang X-J, et al. Comprehensive comparison of Pacific Biosciences and Oxford Nanopore Technologies and their applications to transcriptome analysis. F1000Research. 2017;6.
31. Biosciences P: pbmm2. https://github.com/PacificBiosciences/pbmm2.
32. Wenger AM, Peluso P, Rowell WJ, Chang P-C, Hall RJ, Concepcion GT, et al. Accurate circular consensus long-read sequencing improves variant detection and assembly of a human genome. Nature biotechnology. 2019;37 10:1155-62.
33. Yu Y, Chen L, Miao X and Li SC. SpecHap: a diploid phasing algorithm based on spectral graph theory. Nucleic Acids Research. 2021;49 19:e114-e. doi:10.1093/nar/gkab709.
34. Garg S, Aach J, Li H, Sebenius I, Durbin R and Church G. A haplotype-aware de novo assembly of related individuals using pedigree sequence graph. Bioinformatics. 2020;36 8:2385-92. doi:10.1093/bioinformatics/btz942.
35. Tyagi A, Singh S, Mishra P, Singh A, Tripathi AM, Jena SN, et al. Genetic diversity and population structure of Arabidopsis thaliana along an altitudinal gradient. AoB Plants. 2016;8.
36. Lv J, Wu S, Wei R, Li Y, Jin J, Mu Y, et al. The length of guide RNA and target DNA heteroduplex effects on CRISPR/Cas9 mediated genome editing efficiency in porcine cells. Journal of veterinary science. 2019;20 3.
37. Ye J, Coulouris G, Zaretskaya I, Cutcutache I, Rozen S and Madden TL. Primer-BLAST: a tool to design target-specific primers for polymerase chain reaction. BMC bioinformatics. 2012;13 1:1-11.
38. NCBI SRA. https://www.ncbi.nlm.nih.gov/sra/PRJNA664648. Accessed 22 March 2022.
39. The University of Adelaide Figshare . https://doi.org/10.25909/12972761. Accessed 23 March 2022.
40. Illumina igenome (Human Genome Reference) : https://sapac.support.illumina.com/sequencing/sequencing_software/igenome.html. Accessed 23/03/2022.
41. Bowtie2 (2.4.1) https://sourceforge.net/projects/bowtie-bio/files/bowtie2/2.4.1/.
42. Hapcompass (0.8.2) https://www.brown.edu/Research/Istrail_Lab/hapcompass.php.
43. HapCUT2 (1.3.3) https://github.com/vibansal/HapCUT2.
44. SAMtools, htslib. https://github.com/samtools/htslib.

45. Fruzangohar M; Timmins WA; Kravchuk O; Taylor J : Supporting data for "HaploMaker: An improved algorithm for rapid haplotype assembly of genomic sequences" GigaScience Database. 2022. http://dx.doi.org/10.5524/102206.

Supplementary data

Supp_Material_RevisedV2.docx

Figure1 Click here to access/download;Figure;Figure1.jpg

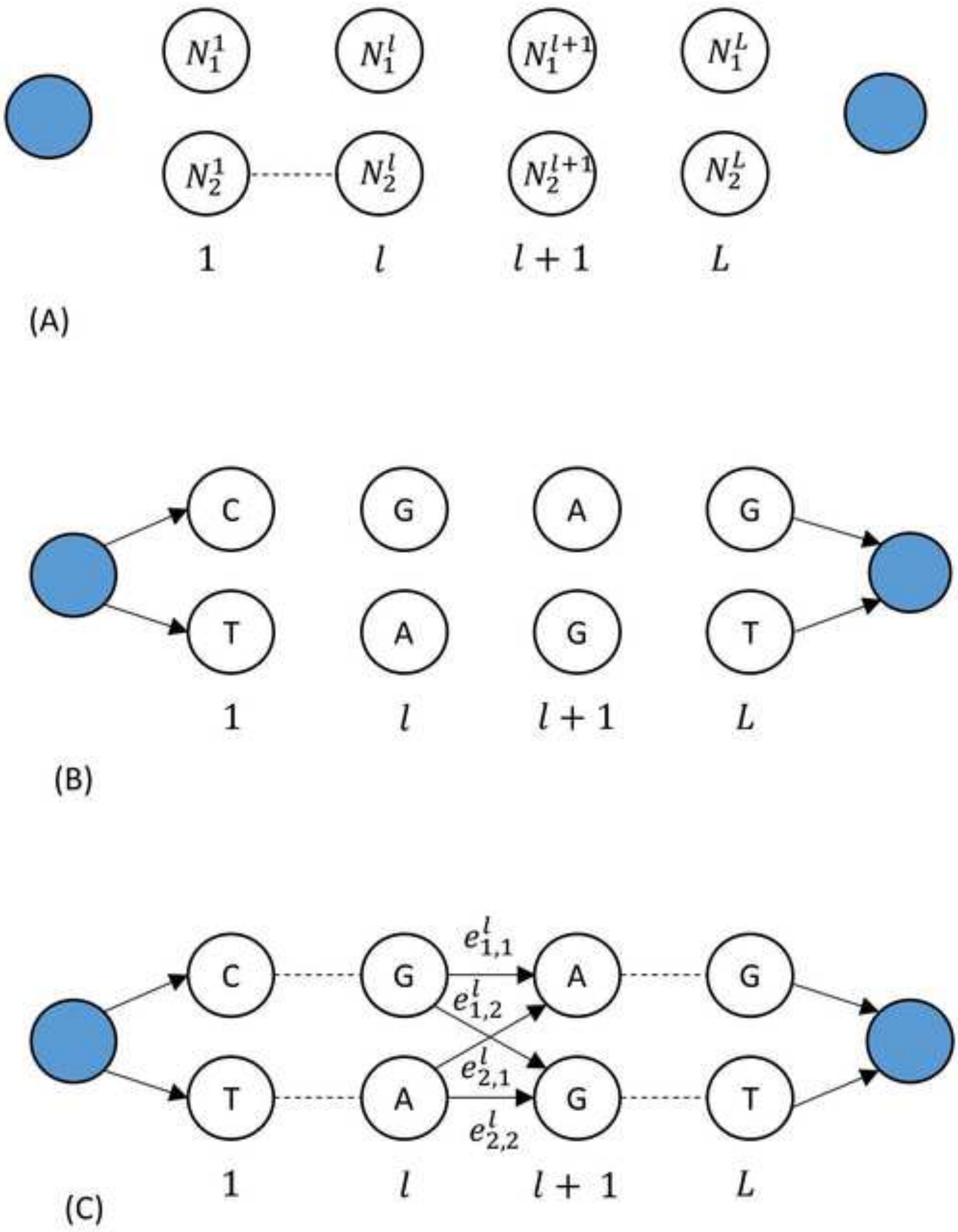

Figure2

Click here to access/download;Figure;Figure2.jpg

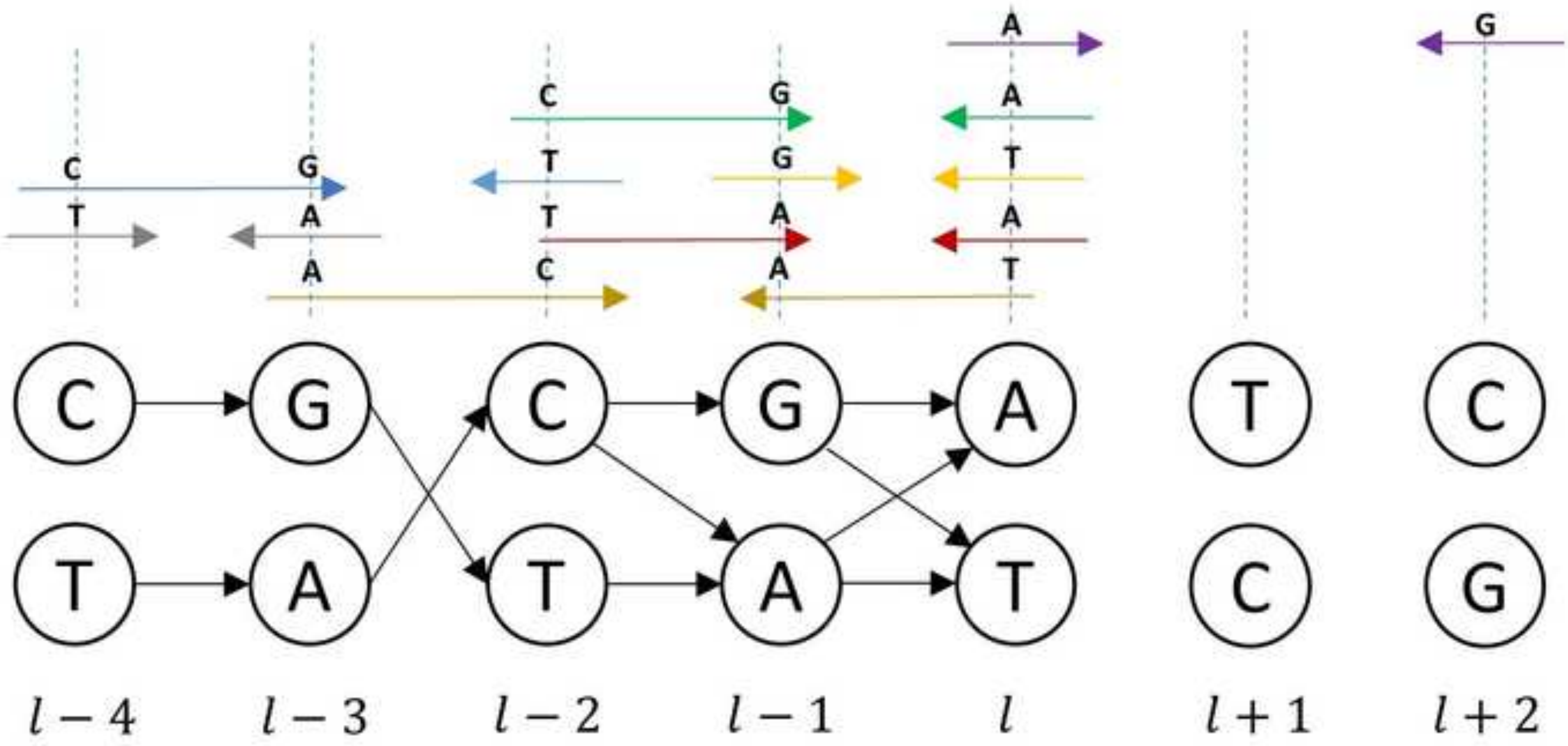

Figure3
Click here to access/download;Figure;Figure3.jpg

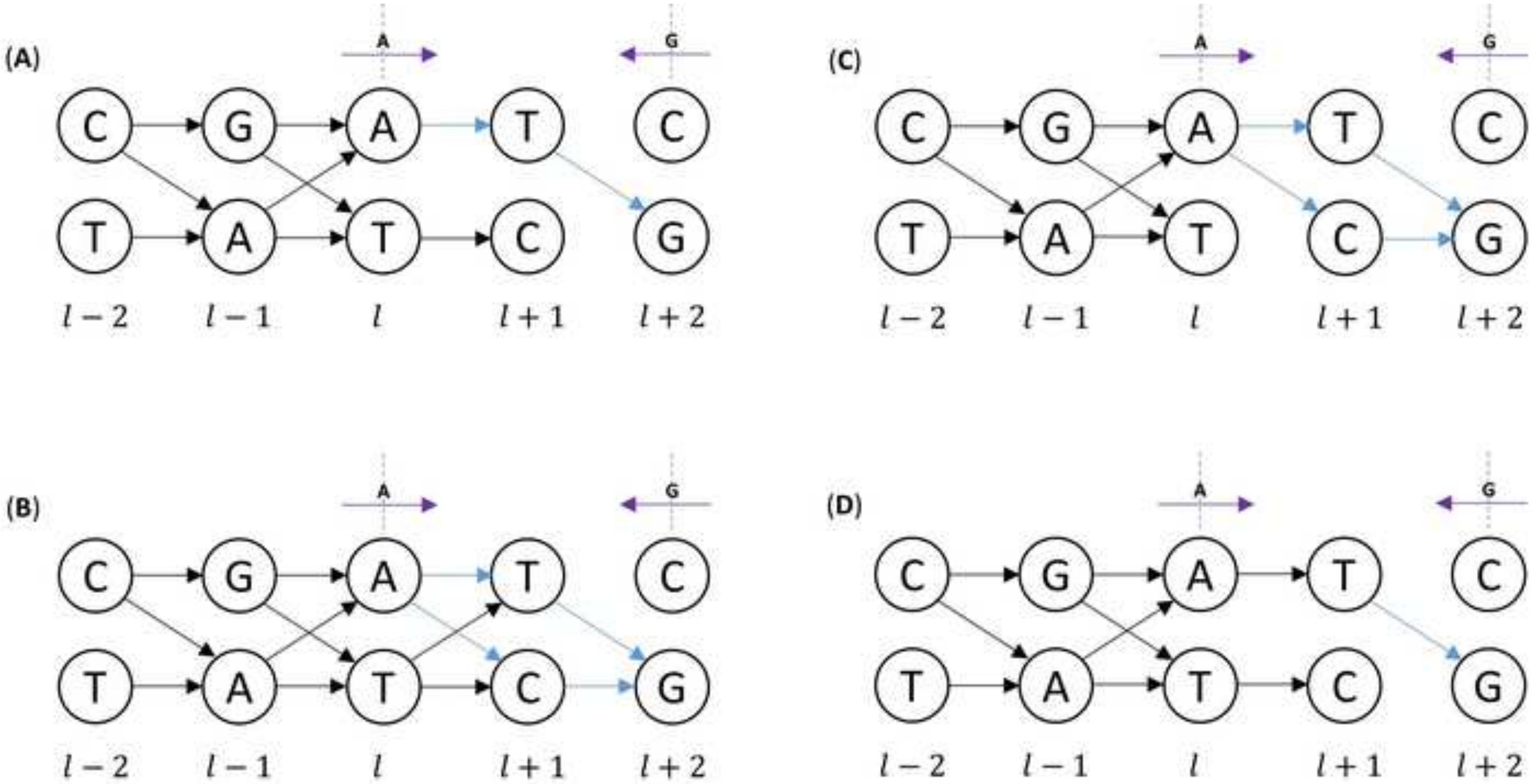

Click here to access/download;Figure;Figure4.jpg

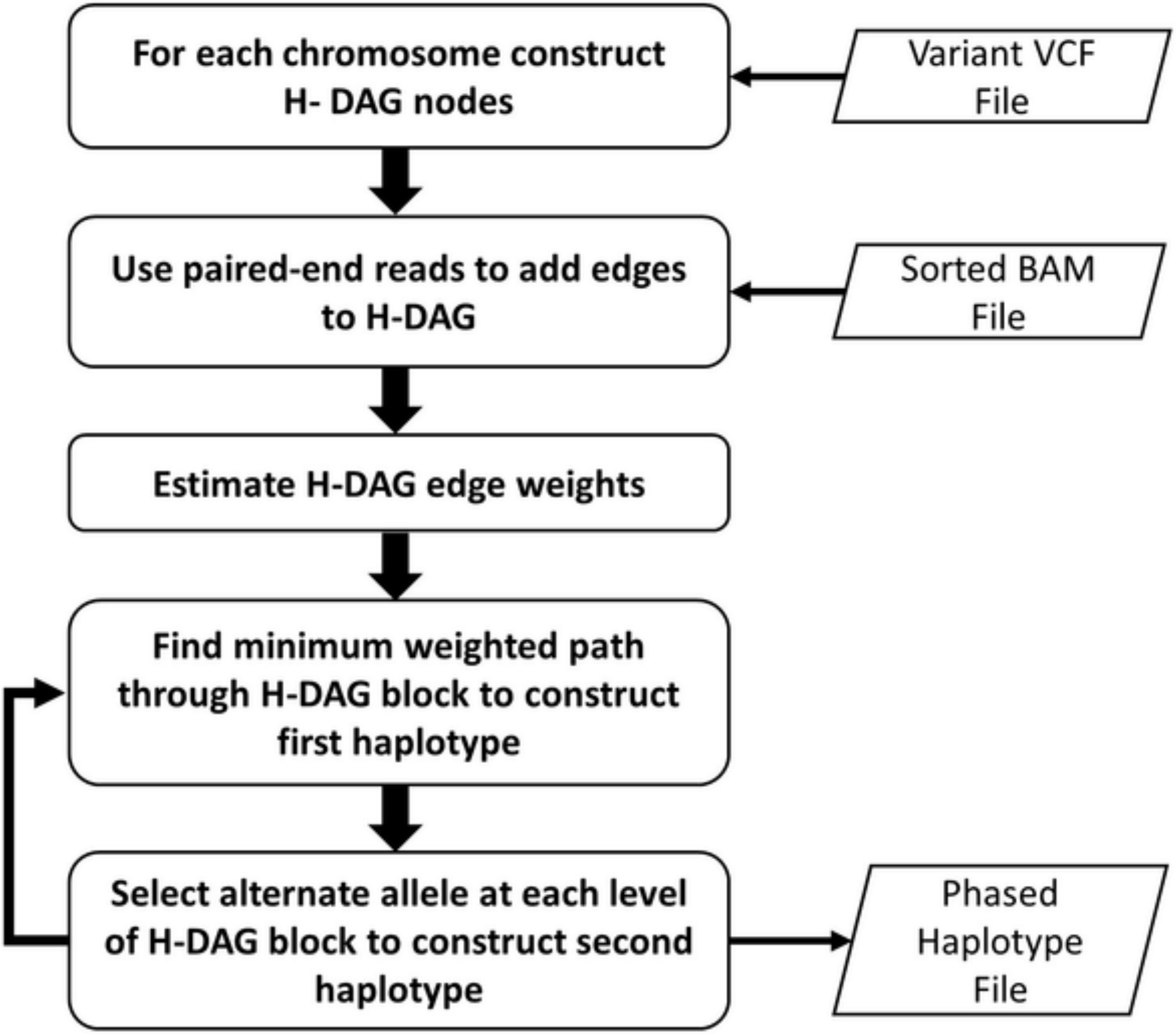

Figure5

Click here to access/download;Figure;Figure5.jpeg

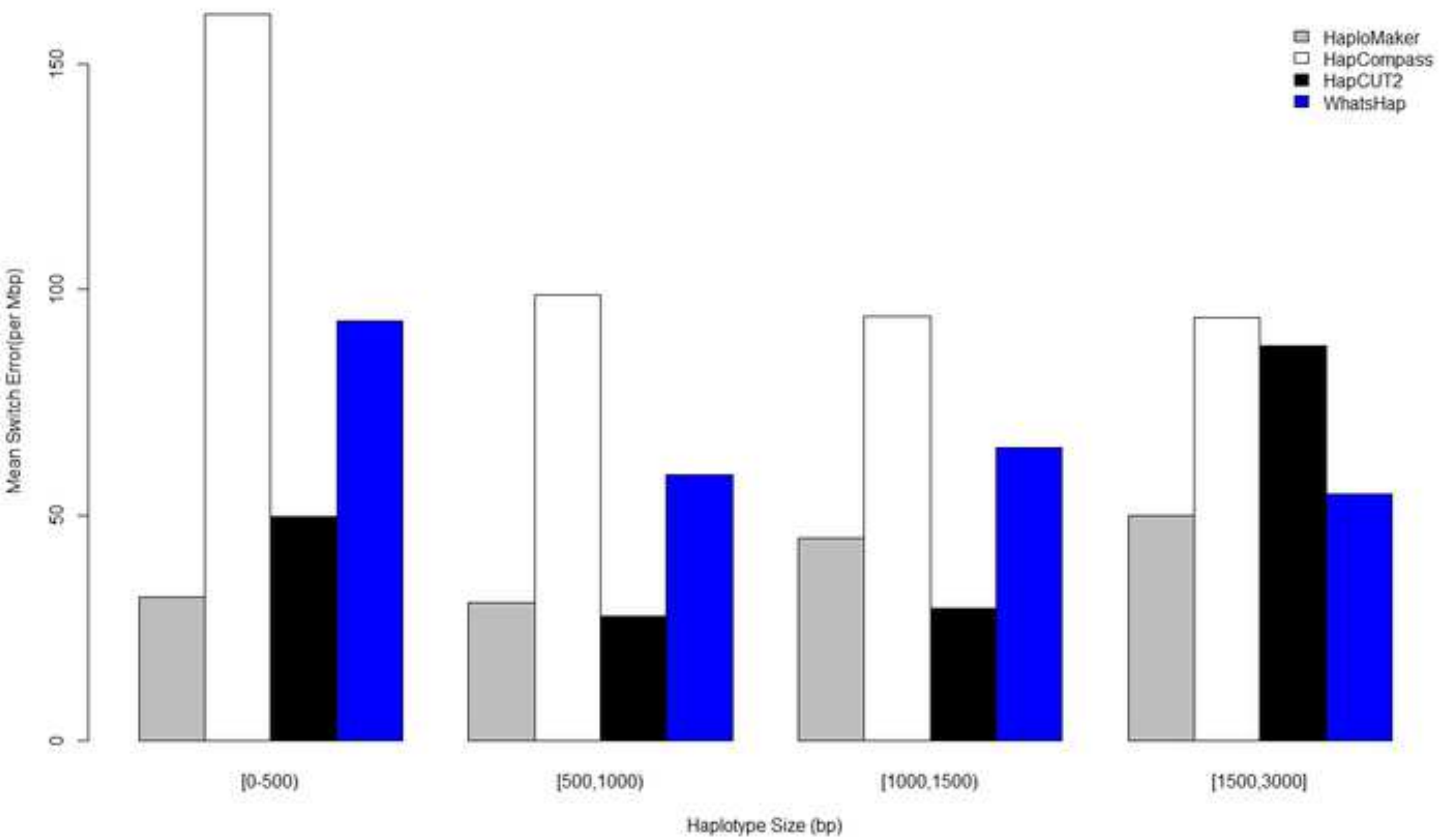

Figure6

Click here to access/download;Figure;Figure6.jpeg

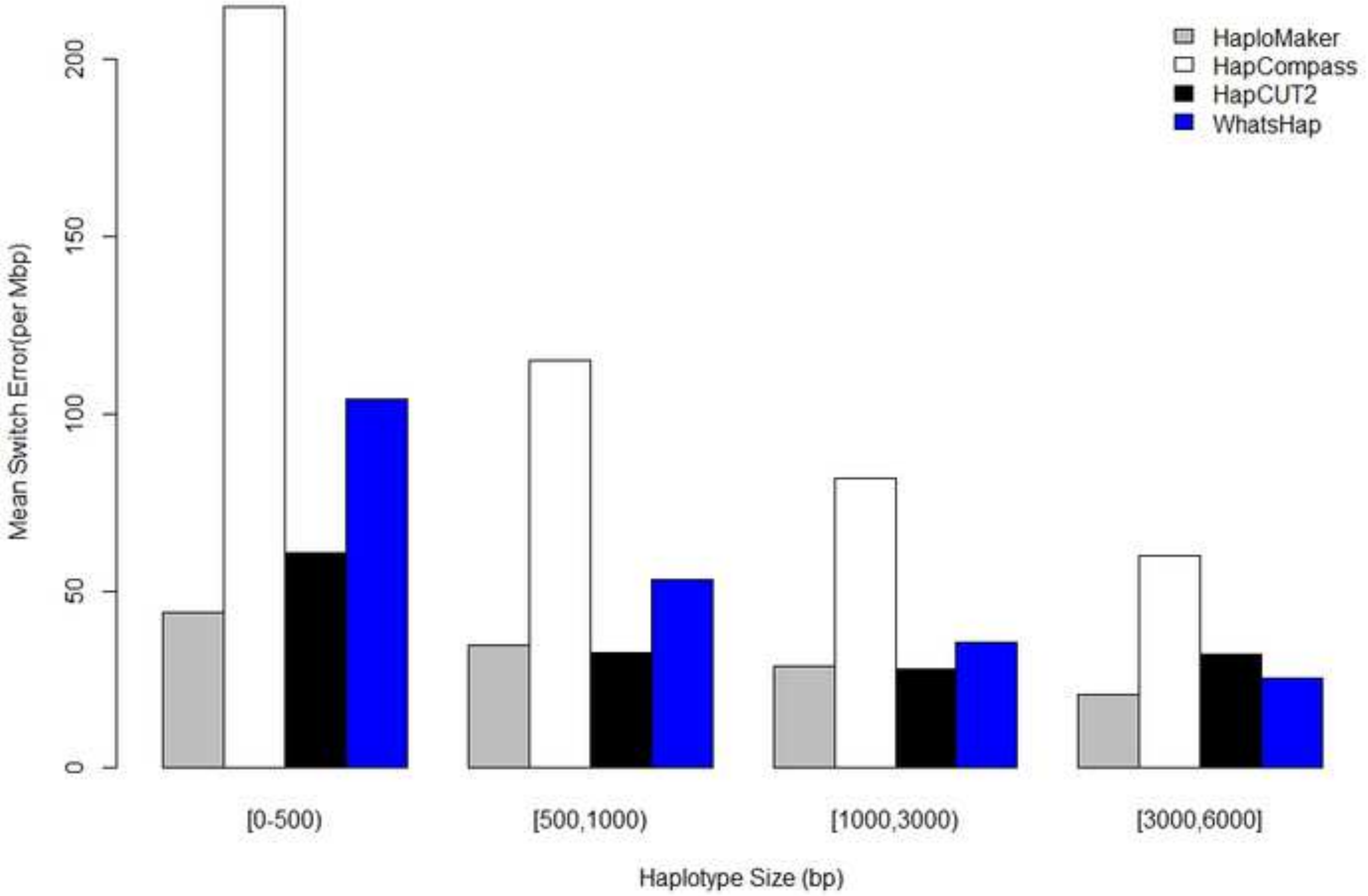

Figure7

Click here to access/download;Figure;Figure7.jpeg

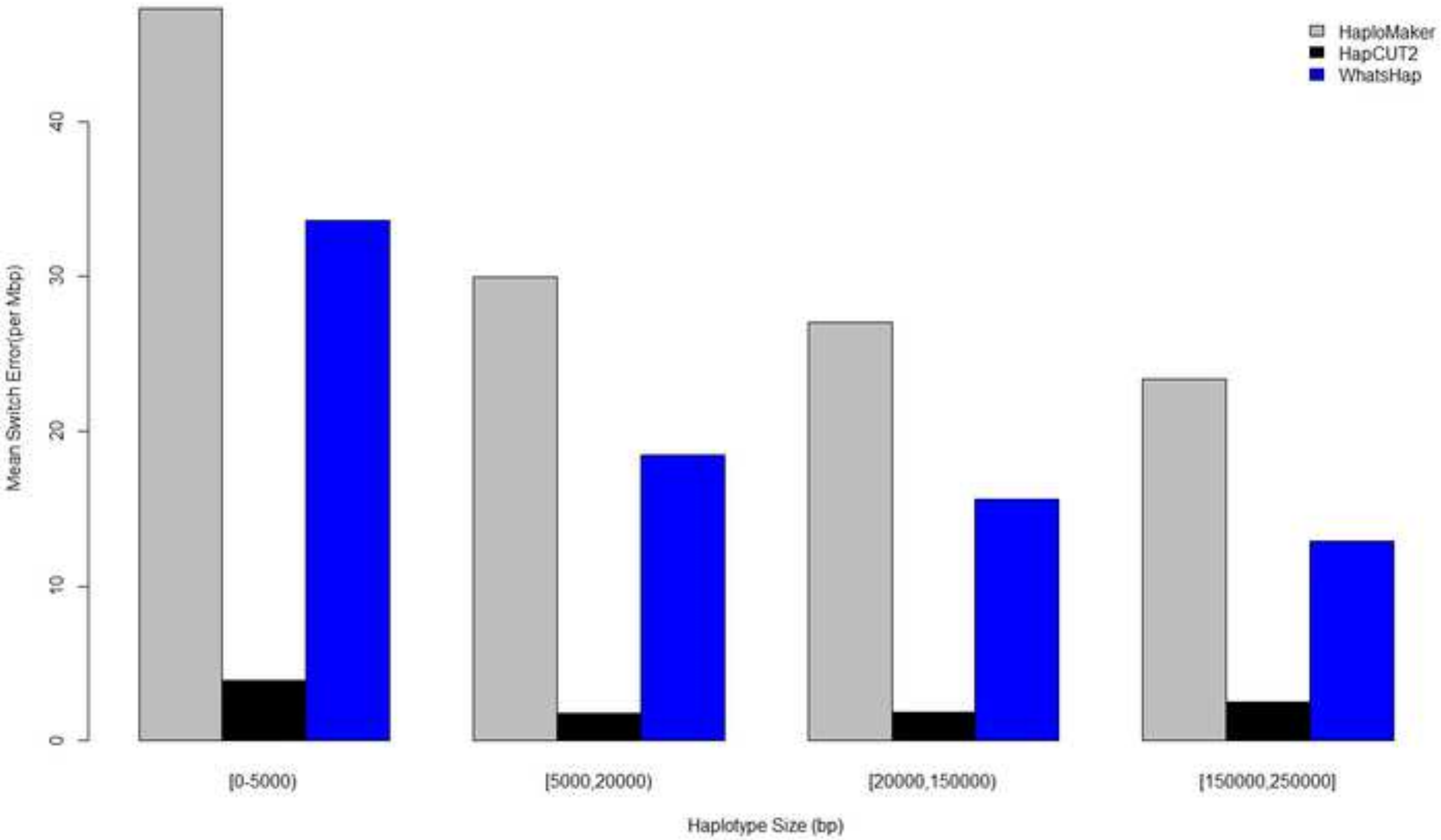

Figure8 Click here to access/download;Figure;Figure8.jpeg

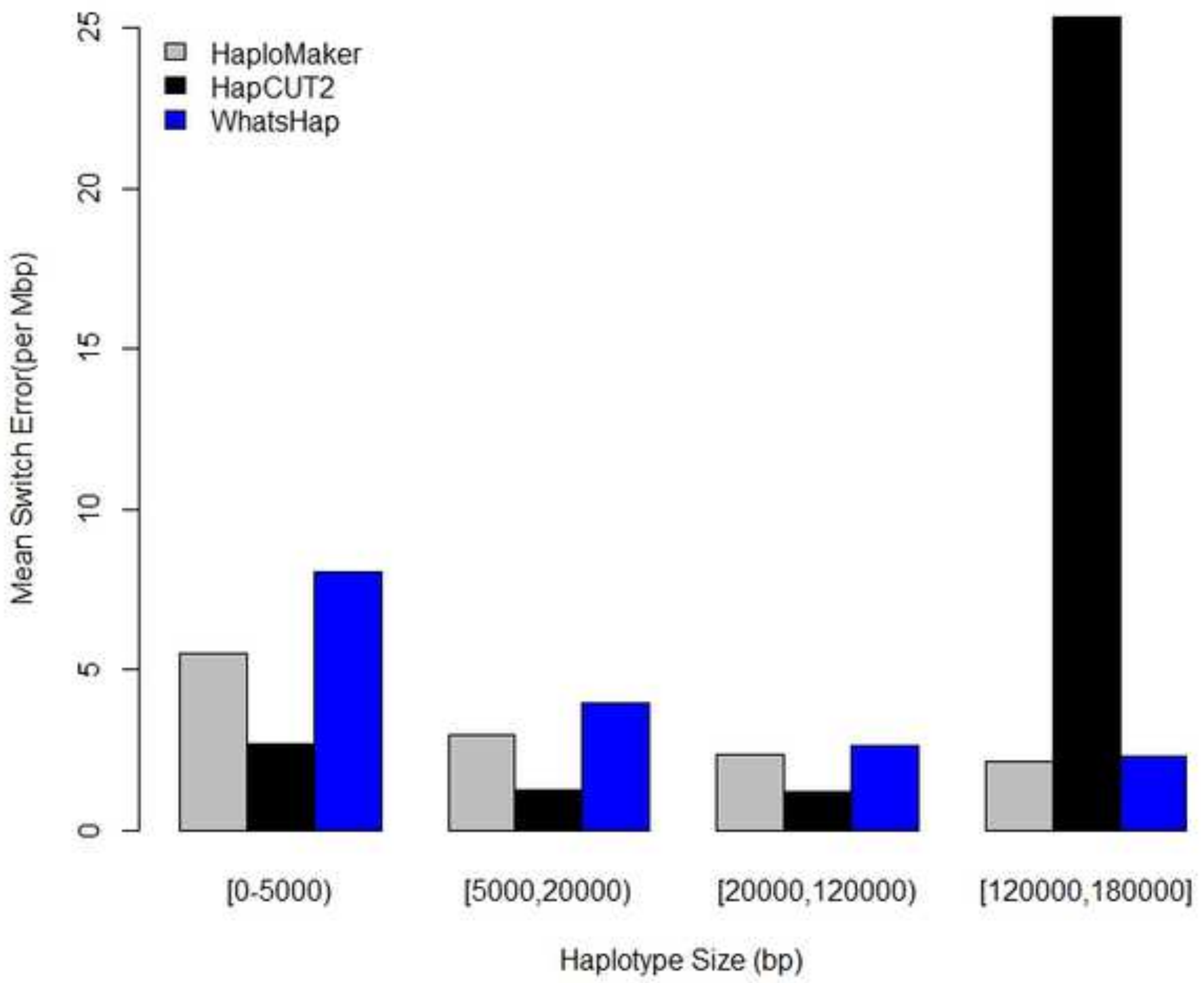

Click here to access/download
Supplementary Material
Supp_Material_RevisedV2.docx
