# Supplementary material for: HaploMaker: An improved algorithm for rapid haplotype assembly of genomic sequences
Source: Gigascience. 2022 May 17;11:giac038. doi: 10.1093/gigascience/giac038 (PMC9112781; doi:10.1093/gigascience/giac038)
Supplement: giac038_Supplemental_File [file giac038_supplemental_file.docx]

**Supplemental Material**

HaploMaker: An improved algorithm for rapid haplotype assembly of genomic sequences

Mario Fruzangohar^1*^, William A. Timmins^1^, Olena Kravchuk^1^, Julian Taylor^1^

^1^The Biometry Hub, School of Agriculture, Food and Wine, University of Adelaide, Australia

^*^Corresponding author

Email: mario.fruzangohar@adelaide.edu.au

**Table of Contents**

[Mapping to Human Genome 2](#_Toc93324816)

[Algorithm Execution 2](#_Toc93324817)

[HaploMaker 2](#_Toc93324818)

[Individual NA12877 paired-end reads 2](#_Toc93324819)

[Individual NA12878 PacBio subreads 3](#_Toc93324820)

[Individual NA12878 PacBio HiFi (CCS) reads 3](#_Toc93324821)

[HapCompass 3](#_Toc93324822)

[HapCUT2 4](#_Toc93324823)

[Individual NA12877 paired-end reads 4](#_Toc93324824)

[Individual NA12878 PacBio reads 4](#_Toc93324825)

[WhatsHap 4](#_Toc93324826)

[Individual NA12877 paired-end reads 5](#_Toc93324827)

[Individual NA12878 PacBio reads 5](#_Toc93324828)

[Haplotype frequencies 5](#_Toc93324829)

# Mapping to Human Genome

Mapping the NA12877 FastQ files to the Human reference Genome version 38 was conducted using Bowitie2 version 2.4. 1 as follows:

bowtie2 -x hg38_igenome/Homo_sapiens/NCBI/GRCh38/Sequence/Bowtie2Index/genome -1 NA12877_R1.fastq.gz -2 NA12877_R2.fastq.gz --end-to-end --very-sensitive --n-ceil L,0,0.1 --rdg 20,1 --rfg 20,1 --no-unal --no-mixed --mp 40,40 --np 5 --ignore-quals --score-min L,0,-0.4 --threads 16 | samtools view -bS - > NA12877.bam;

The BAM file was then sorted and all the reads that had MAPQ less than five were removed, resulting in a file called NA12877_q5.bam. As HapCut2 failed to process NA12877.vcf given NA12877_q5.bam, we had to obtain a subset of SNPs inside NA12877.vcf that were covered by NA12877_q5.bam. We called this VCF file commonmerge.vcf.

Mapping the NA12878 PacBio HiFi FastQ files to the Human reference Genome version hg19 was conducted using pbmm2 version 1.7 as follows:

pbmm2 align /references/UCSC_hg19/minimap2/wholegenome.mmi /pacbiohifiNA12878/SRR9001768.1.fastq.gz /pacbiohifiNA12878/mapped.bam --preset CCS --sort -j 12 -J 4 --log-level INFO

# Algorithm Execution

## HaploMaker

### Individual NA12877 paired-end reads

java –Xmx30g -jar MFbio.jar –-task diploidhap -–vcf commonmerge.vcf –-out haplomaker.hap --afl 400 --seqtype pairedend --minmapq 5 --maxmapq 42 –-ref ~/hg38_igenome/Homo_sapiens/NCBI/GRCh38/Sequence/WholeGenomeFasta/genome.fa --refx genome.fa.fai –-bam NA12877_q5.bam

Parameter Description:

vcf: VCF file

out: output haplotype file

ref: Reference file

refx: Reference file index

bam: sorted BAM file

afl: Average fragment length

seqtype: pairedend, clr, hifi

minmapq: Minimum read quality MAPQ

maxmapq: Maximum MAPQ used by the aligner

Please note that MFbio.jar contains the hts-jdk.jar file from <https://github.com/samtools/htsjdk> project.

### Individual NA12878 PacBio subreads

java –Xmx30g -jar MFbio.jar --task diploidhap --vcf NA12878_sorted.vcf --out NA12878.hap --afl 5000 --seqtype clr --minmapq 5 --maxmapq 50 --ref ~/references/UCSC_hg19/wholegenome.fa --refx ~/references/UCSC_hg19/wholegenome.fa.fai --bam ~/NA12878/pacbio/sorted_final_merged.bam

Note: Parameter seqtype is set to ‘clr ‘for PacBio subreads. Parameter ‘maxmapq’ was set to 50 showing maximum MAPQ used in BLASR aligner.

### Individual NA12878 PacBio HiFi (CCS) reads

java –Xmx30g -jar MFbio.jar --task diploidhap --vcf NA12878_sorted.vcf --out NA12878.hap --afl 5000 --seqtype hifi --minmapq 5 --maxmapq 60 --ref ~/references/UCSC_hg19/wholegenome.fa --refx ~/references/UCSC_hg19/wholegenome.fa.fai --bam ~/NA12878/pacbio/sorted_final_merged.bam

Note: Parameter ‘seqtype’ is set to ‘hifi’ for PacBio HiFi reads. Parameter ‘maxmapq’ was set to 60 showing maximum MAPQ used in pbmm2 aligner.

## HapCompass

java -Xmx3000m -jar ~/hapcompass_v0.8.2/hapcompass.jar --bam NA12877_q5.bam --vcf commonmerge.vcf -o hapcompass_output

The output was in the hapcompass_output folder and was renamed to hapcompass_commonmerge_q5.txt for the sake of consistency.

## HapCUT2

The execution of the HapCUT2 algorithm requires the installation of the high-throughput sequencing tools library, htslib version 1.2.1 or higher.

### Individual NA12877 paired-end reads

Preceding the HapCUT2 execution an initial extractHAIRS command was executed to obtain haplotype fragments.

extractHAIRS --mmq 5 –indels 1 --bam NA12877_q5.bam --VCF commonmerge.vcf --out commonmerge_fragments

where mmq parameter sets minimum MAPQ of reads in BAM file. Then the output of this executed command was used in

HAPCUT2 --fragments commonmerge_fragments --VCF commonmerge.vcf --output hapcut2_commonmerge_q5.hap

### Individual NA12878 PacBio reads

Hapcut2 processes Pacbio reads in 3 steps as follows:

Step 1:

extractHAIRS --pacbio 1 --mmq 5 –indels 1 --bam sorted_final_merged.bam --VCF NA12878_sorted.vcf --out NA12878_fragments --ref ~/references/UCSC_hg19/wholegenome.fa

Step 2:

HAPCUT2 --fragments NA12878_fragments --VCF NA12878_sorted.vcf --output hapcut2.hap

Step 3:

python3 utilities/prune_haplotype.py -i hapcut2.hap -o hapcut2_pruned.hap --min_mismatch_qual 30 --min_switch_qual 30

## WhatsHap

The execution of the WhatsHap algorithm requires the installation of python 3.6 or higher and the C++ compiler.

### Individual NA12877 paired-end reads

whatshap phase -o whatshap_phased_commonmerge.vcf --ignore-read-groups --indels –reference ~/hg38_igenome/Homo_sapiens/NCBI/GRCh38/Sequence/WholeGenomeFasta/genome.fa commonmerge.vcf NA12877_q5.bam

### Individual NA12878 PacBio reads

whatshap phase -o NA12878_phased.vcf --ignore-read-groups --indels --reference ~/references/UCSC_hg19/wholegenome.fa NA12878_sorted.vcf sorted_final_merged.bam

# Haplotype frequencies

**Table S1***: Frequency of haplotypes across four haplotype length classes obtained from the four haplotype phasing algorithms applied to individual NA12877 10x coverage short paired end reads.*

| Length Group | HaploMaker | HapCompass | HapCUT2 | WhatsHap |
| --- | --- | --- | --- | --- |
| 0-500 | 248,161 | 300,298 | 315,315 | 293,461 |
| 500-1000 | 23,294 | 30,009 | 15,968 | 28,366 |
| 1000-1500 | 1,836 | 2,479 | 710 | 2,240 |
| 1500-3000 | 231 | 338 | 41 | 295 |

**Table S2***: Frequency of haplotypes across four haplotype length classes obtained from the four haplotype phasing algorithms applied to individual NA12877 25x coverage short paired end reads.*

| Length Group | HaploMaker | HapCompass | HapCUT2 | WhatsHap |
| --- | --- | --- | --- | --- |
| 0-500 | 335,336 | 393,770 | 405,896 | 398,511 |
| 500-1000 | 58,780 | 69,127 | 54,290 | 62,836 |
| 1000-3000 | 17,500 | 20,203 | 11,778 | 16,411 |
| 3000-6000 | 664 | 795 | 141 | 478 |
| 6000-30000 | 48 | 63 | 0 | 27 |

**Table S3***: Frequency of haplotypes across four haplotype length classes obtained from the three haplotype phasing algorithms applied to individual NA12877 PacBio subreads..*

| Length Group | HaploMaker | HapCUT2 | WhatsHap |
| --- | --- | --- | --- |
| 0-5000 | 13,904 | 24,426 | 200,647 |
| 5000-20000 | 25,390 | 31,971 | 45,457 |
| 20000-150000 | 28,090 | 28,684 | 13,661 |
| 150000-250000 | 398 | 184 | 32 |
| 250000-400000 | 27 | 0 | 1 |

**Table S4***: Frequency of haplotypes across four haplotype length classes obtained from the three haplotype phasing algorithms applied to individual NA12877 PacBio HiFi reads.*

| Length Group | HaploMaker | HapCUT2 | WhatsHap |
| --- | --- | --- | --- |
| 0-5000 | 38,307 | 108,142 | 204,075 |
| 5000-20000 | 35,757 | 67,841 | 47,150 |
| 20000-120000 | 26,309 | 14,267 | 11,635 |
| 120000-180000 | 216 | 2 | 24 |
| 180000-350000 | 17 | 0 | 1 |
