# Supplementary material for: HaploMaker: An improved algorithm for rapid haplotype assembly of genomic sequences
Source: Gigascience. 2022 May 17;11:giac038. doi: 10.1093/gigascience/giac038 (PMC9112781; doi:10.1093/gigascience/giac038)
Supplement: giac038_Response_to_Reviewer_Comments_Original_Submission [file giac038_response_to_reviewer_comments_original_submission.pdf]

Author's Response To Reviewer Comments

Close

The authors would like to thank the reviewers for extremely useful comments that have helped us dramatically improve the manuscript. Firstly, we need to point out that we have changed the name of the algorithm to “HaploMaker” to avoid conflicting with historically licensed software Haplominer. These changes have been made in the manuscript, supplementary material and this reviewer report.
In this new version we have focussed on improving the notation and formalization of the H-DAG framework and provide some useful definitions when we think they are needed. We have kept and expanded the graphical representations of the H-DAG used in Figures 1 to 3 as we believe these provide useful pictorial representations of the more formal H-DAG framework and definitions. Additionally, we have improved text explanations of various aspect of the HaploMaker algorithm to help answer reviewer queries. As all the reviewers have recognized, HaploMaker is quite simple and we believe this simplicity is one of the key features of the algorithm. For this reason we have purposely shied away from adding more graph complexity to the article when aspects of the algorithm can be formally justified through well-established citable material.
Also in this revised version we have included additional experiments to help answer reviewer queries. There is now a paired-end read experiment that includes 25x coverage of reads to help understand how the accuracy (as well as other statistics) of HaploMaker and other algorithms might differ when read coverage is increased. In addition, the manuscript also includes an experiment involving high quality PacBio HiFi reads to improve the understanding of algorithm accuracy when sequencing accuracy is dramatically increased. The discussion of both of these new experiments has been added to the relevant sections of the manuscript.
Specific responses to reviewer queries are given below.

Reviewer #1:
In this article, the authors presented a novel reference-based haplotype assembly algorithm named HaploMaker in order to acquire long phased haplotypes with more accuracy. Importantly, this algorithm uses a directed acyclic graph structure to incorporate SNPs as nodes and weighted read evidences as edges, and converts the haplotype phasing task to an mathematical optimization problem. The authors then showed it worked well with both paired-end short reads and longer PacBio reads for NA12878 when compared with other algorithms. The idea of this algorithm is interesting, if its performance is solid, it would be promising to be further developed for much longer reads assembly.

Specific points:
1. In Page6 and line 89, I understand the authors want to point the potential ability of this algorithm, but they should change their statement in a more appropriate way. Since heterozygous positions are not always close, especially for species with large genome but low heterozygosity rate, and their algorithm are mainly designed for paired-end short reads assembly or pacbio reads, it would still be difficult to phase DNA strands "of any length". And at the results section, they didn't evaluate the algorithm on that, so it's not appropriate to say it "has the ability to phase DNA strands of any length …".
>This has been corrected in the text.

2. In Page15 and line 298, "HaploMaker, HapCompass and Hapcut2 comparably generated a similar number of haplotype blocks, 322K and 339K and 324K respectively … of 2770bp". The statement is conflict with Table 1. Not sure which one is correct. Please confirm and change it accordingly.
>Thank you for identifying this issue. This has been corrected in the text.

4. Since the haplotype path finding has been framed into a probability-based optimization problem that heavily based on the read evidences, so if the coverage depth will or will not influence the algorithm performance? and how? Please present experiments on algorithm performance with coverage.
>Thank you for this useful comment. Based on this we have conducted an additional experiment using the paired-end read data, increasing the coverage by 2.5 times. We observed significant increase in the assembled haplotype length. We added these new results and discussion to the manuscript.

5. The authors should provide experiments with PacBio HiFi data. See data available from paper:

https://www.nature.com/articles/s41587-020-0711-0.
>Thank you for the article link. We have used data from this paper to conduct a new experiment and the results have been added to the manuscript.

6. The authors didn't provide enough details about PacBio reads information, such as average mismatch rate, as they stated for short reads dataset. If provided, it will help the reader to know the quality of PacBio reads tested in this study.
>Additional information regarding the coverage and quality of reads was added to the relevant sections of the manuscript.

7. The authors should perform experiments for other genomes that are used widely for phasing studies. See review article on genome and public datasets:
https://genomebiology.biomedcentral.com/articles/10.1186/s13059-021-02328-9
Unfortunately, there are no gold standard phased VCF files for other genomes and we wanted to focus on the accuracy of HaploMaker and the other algorithms in this article.

8. Please present the command lines used for experiments.
>All commands are provided in the Supplementary File.

Decision: Major revision

Reviewer #2:
This paper deals with haplotype assembly, which refers to reconstructing haplotypes from sequencing reads using applicable reference coordinates. Haplotype assembly partitions reads into groups reflecting their ancestral origin, and based on the grouping, infers the variants that characterize the ancestors. The bulk of work has been focusing on diploid genomes, and so does this paper. Haplotype assembly has been dealt with in various ways, many of which reflect a higher degree of sophistication in terms of computer science.

This paper presents a new framework based on directed acyclic graphs having clearly identifiable levels, allowing for nodes being (not just partially) ordered.

As for results in their own right, this paper does not appear to deliver any real progress. In fact, HaploMaker, the new approach presented is largely on a par with the state of the art approaches, with some advantages on the one hand, and some disadvantages on the other hand.

However, what intrigues me is the simplicity of the approach in comparison with earlier approaches, and the flexibility in terms of allowing for more than just two phases (haplotypes). Earlier approaches often are very sophisticated, which apparently seems to imply that earlier approaches cannot be flexibly extended to more than 2 haplotypes.

Given that, I am positive about this paper. I believe that the framework presented can deliver inspiration for future haplotype assembly approaches, also addressing polyploid settings.

What I found regrettable however was the lack of clarity in terms of sketching the computational problems and their algorithmic solutions in terms of disambiguous, formal notation. Given that the value of this paper (to the best of my understanding) is in the straightforwardness and simplicity of the approach, it would be great if the theoretically interested reader could clearly identify the computational problems and solutions involved, as per providing appropriate formal descriptions of the computational problems and embedding them into the corresponding theory.

This does not mean that I do not appreciate the author's work in other ways: everything is perfectly reproducible for example, not lacking any clarity in this respect. I am only regretting that the theoretical / algorithmic concepts have not been embedded into the theoretical landscape of problems.

MAJOR:

* As already mentioned, an clearer embedding of the computational problems and solutions may be crucial to really understand the value of this paper.
* For example, what exactly do the optimization problems look like the algorithms behind HaploMaker are referring to, and how do they relate with known theoretical problems? (computing the "minimum weighted path" for example)

>We appreciate the reviewers comments and have overhauled a substantial amount of the methods sections to better align with formal graph notation and theory. We also believe there is beauty in the simplicity of the HaploMaker algorithm and have purposely steered away from adding unnecessary complexity to the article when it can be formally justified through well-established citable material. One of the examples of this is the use of a backtracking algorithm to find the “minimum weighted path”. We have provided the formal lead up and optimisation problem with equation (3) and cited the well-established backtracking algorithm as the tool to conduct this optimisation.

* What is the exact definition of a 'switch error' in this paper?
>We have included an additional sub-section at the end of the Methods that summarises the switch error accuracy measure and other statistics used in the Results sections.

MINOR:

* "transforms the haplotype construction problem into a directed acyclic graph (DAG) structure" sounds strange (transforming a problem into a graph structure) What do you really mean?
>We have amended the text to something more suitable.

* "The algorithm has the ability to phase DNA strands of any length as long as there are heterozygous positions close enough to be covered by at least a single DNA fragment." This is hard to believe (I actually don't), something is wrong here. Without having fragments connecting heterozygous positions, phasing is impossible. Having positions close enough is not sufficient.
>This has been amended in the text to something clearer that is accurate for both the continuous and discontinuous DNA fragments cases we discuss in the article.

* "the HaploMaker algorithm runs in O(n)": What is n actually defined to be?
>We have removed this from the text as it uses information that requires definitions to be stated in advance.

* "processor power": what is that? (never heard that before)
>We have amended this to ‘processing power’.

* "HaploMaker algorithm was capable of constructing the most accurate haplotype blocks while maintaining length of haplotypes. Additionally, when using longer PacBio reads, HaploMaker generated significantly longer haplotype blocks while maintaining a competitively low switch error rate.": This is very imprecise summary. I would appreciate a clearer summary of achievements.
>Given the large number of changes in the manuscript, we have amended the text to something more suitable. However, we have kept this succinct as it is the Introduction and we provide greater depth in the Results.

* "(a DNA read and its pair)": what is 'its pair' supposed to mean exactly?
>This refers to illumina paired-end sequencing technology, where a DNA fragment of average length 400bp is read from both ends of the fragment and have a known left and right pairing to each other. We have amended this in text to ensure there is no ambiguity.

* "if it spanned consecutive levels of the H-DAG without loss of coverage": what does 'loss of coverage' mean in this context?
>In the context of the sentence the reviewer has highlighted, loss of coverage is referring to the lack of mapped read coverage across the heterozygous alleles assigned at a level of the H-DAG.

* assignation -> assignment
>Amended.

Reviewer #3: The authors present a new algorithm, called HaploMaker, for reconstructing haplotypes of a diploid individual based on sequenced reads. HaploMaker reduces the problem to the problem of finding a minimum weight path in a weighted DAG built using the input reads. Interesting is their approach that adds edges when a read presents evidence that two non-contiguous alleles are in-phase. The authors provide a basic experimental evaluation of their approach compared to other state-of-the-art approaches (HapCut2, HapCompass, WhatsHap). This assessment partially supports the claim that HaploMaker is competitive with the other approaches, while being significantly faster.

The computational problem is relevant, the exposition is generally clear, and the proposed approach, albeit algorithmically simple, is sound.

I have some major concerns and I think the authors should address them with a revision of the manuscript.

Formalization of the approach. Definitions of the structures used in HaploMaker are not provided in a formal and declarative way. For example, when the H-DAG is introduced (line 121 and following), the authors never says that each input variant site is associated with two nodes of the graph (with a level, using their terminology) and they say that edges only connect nodes of adjacent levels only in the following section. These parts are important in order to understand the approach and they can only be inferred by using the (useful) examples depicted in the figures. In general, I found that the exposition is more operational than declarative. For example, when weights are assigned to edges (line 223 and following) it is described how counters are computed rather than defining what the counters are. In this way, it is not clear if induced edges have counters equal to 1 or not. A definition would have made clear that.

>These are very helpful comments from the reviewer and we have decided to re-write most of the methods sections to align the notation of the paper with well-established graph theory and to provide more formal declarations of the H-DAG where needed. We have kept or expanded most of the figures as they provide useful pictorial examples of the more formal declarations we have used.
Notation. The mathematical notations used in the manuscript are not the ones widely used in graph theory and/or they do not appear to be formally correct or consistent. For example, at line 124 the set of nodes is defined as set of L pairs, but edges are (presumably) defined over the elements that compose the pairs (and not the pairs themselves). Furthermore, I found rather strange that L is included in the triplet that defines the graph, as it can be deduced by the cardinality of the node set. Also, the notation for edges is new to me (see, for example, line 158): edges are generally represented as pairs, while the notation used in the manuscript usually represents functions/maps. Using the same capital letter for denoting sets and their elements (see, for example, the set of nodes - line 124 - or the set of paths -line 243) does not help readability. For a formal point of view, using Q_k and S_k to denote the likelihood and the negative log-likelihood of path P_k is an
abuse of notation: using functions - q(P_k) and s(P_k), for example - is a more appropriate choice in my opinion. Carefully revising the notation would improve the readability of the manuscript.
>We are very appreciative of the reviewer for these comments and we have substantially overhauled the notation of the H-DAG throughout the methods sections to better align with formal graph theory.

Clarity/correctness of algorithm for inducing edges. I appreciate the idea of adding potential edges when there is indirect evidence that two non-adjacent alleles are in-phase (derived from paired-end reads, for example). However, I cannot fully understand the algorithm that has been proposed. I do understand the general idea, but I do not understand how the cases where read pairs intersect each other (say, a read pair connects level l with l+2, another read pair connects level l+1 with level l+3). Does the order of operations influence the final results? How the algorithm getPotentialChildren is applied? I think that this part is relevant for the novelty of the proposed approach, hence a more detailed and more formal description would improve the quality of the work.
>The reviewer has provided some valuable comments. We have completely overhauled this section to help answer the reviewers queries and to provide a clearer explanation of how this part of the algorithm works. Specifically, we have included additional information in the manuscript explaining the order discontinuous fragments are processed. We have also added some formality to the notation surrounding the getPotentialChildren algorithm but kept the pseudo-code simple as it provides a clear explanation of how edges are formed.

Formal analysis of the time complexity. The authors claim that their approach runs in linear time (line 92-93). However, no formal analysis of the time complexity is presented. What represents n in line 92?
>We have removed this from the text as it uses information that requires definitions to be stated in advance.

Limited experimental evaluation. The authors present a limited experimental evaluation of the approach. In particular:

- The results of the experiments on short reads are a bit disappointing. The input reads have an insert size of 336bp but the average reconstructed haplotype length is approximately 250bp (for all the methods). I wonder if the input data are sufficient to test the effectiveness of the haplotype

reconstruction methods. Probably, checking coverages higher than 15x as used in the manuscript could help.
>To address the reviewers query we have conducted an additional experiment using the paired-end read data. We used a new FastQ file with 25x coverage and, compared to the original 10x coverage, found the length of assembled haplotypes increased by 10 times. We have added the new analysis and its results to the manuscript and discussed the effect of coverage in relevant sections.

- On PacBio long reads, HaploMaker reconstructs longer haplotypes than the other methods but with a higher switch error rate. I wonder if the greater haplotype length is obtained since HaploMaker connects pairs of adjacent variant sites that the other methods instead "cut" since they think there is not enough evidence for phasing across these sites. In other words, the other methods might be more "conservative" than HaploMaker. To answer to this question, it would be interesting to investigate the distribution of the length of parts of haplotypes that do not contain switch errors (i.e., parts of haplotypes that were correctly phased).
>We have conducted a new experiment to further support the accuracy of HaploMaker. In the new dataset, HiFi PacBio reads with accuracy around 99.8% were used and the switch error rate of HaploMaker was significantly lower while still maintaining substantially longer haplotypes.

- I also suggest adding the GATK HaplotypeCaller to the comparison, since it is based on a formulation different from the one on which the other methods are based. Such addition would better highlight the relative merits of the different approaches.
>In an earlier version of this manuscript we used HaplotypeCaller and, compared to the algorithms used in this research, the results were disappointing. It was computationally sluggish and the length of haplotypes did not exceed 440bp. HaplotypeCaller first performs full De Bruijn -based assembly for a small region (which is time consuming) and then it constructs artificially long reads (using bamout option) that represented haplotypes. We contacted the authors about these results and they indicated the reason for these issues was the fact it was purpose built for SNP/Variant calling and leveraging short local haplotypes to increase the accuracy of the variant calls.

Minor points:

Please double check the links to the files hosted at GitHub, as some of them do not work.

>This has been amended.
Fig 5 and 6. Could you please also include (in a different figure/table) the number of haplotypes reconstructed by each method in each class? I think it is useful in order to avoid comparing means over sets of different sizes.
>We have added a set of tables to the Supplementary material that include counts of haplotypes in each class across all algorithms and experiments conducted in this article.

While the computational analysis is adequately described in the text and in the supplementary material, such description is not easily reproducible. Adding scripts for performing (either in the main repository or in a companion repo) the analysis and for recreating the environment (see, for example, conda) would further improve the work.
>We have uploaded all the necessary code in GitHub.

Close
