# Supplementary material for: HaploMaker: An improved algorithm for rapid haplotype assembly of genomic sequences
Source: Gigascience. 2022 May 17;11:giac038. doi: 10.1093/gigascience/giac038 (PMC9112781; doi:10.1093/gigascience/giac038)
Supplement: giac038_Response_to_Reviewer_Comments_Revision_1 [file giac038_response_to_reviewer_comments_revision_1.pdf]

Author's Response To Reviewer Comments

Close

Reviewer #1: Thank you for addressing comments. However, there are a few minor comments:
De novo phasing methods using graphs seems important to be discussed (https://academic.oup.com/bioinformatics/article/36/8/2385/5682413) for future perspectives.
2. A recent review article (https://genomebiology.biomedcentral.com/articles/10.1186/s13059-021-02328-9) on phasing is recommended to be cited in the introduction.
>Thank you for mentioning these articles, we have also found them very helpful and referred to them in relevant places of our manuscript.

Reviewer #2: In general, I am very glad about how the authors improved the paper! I remain with two minor comments:

* I remain confused about the definition of 'continuous'. You write:

"During the processing of the read evidence, a DNA fragment (matching left and right paired-end reads) was considered to be continuous if it spanned consecutive levels of the H-DAG without loss of coverage across the heterozygous alleles contained in those levels. This is exemplified in Figure 2 where the (− 4,..., + 2) levels of the example skeleton H-DAG from Figure 1 are used. The red, green and yellow paired-end reads are considered continuous DNA fragments as they span consecutive levels (− 4,...,) of the H-DAG with read evidence indicating they also contain the heterozygous alleles in those levels."

First, it reads as if a read spans consecutive levels without loss of coverage, which does not make much sense (one read makes for coverage 1, I mean). Second, the red, green and yellow paired-end reads, other than indicated, only span levels l-2, l-1, l in Figure 2. Maybe the latter is the source of my confusion?
>We understand the reviewers confusion and have amended the text to make it clearer.

* 'Processing power' is just as unclear as 'processor power'. Do you want to make a statement about runtimes?

>This has been amended.

Reviewer #3: The authors improved the presentation of the algorithm: I still believe that there is room for improvement, but I will no further complain about that.

HapCUT2 is reported not to support indel (Table 1, Table 2, Table 3), while it does support them as far as I can tell. I would ask the authors to double check their statement and possibly revise their conclusions (line 364, 370, 467, for example).

Evaluating haplotype assembly methods is hard, and I appreciate the details that the authors added in this revised version of their experimentation. However, I still find difficult to assess the relative merits of HaploMaker compared with HapCUT2 in the PacBio subreads and HiFi reads. Indeed, HaploMaker has higher MSE than HapCUT2 across all the intervals of haplotype sizes (see Figures 7 and 8). The data here seems to suggest that HaploMaker achieves a different trade-off than HapCUT2, with HaploMakers preferring reconstructing longer haplotypes and HapCUT2 being more conservative. This is perfectly fine and there could be applications where longer haplotypes (possibly switched) are preferable to shorted but more accurate haplotypes. However, in my opinion, the manuscript does not clearly present this fact in the relevant sections.
>We thank the reviewer for bringing this to our attention. We have managed to get HapCUT2 working with INDEL support and generated new results for all four experiments. We updated all relevant tables, Figures 5-8 and also amended relevant sections of manuscript text to reflect these result changes. Where relevant we have also added additional text discussing the potential trade-off from generating longer haplotypes at the sacrifice of having reduced accuracy.
Minor points:

Some links to GitHub files are still broken. Please double-check them.
>These broken links have been fixed.

Please add the license to the repository.
>The license file has been added to the GitHub repository and also mentioned in the manuscript.

Figure 4 is missing in the PDF I received.
>We apologise for this. This figure has now been submitted separately and is available to be viewed.

I wonder if paragraph starting at line 485 is more suited to the conclusion section.
>This paragraph is related to future research and seems more appropriate outside the formal conclusion section of the article.

Close
