# Supplementary material for: HaploMaker: An improved algorithm for rapid haplotype assembly of genomic sequences
Source: Gigascience. 2022 May 17;11:giac038. doi: 10.1093/gigascience/giac038 (PMC9112781; doi:10.1093/gigascience/giac038)
Supplement: giac038_Response_to_Reviewer_Comments_Revision_2 [file giac038_response_to_reviewer_comments_revision_2.pdf]

Author's Response To Reviewer Comments

Close

- Please move all URLs / dois /ftp sites /github repos referring to data sources, software etc. to the bibliography and cite them by number from the text. At GigaScience, we treat online data or software repositories and other internet resources as citable items, i.e. they should get their own numbered reference in the bibliography. Please refer to our instructions for authors for details.
>We have created citations for all the URL , github and software programs in the "Reference" section.

- Please also include your new GigaDB dataset in the bibliography and cite it by number from the availability section. (e.g. write "An archival copy of the code and supporting data is available via the GigaScience database, GigaDB (xx)".
>GigaDB citation was created and added.

Close
