# Supplementary material for: HaploMaker: An improved algorithm for rapid haplotype assembly of genomic sequences
Source: Gigascience. 2022 May 17;11:giac038. doi: 10.1093/gigascience/giac038 (PMC9112781; doi:10.1093/gigascience/giac038)
Supplement: giac038_Reviewer_1_Report_Original_Submission — Shilpa Garg -- 10/11/2021 Reviewed [file giac038_reviewer_1_report_original_submission.pdf]

Reviewer Report

Title: HaploMaker: An improved algorithm for rapid haplotype assembly of genomic sequences

Version: Original Submission **Date:** 10/11/2021

Reviewer name: Shilpa Garg

Reviewer Comments to Author:

In this article, the authors presented a novel reference-based haplotype assembly algorithm named HaploMiner in order to acquire long phased haplotypes with more accuracy. Importantly, this algorithm uses a directed acyclic graph structure to incorporate SNPs as nodes and weighted read evidences as edges, and converts the haplotype phasing task to an mathematical optimization problem. The authors then showed it worked well with both paired-end short reads and longer PacBio reads for NA12878 when compared with other algorithms. The idea of this algorithm is interesting, if its performance is solid, it would be promising to be further developed for much longer reads assembly.
Specific points:
1. In Page6 and line 89, I understand the authors want to point the protential ability of this algorithm, but they should change their statement in a more appropriate way. Since heterozygous positions are not always close, especially for species with large genome but low heterozygosity rate, and their algorithm are mainly designed for paired-end short reads assembly or pacbio reads, it would still be difficult to phase DNA strands "of any length". And at the results section, they didn't evaluate the algorithm on that, so it's not appropriate to say it "has the ability to phase DNA strands of any length …".
2. In Page15 and line 298, "HaploMiner, HapCompass and Hapcut2 comparably generated a similar number of haplotype blocks, 322K and 339K and 324K respectively … of 2770bp". The statement is conflict with Table 1. Not sure which one is correct. Please confirm and change it accordingly.
4. Since the haplotype path finding has been framed into a probability-based optimization problem that heavily based on the read evidences, so if the coverage depth will or will not influence the algorithm performance? and how? Please present experiments on algorithm performance with coverage.
5. The authors should provide experiments with PacBio HiFi data. See data available from paper: https://www.nature.com/articles/s41587-020-0711-0.
6. The authors didn't provide enough details about PacBio reads information, such as average mismatch rate, as they stated for short reads dataset. If provided, it will help the reader to know the quality of PacBio reads tested in this study.
7. The authors should perform experiments for other genomes that are used widely for phasing studies. See review article on genome and public datasets:
https://genomebiology.biomedcentral.com/articles/10.1186/s13059-021-02328-9
8. Please present the command lines used for experiments.
Decision: Major revision

Level of Interest

Please indicate how interesting you found the manuscript: Choose an item.

Quality of Written English

Please indicate the quality of language in the manuscript: Choose an item.

Declaration of Competing Interests

Please complete a declaration of competing interests, considering the following questions:

- Have you in the past five years received reimbursements, fees, funding, or salary from an organisation that may in any way gain or lose financially from the publication of this manuscript, either now or in the future?
- Do you hold any stocks or shares in an organisation that may in any way gain or lose financially from the publication of this manuscript, either now or in the future?
- Do you hold or are you currently applying for any patents relating to the content of the manuscript?
- Have you received reimbursements, fees, funding, or salary from an organization that holds or has applied for patents relating to the content of the manuscript?
- Do you have any other financial competing interests?
- Do you have any non-financial competing interests in relation to this paper?

If you can answer no to all of the above, write 'I declare that I have no competing interests' below. If your reply is yes to any, please give details below.

I declare that I have no competing interests

I agree to the open peer review policy of the journal. I understand that my name will be included on my report to the authors and, if the manuscript is accepted for publication, my named report including any attachments I upload will be posted on the website along with the authors' responses. I agree for my report to be made available under an Open Access Creative Commons CC-BY license (http://creativecommons.org/licenses/by/4.0/). I understand that any comments which I do not wish to be included in my named report can be included as confidential comments to the editors, which will not be published.

Choose an item.

To further support our reviewers, we have joined with Publons, where you can gain additional credit to further highlight your hard work (see: https://publons.com/journal/530/gigascience). On publication of this paper, your review will be automatically added to Publons, you can then choose whether or not to claim your Publons credit. I understand this statement.

Yes Choose an item.
