# Supplementary material for: HaploMaker: An improved algorithm for rapid haplotype assembly of genomic sequences
Source: Gigascience. 2022 May 17;11:giac038. doi: 10.1093/gigascience/giac038 (PMC9112781; doi:10.1093/gigascience/giac038)
Supplement: giac038_Reviewer_2_Report_Original_Submission — Alexander SchÃ¶nhuth D -- 10/20/2021 Reviewed [file giac038_reviewer_2_report_original_submission.pdf]

Reviewer Report

Title: HaploMaker: An improved algorithm for rapid haplotype assembly of genomic sequences

Version: Original Submission **Date:** 10/20/2021

Reviewer name: Alexander SchÃ¶nhuth

Reviewer Comments to Author:

This paper deals with haplotype assembly, which refers to reconstructing haplotypes from sequencing reads using applicable reference coordinates. Haplotype assembly partitions reads into groups reflecting their ancestral origin, and based on the grouping, infers the variants that characterize the ancestors. The bulk of work has been focusing on diploid genomes, and so does this paper. Haplotype assembly has been dealt with in various ways, many of which reflect a higher degree of sophistication in terms of computer science.
This paper presents a new framework based on directed acyclic graphs having clearly identifiable levels, allowing for nodes being (not just partially) ordered.
As for results in their own right, this paper does not appear to deliver any real progress. In fact, HaploMiner, the new approach presented is largely on a par with the state of the art approaches, with some advantages on the one hand, and some disadvantages on the other hand.
However, what intrigues me is the simplicity of the approach in comparison with earlier approaches, and the flexibility in terms of allowing for more than just two phases (haplotypes). Earlier approaches often are very sophisticated, which apparently seems to imply that earlier approaches cannot be flexibly extended to more than 2 haplotypes.
Given that, I am positive about this paper. I believe that the framework presented can deliver inspiration for future haplotype assembly approaches, also addressing polyploid settings.
What I found regrettable however was the lack of clarity in terms of sketching the computational problems and their algorithmic solutions in terms of disambiguous, formal notation. Given that the value of this paper (to the best of my understanding) is in the straightforwardness and simplicity of the approach, it would be great if the theoretically interested reader could clearly identify the computational problems and solutions involved, as per providing appropriate formal descriptions of the computational problems and

embedding them into the corresponding theory.
This does not mean that I do not appreciate the author's work in other ways: everything is perfectly reproducible for example, not lacking any clarity in this respect. I am only regretting that the theoretical / algorithmic concepts have not been embedded into the theoretical landscape of problems.
MAJOR:
* As already mentioned, an clearer embedding of the computational problems and solutions may be crucial to really understand the value of this paper.
* For example, what exactly do the optimization problems look like the algorithms behind HaploMiner are referring to, and how do they relate with known theoretical problems? (computing the "minimum weighted path" for example)
* What is the exact definition of a 'switch error' in this paper?
MINOR:
* "transforms the haplotype construction problem into a directed acyclic graph (DAG) structure" sounds strange (transforming a problem into a graph structure) What do you really mean?
* "The algorithm has the ability to phase DNA strands of any length as long as there are heterozygous positions close enough to be covered by at least a single DNA fragment." This is hard to believe (I actually don't), something is wrong here. Without having fragments connecting heterozygous positions, phasing is impossible. Having positions close enough is not sufficient.
* "the HaploMiner algorithm runs in O(n)": What is n actually defined to be?
* "processor power": what is that? (never heard that before)
* "HaploMiner algorithm was capable of constructing the most accurate haplotype blocks while maintaining length of haplotypes. Additionally, when using longer PacBio reads, HaploMiner generated significantly longer haplotype blocks while maintaining a competitively low switch error rate.": This is very imprecise summary. I would appreciate a clearer summary of achievements.
* "(a DNA read and its pair)": what is 'its pair' supposed to mean exactly?
* "if it spanned consecutive levels of the H-DAG without loss of coverage": what does 'loss of coverage' mean in this context?
* assignation -> assignment

Level of Interest

Please indicate how interesting you found the manuscript: Choose an item.

Quality of Written English

Please indicate the quality of language in the manuscript: Choose an item.

Declaration of Competing Interests

Please complete a declaration of competing interests, considering the following questions:

- Have you in the past five years received reimbursements, fees, funding, or salary from an organisation that may in any way gain or lose financially from the publication of this manuscript, either now or in the future?
- Do you hold any stocks or shares in an organisation that may in any way gain or lose financially from the publication of this manuscript, either now or in the future?
- Do you hold or are you currently applying for any patents relating to the content of the manuscript?
- Have you received reimbursements, fees, funding, or salary from an organization that holds or has applied for patents relating to the content of the manuscript?
- Do you have any other financial competing interests?
- Do you have any non-financial competing interests in relation to this paper?

If you can answer no to all of the above, write 'I declare that I have no competing interests' below. If your reply is yes to any, please give details below.

I declare that I have no competing interests

I agree to the open peer review policy of the journal. I understand that my name will be included on my report to the authors and, if the manuscript is accepted for publication, my named report including any attachments I upload will be posted on the website along with the authors' responses. I agree for my report to be made available under an Open Access Creative Commons CC-BY license (http://creativecommons.org/licenses/by/4.0/). I understand that any comments which I do not wish to be included in my named report can be included as confidential comments to the editors, which will not be published.

Choose an item.

To further support our reviewers, we have joined with Publons, where you can gain additional credit to further highlight your hard work (see: https://publons.com/journal/530/gigascience). On publication of this paper, your review will be automatically added to Publons, you can then choose whether or not to claim your Publons credit. I understand this statement.

Yes Choose an item.
