# Supplementary material for: HaploMaker: An improved algorithm for rapid haplotype assembly of genomic sequences
Source: Gigascience. 2022 May 17;11:giac038. doi: 10.1093/gigascience/giac038 (PMC9112781; doi:10.1093/gigascience/giac038)
Supplement: giac038_Reviewer_3_Report_Original_Submission — Yuri Pirola -- 10/21/2021 Reviewed [file giac038_reviewer_3_report_original_submission.pdf]

Reviewer Report

Title: HaploMaker: An improved algorithm for rapid haplotype assembly of genomic sequences

Version: Original Submission **Date:** 10/21/2021

Reviewer name: Yuri Pirola

Reviewer Comments to Author:

The authors present a new algorithm, called HaploMiner, for reconstructing haplotypes of a diploid individual based on sequenced reads. HaploMiner reduces the problem to the problem of finding a minimum weight path in a weighted DAG built using the input reads. Interesting is their approach that adds edges when a read presents evidence that two non-contiguous alleles are in-phase. The authors provide a basic experimental evaluation of their approach compared to other state-of-the-art approaches (HapCut2, HapCompass, WhatsHap). This assessment partially supports the claim that HaploMiner is competitive with the other approaches, while being significantly faster.
The computational problem is relevant, the exposition is generally clear, and the proposed approach, albeit algorithmically simple, is sound.
I have some major concerns and I think the authors should address them with a revision of the manuscript.
Formalization of the approach. Definitions of the structures used in HaploMiner are not provided in a formal and declarative way. For example, when the H-DAG is introduced (line 121 and following), the authors never says that each input variant site is associated with two nodes of the graph (with a level, using their terminology) and they say that edges only connect nodes of adjacent levels only in the following section. These parts are important in order to understand the approach and they can only be inferred by using the (useful) examples depicted in the figures. In general, I found that the exposition is more operational than declarative. For example, when weights are assigned to edges (line 223 and following) it is described how counters are computed rather than defining what the counters are. In this way, it is not clear if induced edges have counters equal to 1 or not. A definition would have made clear that.
Notation. The mathematical notations used in the manuscript are not the ones widely used in graph theory and/or they do not appear to be formally correct or consistent. For example, at line 124 the set of nodes is defined as set of L pairs, but edges are (presumably) defined over the elements that compose the pairs (and not the pairs themselves). Furthermore, I found rather strange that L is included in the triplet that defines the graph, as it can be deduced by the cardinality of the node set. Also, the notation for edges is new to me (see, for example, line 158): edges are generally represented as pairs, while the notation used in the manuscript usually represents functions/maps. Using the same capital letter for denoting sets and their elements (see, for example, the set of nodes - line 124 - or the set of paths -line 243) does not help readability. For a formal point of view, using Q_k and S_k to denote the likelihood and the negative log-likelihood of path P_k is an abuse of notation: using functions - q(P_k) and s(P_k), for example - is a more appropriate choice in my opinion. Carefully revising the notation would improve the readability of the manuscript.

Clarity/correctness of algorithm for inducing edges. I appreciate the idea of adding potential edges when there is indirect evidence that two non-adjacent alleles are in-phase (derived from paired-end reads, for example). However, I cannot fully understand the algorithm that has been proposed. I do understand the general idea, but I do not understand how the cases where read pairs intersect each other (say, a read pair connects level l with l+2, another read pair connects level l+1 with level l+3). Does the order of operations influence the final results? How the algorithm getPotentialChildren is applied? I think that this part is relevant for the novelty of the proposed approach, hence a more detailed and more formal description would improve the quality of the work.
Formal analysis of the time complexity. The authors claim that their approach runs in linear time (line 92-93). However, no formal analysis of the time complexity is presented. What represents n in line 92?
Limited experimental evaluation. The authors present a limited experimental evaluation of the approach. In particular:
- The results of the experiments on short reads are a bit disappointing. The input reads have an insert size of 336bp but the average reconstructed haplotype length is approximately 250bp (for all the methods). I wonder if the input data are sufficient to test the effectiveness of the haplotype reconstruction methods. Probably, checking coverages higher than 15x as used in the manuscript could help.
- On PacBio long reads, HaploMiner reconstructs longer haplotypes than the other methods but with a higher switch error rate. I wonder if the greater haplotype length is obtained since HaploMiner connects pairs of adjacent variant sites that the other methods instead "cut" since they think there is not enough evidence for phasing across these sites. In other words, the other methods might be more "conservative" than HaploMiner. To answer to this question, it would be interesting to investigate the distribution of the length of parts of haplotypes that do not contain switch errors (i.e., parts of haplotypes that were correctly phased).
- I also suggest adding the GATK HaplotypeCaller to the comparison, since it is based on a formulation different from the one on which the other methods are based. Such addition would better highlight the relative merits of the different approaches.
Minor points:
Please double check the links to the files hosted at GitHub, as some of them do not work.
Fig 5 and 6. Could you please also include (in a different figure/table) the number of haplotypes reconstructed by each method in each class? I think it is useful in order to avoid comparing means over sets of different sizes.
While the computational analysis is adequately described in the text and in the supplementary material, such description is not easily reproducible. Adding scripts for performing (either in the main repository or in a companion repo) the analysis and for recreating the environment (see, for example, conda) would further improve the work.

Level of Interest

Please indicate how interesting you found the manuscript: Choose an item.

Quality of Written English

Please indicate the quality of language in the manuscript: Choose an item.

Declaration of Competing Interests

Please complete a declaration of competing interests, considering the following questions:

- Have you in the past five years received reimbursements, fees, funding, or salary from an organisation that may in any way gain or lose financially from the publication of this manuscript, either now or in the future?
- Do you hold any stocks or shares in an organisation that may in any way gain or lose financially from the publication of this manuscript, either now or in the future?
- Do you hold or are you currently applying for any patents relating to the content of the manuscript?
- Have you received reimbursements, fees, funding, or salary from an organization that holds or has applied for patents relating to the content of the manuscript?
- Do you have any other financial competing interests?
- Do you have any non-financial competing interests in relation to this paper?

If you can answer no to all of the above, write 'I declare that I have no competing interests' below. If your reply is yes to any, please give details below.

I declare that I have no competing interests

I agree to the open peer review policy of the journal. I understand that my name will be included on my report to the authors and, if the manuscript is accepted for publication, my named report including any attachments I upload will be posted on the website along with the authors' responses. I agree for my report to be made available under an Open Access Creative Commons CC-BY license (http://creativecommons.org/licenses/by/4.0/). I understand that any comments which I do not wish to be included in my named report can be included as confidential comments to the editors, which will not be published.

Choose an item.

To further support our reviewers, we have joined with Publons, where you can gain additional credit to further highlight your hard work (see: https://publons.com/journal/530/gigascience). On publication of this paper, your review will be automatically added to Publons, you can then choose whether or not to claim your Publons credit. I understand this statement.

Yes Choose an item.
