# Supplementary material for: HaploMaker: An improved algorithm for rapid haplotype assembly of genomic sequences
Source: Gigascience. 2022 May 17;11:giac038. doi: 10.1093/gigascience/giac038 (PMC9112781; doi:10.1093/gigascience/giac038)
Supplement: giac038_Reviewer_3_Report_Revision_1 — Yuri Pirola -- 1/28/2022 Reviewed [file giac038_reviewer_3_report_revision_1.pdf]

Reviewer Report

Title: HaploMaker: An improved algorithm for rapid haplotype assembly of genomic sequences

Version: Revision 1 **Date:** 1/28/2022

Reviewer name: Yuri Pirola

Reviewer Comments to Author:

The authors improved the presentation of the algorithm: I still believe that there is room for improvement, but I will no further complain about that.
HapCUT2 is reported not to support indel (Table 1, Table 2, Table 3), while it does support them as far as I can tell. I would ask the authors to double check their statement and possibly revise their conclusions (line 364, 370, 467, for example).
Evaluating haplotype assembly methods is hard, and I appreciate the details that the authors added in this revised version of their experimentation. However, I still find difficult to assess the relative merits of HaploMaker compared with HapCUT2 in the PacBio subreads and HiFi reads. Indeed, HaploMaker has higher MSE than HapCUT2 across all the intervals of haplotype sizes (see Figures 7 and 8). The data here seems to suggest that HaploMaker achieves a different trade-off than HapCUT2, with HaploMakers preferring reconstructing longer haplotypes and HapCUT2 being more conservative. This is perfectly fine and there could be applications where longer haplotypes (possibly switched) are preferable to shorted but more accurate haplotypes. However, in my opinion, the manuscript does not clearly present this fact in the relevant sections.
Minor points:
Some links to GitHub files are still broken. Please double-check them.
Please add the license to the repository.
Figure 4 is missing in the PDF I received.
I wonder if paragraph starting at line 485 is more suited to the conclusion section.

Level of Interest

Please indicate how interesting you found the manuscript: Choose an item.

Quality of Written English

Please indicate the quality of language in the manuscript: Choose an item.

Declaration of Competing Interests

Please complete a declaration of competing interests, considering the following questions:

- Have you in the past five years received reimbursements, fees, funding, or salary from an organisation that may in any way gain or lose financially from the publication of this manuscript, either now or in the future?
- Do you hold any stocks or shares in an organisation that may in any way gain or lose financially from the publication of this manuscript, either now or in the future?
- Do you hold or are you currently applying for any patents relating to the content of the manuscript?
- Have you received reimbursements, fees, funding, or salary from an organization that holds or has applied for patents relating to the content of the manuscript?
- Do you have any other financial competing interests?
- Do you have any non-financial competing interests in relation to this paper?

If you can answer no to all of the above, write 'I declare that I have no competing interests' below. If your reply is yes to any, please give details below.

I declare that I have no competing interests

I agree to the open peer review policy of the journal. I understand that my name will be included on my report to the authors and, if the manuscript is accepted for publication, my named report including any attachments I upload will be posted on the website along with the authors' responses. I agree for my report to be made available under an Open Access Creative Commons CC-BY license (http://creativecommons.org/licenses/by/4.0/). I understand that any comments which I do not wish to be included in my named report can be included as confidential comments to the editors, which will not be published.

Choose an item.

To further support our reviewers, we have joined with Publons, where you can gain additional credit to further highlight your hard work (see: https://publons.com/journal/530/gigascience). On publication of this paper, your review will be automatically added to Publons, you can then choose whether or not to claim your Publons credit. I understand this statement.

Yes Choose an item.
